# Intracardiac Versus Transesophageal Echocardiography for Left Atrial Appendage Occlusion: A Meta-Analysis

**DOI:** 10.1016/j.jacadv.2026.102815

**Published:** 2026-05-20

**Authors:** Hatem Yaser, Shehab Yaser, Hazem E. Mohammed, George Hanen, Mohamed E. Haseeb, Mohamed Nasser, Elsayed S. Moubarak, Mira M. Abu Mahfouz, Mazen Negmeldin Yassin, AlMothana Manasrah, Islam Y. Elgendy, Ahmed Sami Abuzaid

**Affiliations:** a Faculty of Medicine, Assiut University, Assiut, Egypt; b Faculty of Medicine, Minia University, Minia, Egypt; c Medical Research Group of Egypt (MRGE), Negida Academy, Arlington, Massachusetts, USA; d Faculty of Medicine, Cairo University, Cairo, Egypt; e Faculty of Medicine, Yarmouk University, Irbid, Jordan; f United Health Services-Wilson Medical Center, Johnson City, New York, USA; g Division of Cardiovascular Medicine, Gill Heart and Vascular Institute, University of Kentucky, Lexington, Kentucky, USA; h Alaska Heart and Vascular Institute, Anchorage, Alaska, USA

**Keywords:** atrial fibrillation, intracardiac echocardiography, left atrial appendage occlusion, pericardial effusion, procedural time, transesophageal echocardiography

## Abstract

**Background:**

The optimal imaging modality for guiding left atrial appendage occlusion remains unclear.

**Objectives:**

The authors compared the effectiveness and safety of intracardiac echocardiography (ICE) vs transesophageal echocardiography (TEE) during left atrial appendage occlusion.

**Methods:**

Electronic databases were searched through November 2025 for studies that compared procedural success and safety with ICE vs TEE. We pooled data using a random-effects model to calculate ORs for categorical outcomes and mean differences (MDs) for continuous variables. A subgroup analysis was performed for unadjusted and adjusted data.

**Results:**

The meta-analysis included 27 observational studies, of which 9 studies reported adjusted summary estimates. Procedural success was comparable in both groups (unadjusted OR: 1.16; 95% CI: 0.92-1.45, I^2^ = 0%; adjusted OR: 1.40; 95% CI: 0.99-1.98, I^2^ = NA). ICE was associated with significantly shorter procedure duration in the adjusted analyses (MD -20.76 minutes; 95% CI: −26.99 to −14.53, I^2^ = 0%), though no difference was observed in unadjusted analyses (MD −0.13; 95% CI: −5.40 to 5.14, I^2^ = 95%). ICE was associated with a higher incidence of in-hospital pericardial effusion requiring intervention (unadjusted OR: 1.52; 95% CI: 1.13-2.04, I^2^ = 9%; adjusted OR: 1.74; 95% CI: 1.15-2.64, I^2^ = 8%). ICE was associated with a higher incidence of residual iatrogenic atrial septal defect at <4 months (unadjusted OR: 1.61; 95% CI: 1.20-2.16, I^2^ = 0%).

**Conclusions:**

ICE and TEE demonstrated comparable procedural success. However, ICE was associated with shorter procedural duration at the expense of a higher incidence of intervention-requiring pericardial effusion and residual iatrogenic atrial septal defect. (Intracardiac versus Transesophageal Echocardiography for Left Atrial Appendage Occlusion: A Meta-Analysis Stratified by Adjusted and Unadjusted Subgroups; CRD420251243170).

Atrial fibrillation (AF) is the most frequent cardiac arrhythmia in adults, carrying a significant risk of cardioembolic stroke primarily originating from the left atrial appendage (LAA).^1^, ^2^, ^3^, ^4^ While oral anticoagulants reduce systemic embolism, LAA occlusion (LAAO) has emerged as a valuable alternative, particularly for patients at high bleeding risk or those who are not candidates to systemic anticoagulation.^5^^,^^6^ Transesophageal echocardiography (TEE) is the gold standard imaging modality to guide LAAO and has been widely adopted in clinical practice. However, TEE has some notable limitations, such as discomfort, prolonged procedure time, and potential risk of esophageal trauma.^7^^,^^8^ An expert consensus has recently recommended the utilization of intracardiac echocardiography (ICE) as an alternative TEE for guiding LAAO, especially for AF patients at high risk for general anesthesia, due to shorter procedure durations and eliminating the need for general anesthesia.^9^^,^^10^ However, ICE remains underutilized in clinical practice due to the associated steep learning curve and widespread availability of TEE.^11^

Previous meta-analyses have reported comparable safety and effectiveness between TEE and ICE in guiding LAAO.^12^, ^13^, ^14^ However, many of these studies have not comprehensively addressed the long-term safety profiles of both imaging modalities. Additionally, prior meta-analyses combined unadjusted with adjusted data, which increases the risk of confounding bias and may produce skewed estimates of treatment effect.^15^ Therefore, we conducted this updated meta-analysis based on data adjustment to assess the comparative safety and effectiveness of both imaging modalities for guiding LAAO procedures.

## Methods

This meta-analysis of publicly available data was exempt from Institutional Review Board approval and patient consent. The study followed the Preferred Reporting Items for Systematic Reviews and Meta-Analyses guidelines and was registered with PROSPERO (CRD420251243170).^16^

### Eligibility criteria

Studies were included in our meta-analysis if they satisfied the following inclusion criteria: randomized or nonrandomized studies that involved adult patients (>18 years of age) with AF who underwent LAAO procedure and received either ICE or TEE. The studies were required to report comparative outcomes between ICE and TEE during LAAO. Non-English publications, case reports, conference abstracts, and single-arm studies were excluded.

### Information sources and search strategy

An electronic search document without any restrictions on year of publication was performed in Cochrane, PubMed, Scopus, and Web of Science through November 13, 2025. A combination of relevant search terms and Boolean operators based on our PICO criteria, such as “atrial fibrillation” AND “LAAO” AND “transesophageal echocardiograph∗” AND “intracardiac echocardiograph∗,” was used to obtain a comprehensive number of potentially eligible studies. Furthermore, backward and forward citation searching in eligible studies was carried out to ensure overarching inclusion of studies of interest. The full search strategy and number of retrieved records for each database are illustrated in (Supplemental Table 1).

### Selection process

Records collected from searching of electronic databases were imported into EndNote (Clarivate Analytics) for deduplication, and then Rayyan software for title/abstract screening and subsequent full-text screening of eligible articles.^17^^,^^18^ The screening process was performed independently by 2 authors. Disagreements between authors during the selection process were resolved by discussion with a senior author.

### Data collection and items

We extracted study design, patient characteristics, procedural details, and safety/effectiveness outcomes (defined as in-hospital, early [<4 months], or late [>4 months] follow-up). Six authors independently extracted data, with discrepancies resolved by a senior author. Data presented in a graphical form were extracted using plot digitization methods (Plot Digitizer, version 2.6.8, Free Software Foundation, Boston, MA). Categorical data were extracted as frequency of events and total number of participants in each study arm, while continuous outcomes were extracted as mean, SD, and total number of participants. Regarding continuous outcomes presented as median and IQRs, the mean and SD were approximated using the estimation method of Wan et al (2014).^19^

### Risk of bias assessment

The Risk Of Bias In Non-Randomized Studies - of Interventions (ROBINS-I) Version 2 tool was used to assess the risk of bias in the included observational studies.^20^ This tool evaluates the following 7 domains: confounding, selection of participants, classifications of interventions, deviations from intended interventions, missing data, measurement of outcomes, and selection of the reported result. The overall rating of “low,” “moderate,” “serious,” or “critical” risk of bias is assigned based on the highest risk of bias rating and its frequency across all domains. In accordance with the ROBINS-I tool prerequisites, we identified 4 confounding factors for evaluation:

• Clinical comorbidity burden, particularly the adjustment for key score estimates such as CHA_2_DS_2_-VASc and HAS-BLED scores and their components, given that higher-risk patients may be selected for ICE guidance to avoid general anesthesia.
• Operator or center experience, accounting for the ICE learning curve and its effect on procedure duration and safety.^21^, ^22^, ^23^
• Preprocedural imaging, controlling for anatomical complexity as assessed by baseline cardiac computed tomography or TEE.^6^
• Concomitant procedures, specifically catheter ablation, given its direct impact on procedural duration and guidance selection.^24^

The assessment was performed independently by 6 authors, and any discrepancies were resolved through consensus.

### Effect measures and data synthesis

For both unadjusted and adjusted analyses, categorical data were extracted as the number of events and total sample sizes, while continuous data were extracted as means and SDs. Subsequently, categorical data were pooled as ORs and continuous data as mean differences (MDs), each with their corresponding 95% CIs using a random-effects model. The DerSimonian–Laird estimator was uniformly applied for both categorical and continuous data. To estimate the expected range of true effects in future clinical settings, 95% prediction intervals (PIs) were calculated for all random-effects models. Heterogeneity was assessed using Cochrane’s Q test, with an I^2^ value of >50% and a *P* value of <0.1 being indicative of significant heterogeneity. Differences between subgroups were explicitly evaluated using Cochran’s Q test for subgroup differences to generate a *P* value.

For outcomes exhibiting significant heterogeneity, a leave-one-out sensitivity analysis was iteratively performed to evaluate the robustness of the pooled results and ascertain if any single study disproportionately influenced the overall effect sizes. Furthermore, to account for baseline confounding variables, a prespecified subgroup analysis was conducted by stratifying the pooled studies into unadjusted and adjusted cohorts. The adjusted subgroup consisted specifically of pooled raw data from propensity score matched or inverse probability weighted cohorts. Due to the limited number of studies reporting adjusted analyses, subsequent meta-regression and publication bias assessments were restricted exclusively to the unadjusted subgroup. Mixed-effects meta-regression analyses, utilizing both additive and interactive models, were conducted to examine the impact of baseline CHA2DS2-VASc and HAS-BLED scores on the pooled effect sizes. The interactive model was specifically employed to assess whether the concurrent elevation of both risk scores significantly modifies procedural outcomes differently than their independent additive effects. Finally, potential publication bias was assessed visually using funnel plots and statistically quantified using Egger’s test. Review Manager (RevMan) [Version 5.4] and RStudio 2026.01.2 + 418 were used for the statistical analysis of pooled data.

## Results

### Literature search

**Figure 1:**
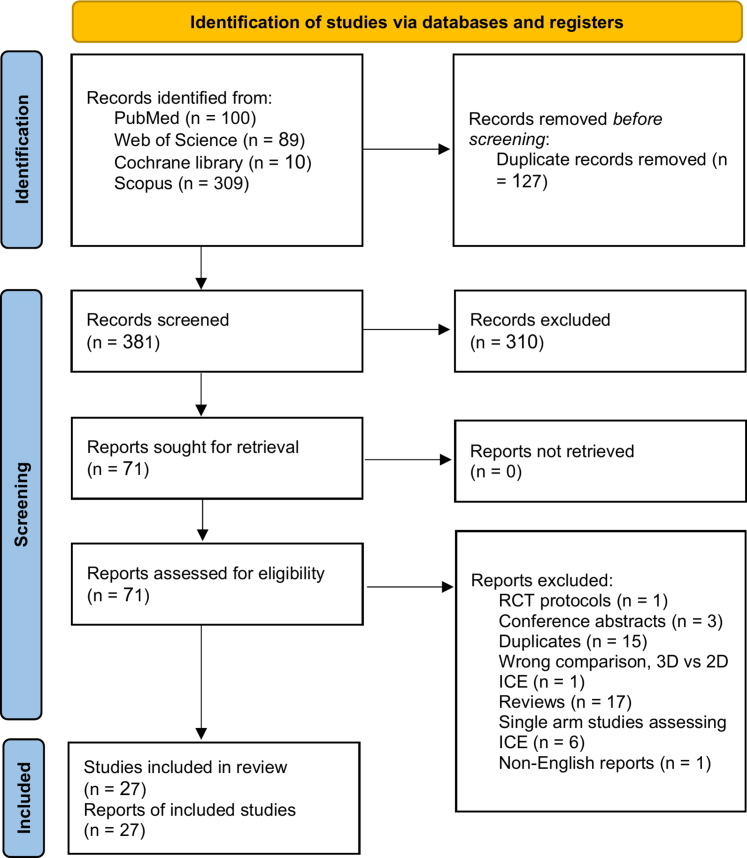
PRISMA Flow Diagram Preferred Reporting Items for Systematic Reviews and Meta-Analyses flow diagram of study screening and selection. ICE = intracardiac echocardiography; 2D = 2-dimensional; 3D = 3-dimensional; RCT = randomized controlled trials.

A total of 508 records were initially identified. Seventy-one full-text reports were assessed for eligibility, of which 44 were excluded. Ultimately, 27 studies met the inclusion criteria and were included in the final qualitative synthesis (Figure 1).^11^^,^^21^, ^22^, ^23^^,^^25^, ^26^, ^27^, ^28^, ^29^, ^30^, ^31^, ^32^, ^33^, ^34^, ^35^, ^36^, ^37^, ^38^, ^39^, ^40^, ^41^, ^42^, ^43^, ^44^, ^45^, ^46^, ^47^

### Studies and population characteristics

Included studies encompassed prospective and retrospective cohorts enrolling patients from 2008 to 2024, primarily utilizing Watchman or Amulet devices (Table 1). Patients were generally elderly in the eighth decade of life with high thromboembolic (CHA_2_DS_2_-VASc 4-5) and bleeding risks (Table 2).

**Table 1:** Summary of Included Studies

First Author, Year	Cohort Design	Number of Centers	Country	Imaging Follow-Up	Clinical Follow-Up	Type of Device Used	Procedural/Technical Success Definition	Major Bleeding Definition/Criteria	Enrollment Period
Adams, 2024	Retrospective	1	United States	NA	NA	Watchman FLX/Amulet	A successful LAA implant was defined as complete LAA occlusion	NA	January 2022–March 2023
Alkhouli, 2020	Prospective	1	United States	45 ± 15 d	45 ± 15 d	Watchman	Ability to successfully implant a device in the left atrial appendage	NA	June 2016–April 2019
Alkhouli, 2025	Retrospective	Multicenter	United States	45 ± 14 d	45 d	Amulet	Implantation success was defined as successful deployment of the Amulet occluder and completion of procedure after venous access was obtained	Defined as pericardial effusion requiring surgery, access site bleeding, hematoma, gastrointestinal bleeding, hemothorax requiring drainage or hospitalization, nonintracranial hemorrhage, or retroperitoneal bleeding	August 2021–December 2023
Berti, 2018	Retrospective	16	Italy	NA	NA	ACP/Amulet	Successful deployment and implantation of the device without major procedure-related complications.	International Society on Thrombosis and Haemostasis criteria	December 2008–April 2015
Chen, 2023	Prospective	1	China	45 d to 3 mo	3 mo	LAmbre	NA	NA	April 2019–February 2022
Chu, 2020	Prospective	1	China	NA	NA	LAmbre	NA	NA	April 2019–June 2019
Ferro, 2023	Retrospective	698	United States	45 d	45 d	Watchman FLX	Implant success, defined as device release and deployment, was also assessed.	NA	August 2020–September 2021
Frangieh, 2017	Prospective	1	Switzerland	NA	NA	Watchman	NA	NA	November 2013–June 2016
Gianni, 2021	Prospective	1	United States	Median of 8 (7-11) wk	NA	Watchman FLX	NA	NA	August–December 2020
Grazina, 2023	Retrospective	1	Portugal	45 d	1 y	ACP/Amulet/Watchman/LAmbre	NA	Bleeding Academic Research Consortium criteria (types 2---5)	2009–2020
Hemam, 2019	Retrospective	3	United States and Colombia	45 or 120 d (depending on center)	NA	Watchman	NA	NA	April 2015–January 2018
Kim, 2018	Retrospective	2	South Korea	NA	NA	ACP/Amulet/Watchman	NA	NA	March 2013–April 2017
Korsholm, 2017	Retrospective	1	Denmark	8 wk	8 wk	ACP/Amulet	Technical success without procedure-related complications.	NA	March 2010–November 2016
Korsholm, 2020	Prospective	1	Denmark	NA	NA	Watchman	NA	Bleeding Academic Research Consortium type 3 or higher	March 2019–January 2020
Kumar, 2025	Retrospective	1	United States	45 d	45 d	Watchman	Successful deployment of the LAA occluder and absence of periprocedural complications	NA	2021–2024
Liu, 2024	Prospective	1	China	3 mo	18.46 ± 7.70 mo	Watchman/LAmbre	NA	NA	November 2017–May 2022
Morcos, 2022	Retrospective	Multicenter	United States	NA	NA	Watchman/Amulet	NA	NA	2016–2018
Nielsen-Kudsk, 2019	Prospective	13	International/global registry	NA	1 y	Amulet	NA	Bleeding Academic Research Consortium type 3 or higher	June 2015–September 2016
Pastormerlo, 2023	Prospective	26	Italy	NA	1 y	Watchmann FLX	Procedural success was defined as technical success without major procedure-related complications. Fully meeting the PASS criteria. Procedural success: technical success without major procedure-related complications	International Society on Thrombosis and Haemostasis criteria	October 2018–September 2021
Pommier, 2021	Prospective	1	France	6-8 wk	Median 326 d	ACP/Watchman	Procedural success was defined according to the Munich consensus, which specifies that the device should be implanted in the correct position without device-related complications and no peridevice leaks >5 mm on color Doppler.	NA	January 2014–April 2019
Puga, 2021	Retrospective	1	Portugal	30 d	Median 14.5 mo	Amulet/ACP/Watchman	NA	NA	May 2010–August 2017
Reis, 2018	Retrospective	1	Portugal	6 mo	NA	ACP/Watchman	NA	Defined as the occurrence of intracranial hemorrhage or blood loss associated with a decrease in hemoglobin ≥5 g/dL.	May 2010–January 2017
Shang, 2023	Prospective	1	China	3 mo	15.67 ± 3.76 mo (ICE group) and 16.49 ± 2.88 mo (TEE arm)	Watchman	The procedure success was defined as no peri-device leak (PDL) > 5 mm on ultrasound images, no device-related complications, and no procedure-related complications	NA	January 2021–October 2022
Stout, 2024	Prospective	1	United States	1 y	1 y	Watchman/Watchman FL	Successful implantation without conversion to TEE, no peridevice leak and no procedural complications	NA	January 2018 to March 2022
Streb, 2019	Prospective	1	Poland	30 d	30 d	Amulet	NA	NA	NA
Su, 2022	Prospective	39	China	30 d	30 d	Watchman	Device deployed and implanted in the correct position.	Major/life-threatening bleeding defined by Munich consensus and BARC criteria	April 2019–October 2020
Zahid, 2022	Retrospective	NA	United States	NA	NA	Watchman	NA	Bleeding complications (retroperitoneal and gastrointestinal) identified using ICD-10 codes	Quarter 4 of 2015–2019

ACP = Amplatzer cardiac plug; BARC = Bleeding Academic Research Consortium; ICD = International Classification of Diseases; ICE = intracardiac echocardiography; LAA = left atrial appendage; NA = not available; PASS = Position: device at the LAA ostium; Anchor: fixation anchors engaged/stable device; Size: 8% to 20% device compression; Seal: device spans the ostium, all lobes covered; TEE = transesophageal echocardiography.

**Table 2:** Baseline Characteristics of Participants in Included Studies

First Author, Year	No. of Patients		Age (Years), Mean (SD)		Sex (Female), n (%)		Preprocedural Imaging, n (%)		Classification of AF, n (%)		Comorbidities, n (%)		CHA2DS2-VASc Score, Mean (SD)		HAS-BLED Score, Mean (SD)		LVEF (c/0), Mean (SD)	
	ICE	TEE	ICE	TEE	ICE	TEE	ICE	TEE	ICE	TEE	ICE	TEE	ICE	TEE	ICE	TEE	ICE	TEE
Adams, 2024	46	75	76.4 (6.7)	76.1 (7.2)	16 (34.8)	31 (41.3)	NA	NA	NA	NA	HTN: NA DM: NA HF: 19 (41.3) Previous stroke/TIA: NA	HTN: NA DM: NA HF: 24 (32.0) Previous stroke/TIA: NA	4.1 (1.2)	4.3 (1.4)	4.3 (1)	3 (0.81)	NA	NA
Alkhouli, 2020	90	196	75.7 (8)	75.2 (7.8)	34 (37.8)	87 (44.4)	CTA: 28 (31.1) TEE: 54 (60)	CTA: 27 (13.8) TEE: 160 (81.6)	NA	NA	HTN: 83 (92.2) DM: 30 (33.3) HF: 51 (56.7) Previous stroke/TIA: 33 (36.5)	HTN: 171 (87.2) DM: 86 (43.9) HF: 95 (48.5) Previous stroke/TIA: 84 (42.9)	4.7 (1.4)	4.8 (1.6)	2.8 (1.2)	2.9 (1.1)	55.3 (11.6)	58 (9.1)
Alkhouli, 2025	433	9,793	76.5 (7.7)	77.1 (7.7)	158 (36.5)	3,927 (40.1)	CTA/TEE: 191 (44.1)	CTA/TEE: 2,673 (27.3)	Paroxysmal: 236 (54.5) Persistent/permanent: 197 (45.5)	Paroxysmal: 6,228 (63.6) Persistent/permanent: 3,565 (36.4)	HTN: 396 (91.5) DM: 152 (35.1) HF: NA Previous stroke/TIA: 89 (20.6)	HTN: 8,970 (91.6) DM: 3,300 (33.7) HF: NA Previous stroke/TIA: 1,978 (20.2)	4.6 (1.6)	4.7 (1.5)	2.7 (1)	2.7 (1.1)	53.7 (9.6)	54.4 (9.4)
Berti, 2018	187	417	76 (8)	74 (7)	64 (34)	146 (35)	CTA: 138 (74) TEE: 82 (44)	CTA: 125 (30) TEE: 379 (91)	Permanent: 127 (68)	Permanent: 267 (64)	HTN: NA DM: NA HF: NA Previous stroke/TIA: NA	HTN: NA DM: NA HF: NA Previous stroke/TIA: NA	4.27 (1.4)	4.25 (1.4)	3.25 (1)	3.15 (1.1)	53 (9)	52 (11)
Chen, 2023	69	121	73 (8.3)	70.8 (7.3)	19 (27.5)	41 (33.9)	TEE: 187 (100)	TEE: 417 (100)	Paroxysmal: 4 (5.8) Persistent/long-standing persistent/permanent: 65 (94.2)	Paroxysmal: 3 (2.5) Persistent/long-standing persistent/permanent: 118 (97.5)	HTN: 49 (71.0) DM: 19 (27.5) HF: NA Previous stroke/TIA: 37 (53.6)	HTN: 100 (82.6) DM: 34 (28.1) HF: NA Previous stroke/TIA: 68 (56.2)	4.4 (1.7)	4.4 (1)	2.6 (1)	2.9 (1.1)	62 (9.9)	62.3 (9.1)
Chu, 2020	7	7	71.7 (8.8)	75.6 (9.1)	2 (28.6)	3 (42.9)	NA	NA	Paroxysmal: 4 (57.1)	Paroxysmal: 3 (42.9)	HTN: 5 (71.4) DM: 0 (0) HF: NA Previous stroke/TIA: 1 (14.3)	HTN: 6 (85.7) DM: 1 (14.3) HF: NA Previous stroke/TIA: 0 (0)	5.1 (2.1)	5.1 (1.2)	3.0 (1.2)	3.1 (0.7)	60.9 (10.6)	64.3 (5.4)
Ferro, 2023	2,272	31,835	75.8 (8.0)	76.4 (7.9)	907 (39.9)	13,018 (40.9)	CTA: 864 (38.1)	CTA: 6,298 (19.8)	Paroxysmal: 1,257 (55.7) Persistent: 599 (26.5) Long-standing persistent/permanent: 402 (17.9)	Paroxysmal: 19,735 (62.5) Persistent: 6,076 (19.2) Long-standing persistent/permanent: 5,781 (18.3)	HTN: 2,083 (91.7) DM: 837 (36.9) HF: 791 (34.8) Previous stroke/TIA: 524 (23.1)	HTN: 29,194 (91.7) DM: 11,350 (35.7) HF: 12,449 (39.1) Previous stroke/TIA: 6,851 (21.5)	4.8 (1.5)	4.8 (1.5)	2.5 (1)	2.4 (1.0)	54.3 (10.2)	54.0 (9.9)
Frangieh, 2017	32	44	74.67 (9.31)	80.33 (7.66)	6 (18.75)	19 (43.18)	TEE: 76 (100)		Permanent: 10 (31)	Permanent: 20 (46)	HTN: 27 (84) DM: 14 (44) HF: NA Previous stroke/TIA: 9 (28)	HTN: 38 (86) DM: 16 (36) HF: NA Previous stroke/TIA: 9 (21)	4.27 (2.17)	4 (1.53)	3.33 (0.78)	3.6 (1.38)	52.67 (14.74)	59.33 (6.13)
Gianni, 2021	122	68	72 (8)	75 (9)	41 (34)	27 (40)	NA	NA	NA	NA	HTN: NA DM: NA HF: NA Previous stroke/TIA: NA	HTN: NA DM: NA HF: NA Previous stroke/TIA: NA	4.1 (1.4)	4.3 (1.3)	2.7 (1.3)	2.7 (1.2)	NA	NA
Grazina, 2023	45	43	75.5 (9.6)	74.2 (9.8)	13 (28.9)	15 (34.9)	CTA/TEE: 45 (100)	CTA/TEE: 43 (100)	Permanent: 31 (68.9)	Permanent: 32 (74.4)	HTN: 31 (68.9) DM: 15 (33.3) HF: NA Previous stroke/TIA: NA	HTN: 36 (83.7) DM: 13 (30.2) HF: NA Previous stroke/TIA: NA	4.0 (1.4)	4.1 (1.4)	3.6 (1.1)	3.6 (1)	NA	NA
Hemam, 2019	53	51	77 (10)	76 (7)	20 (37.7)	20 (39.2)	NA	NA	NA	NA	HTN: 43 (81) DM: 18 (34) HF: 10 (19) Previous stroke/TIA: 22 (42)	HTN: 46 (90) DM: 15 (29) HF: 13 (25) Previous stroke/TIA: 17 (33)	NA	NA	NA	NA	NA	NA
Kim, 2018	41	103	71.4 (9.3)	72.3 (9.2)	17 (41.5)	51 (50.5)	TEE: 41 (100)	TEE: 103 (100)	Paroxysmal: 14 (34.1)	Paroxysmal: 28 (27.2)	HTN: 37 (90.2) DM: 11 (26.8) HF: NA Previous stroke/TIA: 20 (48.8)	HTN: 86 (83.5) DM: 26 (25.2) HF: NA Previous stroke/TIA: 44 (42.7)	4.3 (1.4)	4.3 (1.4)	3.0 (1.5)	3.1 (1.4)	NA	NA
Korsholm, 2017	109	107	73.0 (7.8)	73.0 (9.7)	41 (38)	28 (26)	CTA: 109 (100) TEE: 8 (7)	CTA: 107 (100) TEE: 0	Paroxysmal: 52 (48) Persistent: 7 (6) Permanent: 50 (46)	Paroxysmal: 45 (42) Persistent: 8 (8) Permanent: 54 (50)	HTN: 91 (83) DM: 23 (21) HF: 16 (15) Previous stroke/TIA: 50 (46)	HTN: 86 (80) DM: 23 (22) HF: 21 (20) Previous stroke/TIA: 59 (55)	4.1 (1.6)	4.4 (1.6)	4.1 (0.9)	4.1 (1.1)	NA	NA
Korsholm, 2020	91		73.3 (8.5)		68 (75)		CTA: 91 (100)		Permanent: 45 (49)		HTN: 69 (76) DM: 24 (26) HF: 15 (16) Previous stroke/TIA: 38 (42)		3.9 (1.7)		2.4 (1)		56.6 (7.5)	
Kumar, 2025	46	97	75.1 (6.4)	76.8 (5.6)	11 (23.9)	40 (41.2)	CTA: 46 (100)	TEE: 97 (100)	NA	NA	HTN: 44 (95.7) DM: 17 (37.0) HF: NA Previous stroke/TIA: 9 (19.6)	HTN: 86 (88.7) DM: 29 (29.9) HF: NA Previous stroke/TIA: 22 (22.7)	4.3 (1.3)	3.9 (1.4)	3.7 (0.9)	3.4 (0.8)	60.6 (8.5)	58.4 (8.1)
Liu, 2024	132	132	62.98 (8.14)	62.44 (8.52)	56 (42.42)	52 (39.39)	CTA: 132 (100) TEE: 132 (100)	CTA: 132 (100) TEE: 132 (100)	Paroxysmal: 36 (27.27) Persistent/long-standing persistent: 96 (72.73)	Paroxysmal: 23 (17.42) Persistent/long-standing persistent: 109 (82.58)	HTN: 83 (62.88) DM: 37 (28.03) HF: 85 (64.39) Previous stroke/TIA: 71 (53.79)	HTN: 88 (66.67) DM: 26 (19.70) HF: 98 (74.24) Previous stroke/TIA: 70 (53.03)	4.33 (1.44)	4.26 (1.42)	2.80 (0.80)	2.77 (0.95)	NA	NA
Morcos, 2022	396	18,050	70.7 (13.93)	75.3 (188.09)	162 (40.8)	7,213 (40.0)	NA	NA	Paroxysmal: 160 (40.3) Unspecified: 237 (59.7)	Paroxysmal: 8,501 (47.1) Unspecified: 9,550 (52.9)	HTN: 234 (59.2) DM: 68 (17.1) HF: 10 (2.5) Previous stroke/TIA: NA	HTN: 219 (55.4) DM: 66 (16.8) HF: 5 (1.3) Previous stroke/TIA: NA	NA	NA	NA	NA	NA	NA
Nielsen-Kudsk, 2019	130	955	75 (8)	75 (9)	52 (40)	335 (35)	NA	NA	NA	NA	HTN: NA DM: NA HF: NA Previous stroke/TIA: Stroke: 42 TIA: 12	HTN: NA DM: NA HF: NA Previous stroke/TIA: Stroke: 25,TIA: 10	4.1 (1.6)	4.2 (1.6)	3.2 (0.9)	3.3 (1.1)	NA	NA
Pastormerlo, 2023	149	623	77 (7.5)	76.3 (8)	52 (35)	216 (35)	CTA: 140 (94) TEE: 12 (8)	CTA: 62 (10) TEE: 567 (91)	Permanent: 72 (48)	Permanent: 304 (48)	HTN: 115 (77) DM: 45 (30) HF: NA Previous stroke/TIA: 19 (12)	HTN: 491 (78) DM: 219 (34) HF: NA Previous stroke/TIA: 87 (13)	4.2 (1.8)	4.1 (1.4)	3.5 (1.4)	3.7 (1.1)	54 (11)	51 (11)
Pommier, 2021	175	49	76 (8)	75 (7)	53 (30)	14 (27)	CTA: 175 (100)	CTA: 49 (100)	Paroxysmal: 51 (29) Permanent: 122 (70)	Paroxysmal: 11 (23) Permanent: 37 (77)	HTN: 160 (91) DM: 60 (34) HF: 30 (17) Previous stroke/TIA: 122 (70)	HTN: 46 (96) DM: 10 (21) HF: 8 (17) Previous stroke/TIA: 31 (64)	4.2 (1.38)	4.5 (1.49)	4.07 (0.99)	3.93 (1.02)	57 (7)	57 (7)
Puga, 2021	30	36	72.1 (9)		23 (32.9)		TEE: 30 (100)	TEE: 36 (100)	Paroxysmal: 18 (25.7)		HTN: 26 (37.1) DM: 26 (37.1) HF: NA Previous stroke/TIA: NA		3 (1.51)		2.67 (0.76)		55 (9.6)	
Reis, 2018	26	56	74 (8.0)		29 (35.4)		TEE: 26 (100)	TEE: 56 (100)	Paroxysmal: 25 (30.5) Persistent: 4 (4.9) Permanent: 53 (64.6)		HTN: 71 (86.6) DM: 26 (31.7) HF: NA Previous stroke/TIA: 34 (41.5)		4.7 (1.4)		3.3 (1)		NA	
Shang, 2023	193	109	65.02 (8.47)	64.23 (7.75)	84 (43.5)	40 (36.7)	NA	NA	Paroxysmal: 95 (49.22)	Paroxysmal: 44 (40.37)	HTN: 114 (59.07) DM: 61 (31.61) HF: 79 (40.93) Previous stroke/TIA: 98 (50.78)	HTN: 71 (65.14) DM: 27 (24.77) HF: 39 (35.78) Previous stroke/TIA: 46 (42.20)	3.87 (1.60)	3.41 (1.82)	2.19 (1.15)	2.07 (1.28)	58 (9)	58 (8)
Stout, 2024	63	171	76 (8)	76 (8)	25 (39.7)	74 (43.3)	NA	NA	Paroxysmal: 51 (81) Long-standing persistent: 0 Permanent: 11 (17.5)	Paroxysmal: 113 (66.9) Long-standing persistent: 3 (1.8) Permanent: 29 (17.2)	HTN: 61 (97.6) DM: 27 (42.9) HF: 24 (38.1) Previous stroke/TIA: 9 (14.3)	HTN: 166 (97.1) DM: 75 (43.9) HF: 76 (44.4) Previous stroke/TIA: 22 (12.9)	5	5.1	NA	NA	NA	NA
Streb, 2019	11	12	31.67 (12.72)	33 (5.87)	7 (63.6)	7 (54.5)	TEE: 100	TEE: 100	Paroxysmal: 5 (45.5)	Paroxysmal: 8 (66.7)	HTN: 9 (81.8) DM: 3 (27.3) HF: 2 (20.0) Previous stroke/TIA: 5 (45.5)	HTN: 11 (91.7) DM: 3 (25.0) HF: 4 (33.3) Previous stroke/TIA: 3 (25.0)	5 (1.27)	5 (1.26)	3 (0.85)	2 (0.42)	53.5 (16.5)	55.0 (14.0)
Su, 2022	95	2,508	69.1 (9.4)	69.1 (9.4)	1,314 (42.4)	1,314 (42.4)	CTA: 1,812 (58.5) TEE: 1,756 (56.7)	CTA: 1,812 (58.5) TEE: 1,756 (56.7)	Paroxysmal: 1,249 (40.3) Persistent: 1,277 (41.2) Long-standing persistent: 570 (18.4)	Paroxysmal: 1,249 (40.3) Persistent: 1,277 (41.2) Long-standing persistent: 570 (18.4)	HTN: 2,130 (68.8) DM: 720 (23.3) HF: 462 (15.0) Previous stroke/TIA: 1,418 (45.9)	HTN: 2,130 (68.8) DM: 720 (23.3) HF: 462 (15.0) Previous stroke/TIA: 1,418 (45.9)	4.0 (1.8)	4.0 (1.8)	2.4 (1.2)	2.4 (1.2)	60.0 (8.3)	60.0 (8.3)
Zahid, 2022	1,410	60,585	74.33 (7.42)	76.67 (8.15)	540 (38.3)	25,270 (41.7)	NA	NA	NA	NA	HTN: 1,215 (86.2) DM: 290 (20.6) HF: 415 (29.4) Previous stroke/TIA: NA	HTN: 52,575 (86.8) DM: 11,370 (18.8) HF: 20,910 (34.5) Previous stroke/TIA: NA	NA	NA	NA	NA	NA	NA

AF = atrial fibrillation; CTA = computed tomography angiography; DM = diabetes mellitus; HF = heart failure; HTN = hypertension; LVEF = left ventricular ejection fraction; TIA = transient ischemic attack; other abbreviations as in Table 1.

### Quality assessment

**Figure 2:**
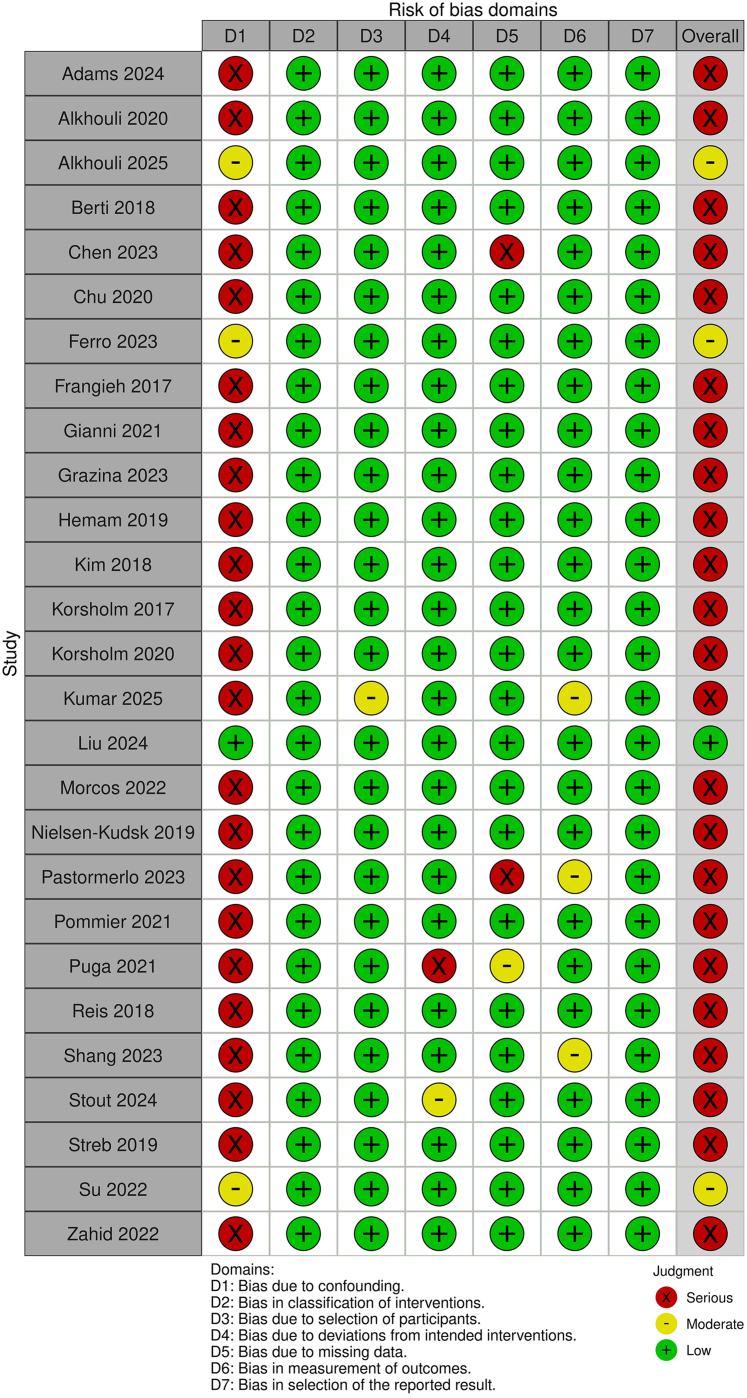
Risk of Bias Assessment Quality assessment of nonrandomized studies using the ROBINS-I V2 tool. ROBINS-I = Risk Of Bias In Non-Randomized Studies - of Interventions.

Considerable heterogeneity was observed in the handling of confounding across the included studies. Adjustment for the prespecified confounding factors varied substantially (Table 3). Using the ROBINS-I version 2 tool, bias due to confounding (Domain 1) emerged as the predominant source of bias and the principal driver of serious overall risk-of-bias judgments. Liu et al (2024) was judged as low risk of bias, and only 3 studies were judged to have a *moderate* risk of bias due to confounding—Alkhouli et al (2025), which did not adjust for operator or center volume, and Ferro et al (2023) and Su et al. (2022), which did not account for preprocedural imaging—while all remaining studies were classified as having a *serious* risk of bias due to incomplete or absent adjustment for multiple key confounders (Figure 2).^21^, ^22^, ^23^^,^^38^

**Table 3:** Adjustment Method Details

	Alkhouli, 2025	Chu, 2020	Ferro, 2023	Kim, 2018	Liu, 2024	Morcos, 2022	Puga, 2021	Su, 2022	Zahid, 2022
Adjustment analysis used	Multivariable logistic regression	Propensity score matching	Inverse probability weighting	Univariate and multivariate linear regression	Propensity score matching	Propensity score matching	Multivariate analysis	Univariable and multivariable logistic regression	Propensity score matching
Sample size included	ICE: 433 TEE: 9,793	ICE: 7 TEE: 7	ICE: 1,408 TEE: 22,159	ICE: 41 TEE: 103	ICE: 132 TEE: 132	ICE: 395 TEE: 395	ICE: 30 TEE: 36	ICE: 95 TEE: 2,508	ICE: 1,410 TEE: 1,410
Adjusted outcomes	Safety composite endpoint, in-hospital adverse events and clinical events through 45 d	Procedure time, fluoroscopy time (zero in ICE group), fluoroscopy dose, contrast consumption, successful implantation, complete occlusion and peridevice leak	Effectiveness and safety endpoints, in-hospital and at 45 d	Total procedural time	Procedure-related events and follow-up results	In-hospital complications and resources limitation	iASD	Procedural success, any life-threatening or major bleeding at 30 d and death, stroke, or systemic embolism at 30 d	Hospital encounter outcomes and resource utilization
Preliminary consideration of confounding factors									
Risk scores (CHA2DS2-VASc, HAS-BLED) and their components	Adjusted	NA	Adjusted	Unadjusted	Adjusted	Unadjusted	Unadjusted	Adjusted	Unadjusted
Operator or center experience	Unadjusted	NA	Adjusted	Unadjusted	A single, highly experienced staff member was involved in the investigation	Unadjusted	Unadjusted	Adjusted	Unadjusted
Preprocedural imaging	Adjusted	NA	Unadjusted	All included patients had preprocedural TEE	All included patients had both preprocedural CTA and TEE	Unadjusted	All included patients had preprocedural TEE	Unadjusted	Unadjusted
Concomitant procedures	Patients with combined procedures were excluded	NA	Patients with combined procedures were excluded	Unadjusted	All included patients had combined catheter ablation	Unadjusted	Unadjusted	Adjusted	Unadjusted
Overall variables used in adjustment	AF classification, hypertension, HAS-BLED score, prior bleeding, increased fall risk, prior LAAO intervention, baseline imaging performed, age, CHA2DS2-VASc score	NA	Age (continuous), sex, body mass index (continuous), race and ethnicity (White vs other), AF classification (paroxysmal vs other), chronic lung disease, sleep apnea, cardiomyopathy, coronary artery disease, clinically relevant bleeding, increased risk of falls, CHA2DS2-VAsc, components (congestive heart failure, hypertension, diabetes mellitus, stroke/TIA, vascular disease), HAS-BLED, components (abnormal renal function, abnormal liver function, labile INR, alcohol, antiplatelet drug use, NSAID use), periprocedural P2Y12 inhibitor, periprocedural oral anticoagulant, prior coronary bypass surgery, prior structural cardiac surgery, prior percutaneous angioplasty, prior arrhythmia ablation, site volume (cases, continuous), operator volume (cases, continuous)	Age, male, ICE, time period (A, B, C), type of device (Amplatzer Cardiac Plug, Amulet, Watchman), type of anesthesia (general anesthesia, local anesthesia with sedation)	Age, gender, CHA2DS2-VASc score, HAS-BLED score	Age, sex, valvular disease, diabetes, hypothyroidism, coagulopathy, obesity, chronic blood loss anemia, alcohol abuse, drug abuse, congestive heart failure, pulmonary circulation disease, peripheral vascular disease, chronic pulmonary disease, liver disease, hypertension, coronary artery disease, chronic kidney disease, patient location (NCHS urban-rural code), median household income national quartile for patient ZIP code, all patient refined DRG (risk of mortality subclass), all patient refined DRG (severity of illness subclass), bed size of hospital, control/ownership of hospital, hospital urban-rural designation, teaching status of urban hospitals	Device size, type of device (Watchman vs ACP/Amulet), presence of moderate or severe mitral regurgitation, exclusive use of ICE	Demographic characteristics (age, sex), coexisting medical conditions (diabetes, hypertension, prior coronary artery disease, prior vascular disease, previous stroke, chronic heart failure), type of anesthesia, type of intraprocedural imaging guidance, combined procedure, the center volume, HAS-BLED score (calculated as hypertension, abnormal kidney or liver function, stroke, bleeding history or predisposition, labile international normalized ratio, elderly [ie, aged >65 years], and using drugs or alcohol concomitantly), CHA2DS2-VASc score (calculated as congestive heart failure, hypertension, age ≥75 years, diabetes, stroke or TIA, vascular disease, age 65-74 years, and sex category)	Age, race/ethnicity, comorbidities, insurance, income

DRG = Diagnosis-Related Group; iASD = iatrogenic atrial septal defect; INR = international normalized ratio; LAAO = left atrial appendage occlusion; NCHS = National Center for Health Statistics; NSAIDs = nonsteroidal anti-inflammatory drugs; other abbreviations as in Tables 1 and 2.

### Procedural success and characteristics

#### Procedural success

**Figure 3:**
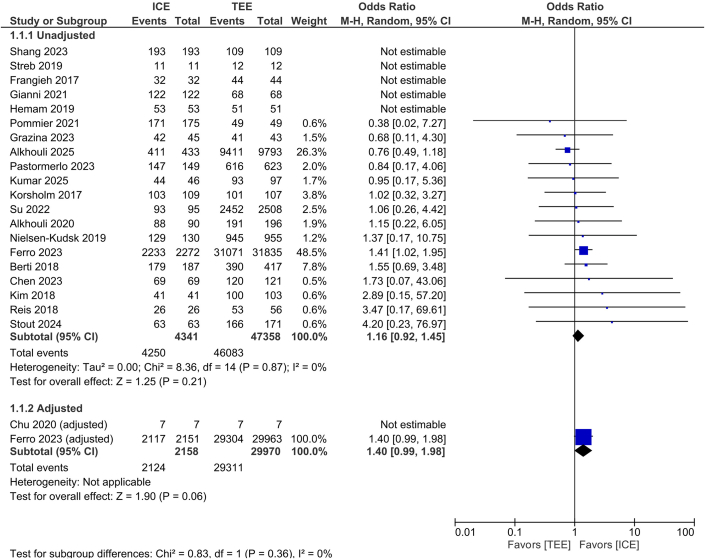
Procedural Success Forest plot of procedural success for intracardiac echocardiography vs transesophageal echocardiography. The analysis is stratified by unadjusted and adjusted subgroups. ORs with 95% CIs are shown, with each square representing study weight. TEE = transesophageal echocardiography; other abbreviation as in Figure 1.

Procedural success was comparable between ICE and TEE in both unadjusted (OR: 1.16; 95% CI: 0.92-1.45, 95% PI: 1.00-1.01, *P* = 0.21, I^2^ = 0%) and adjusted analyses (OR: 1.40; 95% CI: 0.99-1.98, *P* = 0.06, I^2^ = NA), with no significant subgroup interaction (*P* = 0.36) (Figure 3).

#### Procedural time

**Figure 4:**
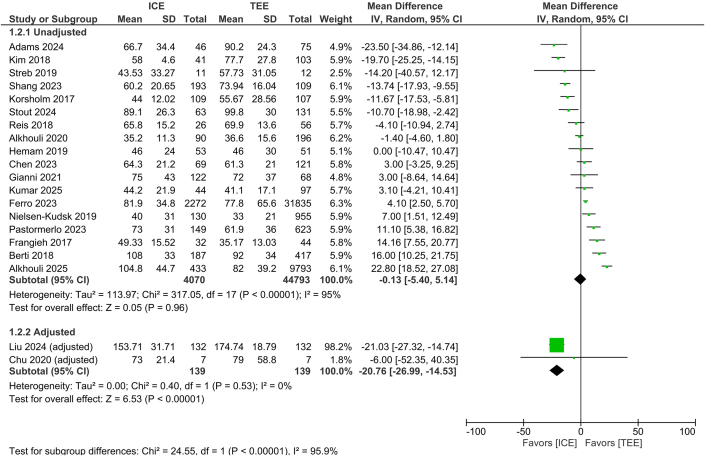
Procedural Time Forest plot of procedural time for intracardiac echocardiography vs transesophageal echocardiography. The analysis is stratified by unadjusted and adjusted subgroups. Mean differences with 95% CIs are shown, with each square representing study weight. Abbreviations as in Figures 1 and 2.

Unadjusted data showed no difference (MD −0.13 minutes; 95% CI: −5.40 to 5.14, 95% PI: −23.35 to 23.10, *P* = 0.96, I^2^ = 95%); however, adjusted analysis demonstrated significantly shorter duration with ICE (MD −20.76 minutes; 95% CI: −26.99 to −14.53, 95% PI: −61.15 to 19.64, *P* < 0.00001, I^2^ = 0%) with a significant subgroup interaction (*P* < 0.00001) (Figure 4).

Regarding secondary procedural metrics, ICE guidance was associated with reduced radiation dose, fewer device attempts, and lower device recapture rates. Additionally, adjusted analyses demonstrated a significant reduction in contrast volume with ICE (Supplemental Figures 2 to 6).

### Complications

#### Pericardial effusion

**Figure 5:**
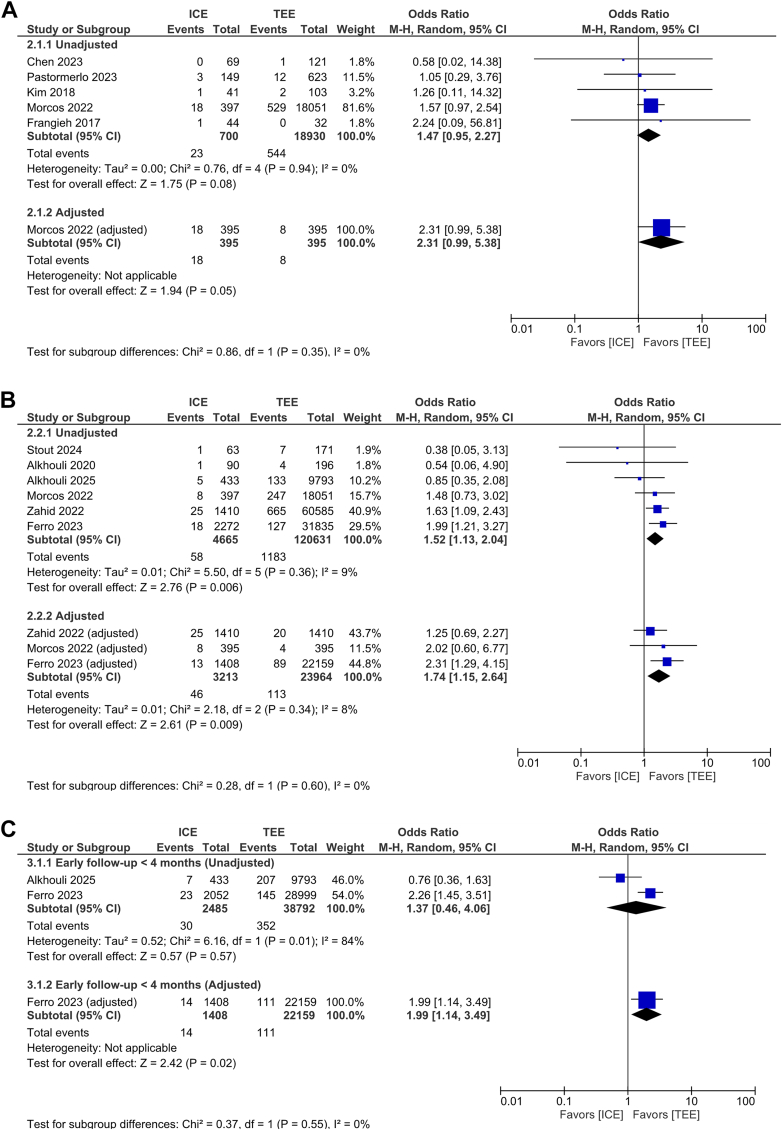
Procedural Effusion Forest plot of procedural effusion outcomes for intracardiac echocardiography vs transesophageal echocardiography. (A) Overall pericardial effusion (in-hospital), (B) Pericardial effusion requiring intervention (in-hospital), (C) Pericardial effusion requiring intervention (follow-up). The analysis is stratified by unadjusted and adjusted subgroups. ORs with 95% CIs are shown, with each square representing study weight. Abbreviations as in Figures 1 and 2.

Regarding in-hospital time point, pericardial effusion (PE) rates were similar between groups in both unadjusted (95% PI: 0.81-2.63, *P* = 0.08, I^2^ = 0%) and adjusted (*P* = 0.05, I^2^ = NA) analyses (Figure 5A). However, ICE was associated with higher odds of PE requiring intervention in both unadjusted (OR: 1.52; 95% CI: 1.13-2.04, 95% PI: 0.93-2.46, *P* = 0.006, I^2^ = 9%) and adjusted (OR: 1.74; 95% CI: 1.15-2.64, 95% PI: 0.60-4.95, *P* = 0.009, I^2^ = 8%) models (Figure 5B).

In the unadjusted analysis of early follow-up (<4 months), there was no statistically significant difference in the risk of PE requiring intervention between ICE and TEE guidance (OR: 1.37; 95% CI: 0.46-4.06, 95% PI: 0.00-126264.77, *P* = 0.57, I^2^ = 84%). In contrast, the adjusted analysis demonstrated a significant higher odds of early PE requiring intervention with ICE guidance compared with TEE (OR: 1.99; 95% CI: 1.14-3.49, *P* = 0.02, I^2^ = NA). There was no significant difference between unadjusted and adjusted subgroup estimates (test for subgroup differences: *P* = 0.55) (Figure 5C).

#### Residual iatrogenic atrial septal defect

**Figure 6:**
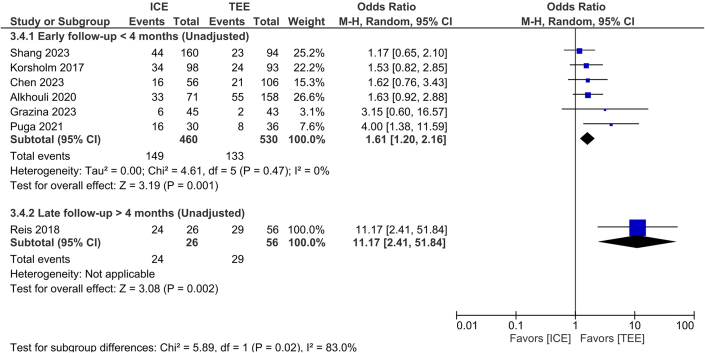
Residual Latrogenic Atrial Septal Defect Forest plot of residual iatrogenic atrial septal defect for intracardiac echocardiography vs transesophageal echocardiography. The analysis is stratified by early> 4 months unadjusted and late <4 months unadjusted subgroups. ORs with 95% CIs are shown, with each square representing study weight. Abbreviations as in Figures 1 and 2.

In the unadjusted early follow-up analysis (<4 months), ICE guidance was associated with a significantly higher incidence of iatrogenic atrial septal defect (iASD) compared with TEE (OR: 1.61; 95% CI: 1.20-2.16, 95% PI: 1.07-1.79, *P* = 0.001, I^2^ = 0%). During late follow-up (>4 months), ICE guidance was associated with a markedly increased persistence of iASD (OR: 11.17; 95% CI: 2.41-51.84, *P* = 0.002, I^2^ = NA). A significant difference between early and late follow-up estimates was observed (test for subgroup differences: *P* = 0.02), with substantial heterogeneity between subgroups (I^2^ = 83%) (Figure 6). Additional safety outcomes are presented in Supplemental Figures 7 to 22.

### Publication bias and sensitivity analysis

Statistical analysis revealed no significant publication bias for the majority of unadjusted outcomes, including procedural success (*P* = 0.86), procedural time (*P* = 0.437), in-hospital overall PE (*P* = 0.339), in-hospital ischemic stroke (*P* = 0.051), early follow-up residual iASD (*P* = 0.066), early follow-up peridevice leak >5 mm (*P* = 0.217), and late follow-up all-cause mortality (*P* = 0.338). However, significant bias was observed for in-hospital PE requiring intervention (*P* = 0.028) and in-hospital all-cause mortality (*P* = 0.006). These analyses are visualized in funnel plots (Supplemental Figures 23 to 31). Furthermore, to evaluate the robustness of pooled outcomes exhibiting significant heterogeneity, a sequential leave-one-out sensitivity analysis was performed. The detailed forest plots are provided in Supplemental Figures 32 to 42.

### Meta-regression

Meta-regression was performed to explore the impact of baseline CHA2DS2-VASc and HAS-BLED scores on the unadjusted pooled outcomes (Supplemental Table 2). For procedural success, the interactive model accounted for all observed heterogeneity (R^2^ = 100%). Although individual CHA2DS2-VASc (estimate: 9.27; *P* < 0.0001) and HAS-BLED (estimate: 16.18; *P* < 0.0001) scores independently predicted the outcome, a significant negative interaction between them (estimate: −3.87; 95% CI: -4.96 to −2.78; *P* < 0.0001) indicated that their concurrent elevation significantly attenuates this effect.

## Discussion

### Summary of key findings

This updated meta-analysis of 27 nonrandomized observational studies, comparing ICE vs TEE guidance for LAAO, demonstrated the following key findings: 1) ICE guidance demonstrated comparable procedural success rates to TEE; 2) in the adjusted analyses, ICE was associated with significantly shorter procedural times; 3) ICE was associated with a higher risk of severe PE requiring intervention and residual iASD. However, there were no significant differences in all-cause mortality, ischemic stroke, or significant peridevice leak (>5 mm) (Central Illustration).

**Central Illustration:**
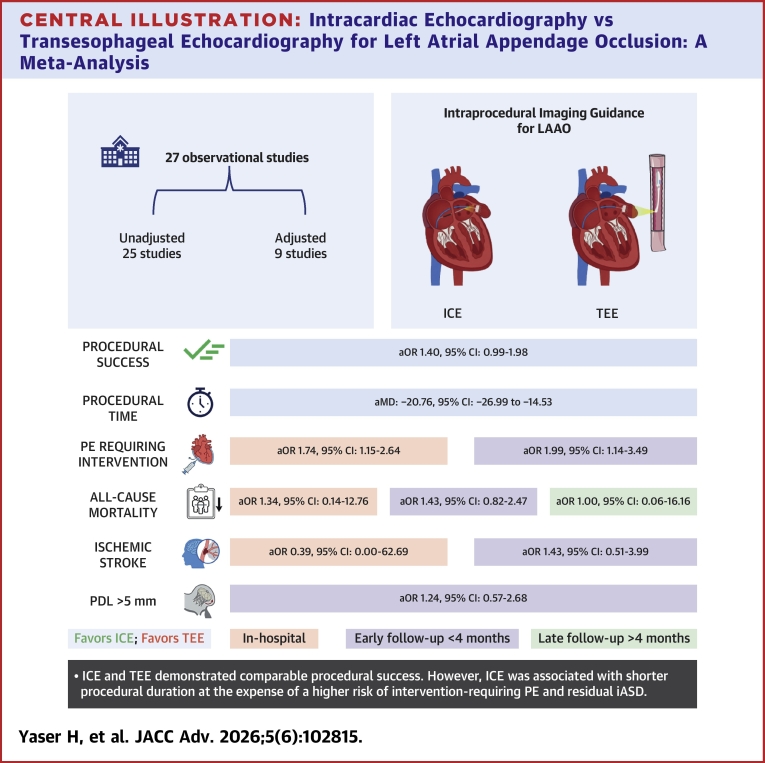
Intracardiac Echocardiography vs Transesophageal Echocardiography for Left Atrial Appendage Occlusion: A Meta-Analysis In this meta-analysis of 27 observational studies, intracardiac echocardiography and transesophageal echocardiography demonstrated comparable procedural success for intraprocedural guidance of left atrial appendage occlusion. However, intracardiac echocardiography was associated with shorter procedural duration at the expense of a higher risk of intervention-requiring pericardial effusion and residual iatrogenic atrial septal defect. aMD = adjusted mean difference; aOR = adjusted OR; iASD = iatrogenic atrial septal defect; LAAO = left atrial appendage occlusion; PDL = peridevice leak; PE = pericardial effusion; TEE = transesophageal echocardiography; other abbreviations as in Figures 1 and 2.

### Procedural success

Our pooled analysis found no significant difference in procedural success rates between ICE and TEE. Although not directly assessed in our quantitative model, data from the included studies suggest that a potential determinant of this might be related to the operator learning curve rather than the modality alone. Unlike TEE, ICE adoption is still growing, and many operators are in the early phase of the learning curve.^21^^,^^22^ Indeed, Alkhouli et al (2025) demonstrated that operators with high ICE experience (≥30 cases) achieved significantly higher success rates compared to those with early experience (<10 cases).^21^ This reliance on operator proficiency is widely acknowledged and is now reflected in the design of upcoming randomized trials, which explicitly mandates a minimum of 3 successful ICE-guided cases prior to operator participation. Furthermore, the impact of confounding variables remains complex.^48^ For instance, Ferro et al (2023) found that the statistical advantage of ICE observed in unadjusted analysis was attenuated to nonsignificance after adjustment.^22^ This aligns with Su et al (2022), where analyses demonstrated no significant difference in procedural success in either unadjusted or adjusted models.^23^ This consistency across high-quality adjusted data sets suggests that observed outcomes may be driven by patient selection and adjustment factors, including operator expertise and risk scores, implying that procedural success is multifactorial rather than dependent on the imaging modality itself.

### Procedural time

Regarding procedural time, we found no significant differences in the unadjusted analysis, a finding likely attributable to the significant heterogeneity observed within that subgroup. However, our adjusted analysis revealed a significant reduction in procedural time with ICE. These results were primarily driven by Liu et al (2024), which included procedures performed by a single experienced operator (which minimizes operator variability despite limiting generalizability), mandated the use of both cardiac computed tomography and TEE for preprocedural planning. The critical role of operator proficiency was further confirmed by Ferro et al (2023) and Alkhouli et al (2025), who demonstrated that operators with high ICE case volumes achieved significantly shorter procedure times compared to those in the early phases of the learning curve.^21^^,^^22^ The advantage of concomitant procedures to ICE was similarly observed by Shang et al (2023), suggesting that TEE may be a less optimal intraprocedural option when LAAO is performed concomitantly with ablation. Beyond workflow, specific techniques and technological advancements also appear to drive these results.^45^ Kim et al (2018) achieved shorter times by positioning the ICE probe at the left superior pulmonary vein, mirroring the XR-Star workflow.^34^^,^^38^ Furthermore, while most studies relied on 2-dimensional (2D) ICE, Adams et al (2024) utilized 4-dimensional (4D) ICE in the comparison with intraprocedural TEE. This study achieved shorter procedural times, highlighting the gains from reduced catheter manipulation and comprehensive visualization.^25^^,^^49^

### Safety outcomes

Regarding safety outcomes, our analysis revealed a consistent signal across both unadjusted and adjusted subgroups. While ICE was comparable to TEE for overall PE and cardiac tamponade, it was associated with a significantly higher risk of PE requiring intervention. This could be related to the transseptal advancement and manipulation of a rigid ultrasound catheter within the left atrium.^50^^,^^51^ Furthermore, patient selection may confound this risk; a previous large-scale analysis identified advanced age and higher CHA2DS2-VASc scores as independent predictors of intervention-requiring PE, suggesting that the preferential use of ICE in frailer patients may contribute to the observed safety signal.^52^ Despite the low absolute incidence, its significant association with mortality underscores a critical need for risk mitigation.^51^^,^^52^ Encouragingly, this risk appears to be modifiable by experience; both Ferro et al (2023) and Alkhouli et al (2025) observed a reduction in intervention-requiring PE rates as operator volume increased, though this trend did not reach statistical significance in the latter analysis, highlighting the potential impact of the learning curve.^21^^,^^22^

Similarly, we observed that ICE guidance was consistently associated with a higher rate of residual iASD. Unlike previous meta-analyses,^13^^,^^14^ we explicitly stratified outcomes into early and late follow-up subgroups, confirming that the significantly higher risk associated with ICE persists across both time points. While exclusive ICE usage was identified as the primary driver for iASD incidence, independent of device size or mitral regurgitation, defect persistence was found to be correlated with patient-specific factors including device size, significant mitral regurgitation, and CHA_2_DS_2_-VASc scores.^43^^,^^53^ Importantly, this persistence appears clinically benign, as nearly half of these defects close spontaneously within a year—with persistence rates declining from 66.0% at 6 months to 52.0% at 12 months post-LAAO.^53^ Furthermore, existing literature demonstrates that even when persistent, iASDs have no significant impact on long-term rates of stroke, systemic thromboembolism, or all-cause mortality.

### Strengths

Our study has several key strengths that distinguish it from prior meta-analyses.^12^, ^13^, ^14^ First, it represents the largest meta-analysis to date, incorporating a significantly larger sample size than previous reviews, which enhances the statistical power of our safety signals. Second, we employed a rigorous methodological approach by explicitly separating adjusted observational data from unadjusted cohorts. This stratification allowed us to better account for baseline confounding variables, offering a more refined assessment of the outcomes. Third, our analysis provides a granular assessment of safety outcomes by evaluating complications at 3 distinct time points: in-hospital, early follow-up (<4 months), and late follow-up (>4 months). This temporal stratification helped identify the evolution of specific risks, such as the persistence of iASDs, which although appearing clinically benign, might otherwise be obscured in a pooled aggregate analysis.

### Study limitations

These findings must be interpreted in the context of certain limitations. First, despite the large overall sample size, the availability of adjusted data was scarce across multiple endpoints (only 9 of 27 included studies). Consequently, most of our pooled outcomes remain driven by observational and potentially confounded unadjusted data. While we explicitly stratified our analyses to report adjusted estimates where available, the limited number of such studies reduces the robustness of causal inference. Furthermore, the specific confounding factors used for adjustment varied significantly across these studies. This lack of standardization restricts the robustness of these specific findings compared to the broader unadjusted analysis. Second, there was notable heterogeneity in the definition of “procedural success” across the included studies, ranging from successful deployment to complete seal without leak, which introduces variability in the efficacy endpoint. Furthermore, the statistical power of meta-regression analyses may be limited for certain outcomes, as these tests optimally require a minimum of 10 included studies.^54^^,^^55^ Finally, nearly all included studies utilized 2D ICE technology, with only 1 study (Adams et al) utilizing 4D ICE.^25^ As 4D ICE offers superior spatial resolution the findings of this meta-analysis may primarily reflect the capabilities of the older 2D generation.

## Conclusions

This meta-analysis suggests that ICE offers comparable procedural success and potentially shorter times, compared with TEE for LAAO guidance. However, ICE is associated with a higher risk of intervention-requiring PE and residual iASD. These results suggest that while ICE is a promising alternative, rigorous operator training and future randomized studies are essential to confirm these findings.

Perspectives **COMPETENCY IN MEDICAL KNOWLEDGE:** For LAAO guidance, ICE offers comparable procedural success and shorter procedure times than TEE. However, operators must recognize an increased incidence of mechanical trauma with ICE, specifically intervention-requiring PE and residual iASD. To optimize safety, clinicians should ensure rigorous training, and carefully weigh these risks during patient selection.

**TRANSLATIONAL OUTLOOK:** While observational data establish ICE as a promising alternative to TEE for LAAO, current evidence relies predominantly on older 2D ICE technology. Future adequately powered, multicenter randomized controlled trials are essential. Specifically, these trials must evaluate whether emerging 4D volumetric ICE technologies, combined with standardized operator training, can mitigate the risks of ICE.

## Funding support and author disclosures

The authors have reported that they have no relationships relevant to the contents of this paper to disclose.
