# Supplementary material for: Intracardiac Versus Transesophageal Echocardiography for Left Atrial Appendage Occlusion: A Meta-Analysis
Source: JACC Adv. 2026 May 20;5(6):102815. doi: 10.1016/j.jacadv.2026.102815 (PMC13217600; doi:10.1016/j.jacadv.2026.102815)
Supplement: Supplemental_Material [file mmc1.docx]

Table of Contents

**Supplemental Table 1**, Search strategy utilized in the databases…………………………..….…. **3
Supplemental Table 2**, Meta-regression of unadjusted pooled outcomes (additive and interactive models)……………………………………………………………………………………………. **4**
**Supplemental Figure 1**, ROBINS-I weighted summary plot………………………………….…. **5**
**Supplemental Figure 2**, Forest plot of mean difference in Fluoroscopy time for ICE vs. TEE… **6**
**Supplemental Figure 3**, Forest plot of mean difference in Contrast volume for ICE vs. TEE. …….**6**
**Supplemental Figure 4**, Forest plot of mean difference for ICE vs TEE, A; Radiation dose, B; Dose area product………………………………………………………………..………………..**7**
**Supplemental Figure 5,** Forest plot of mean difference for ICE vs. TEE, A; Device attempted, B; Device recapture, C; Device used. …………………………………………………...…………….**8**
**Supplemental Figure 6**, Forest plot of mean difference for ICE vs. TEE, A; Length of hospital stay, B; Cost of hospitalization……………………………………...……………………………...**9**
**Supplemental Figure 7**, Forest plot of odds ratios for ICE vs. TEE, A; Pericardial effusion requiring pericardiocentesis (In-hospital complications), B; Pericardial effusion requiring surgery(In-hospital complications), C; Cardiac tamponade(In-hospital complications), D; Pericardial effusion requiring pericardiocentesis( Follow-up complications), E; Pericardial effusion requiring surgery( Follow-up complications)……………...………………………..…………… **10**
**Supplemental Figure 8**, Forest plot of mean difference in ASD size (Follow-up complications) for ICE vs TEE……………………………………………………………………...………………...**11**
**Supplemental Figure 9**, Forest plot of odds ratios for ICE vs. TEE, A; peridevice leak (In-hospital complications), B; peridevice leak (Follow-up complications)…………………………………...**12**
**Supplemental Figure 10**, Forest plot of odds ratios for ICE vs. TEE, A; peridevice leak >5mm (In-hospital complications), B; peridevice leak>5mm (Follow-up complications)……………..…….**13**
**Supplemental Figure 11**, Forest plot of odds ratios for ICE vs. TEE, A; peridevice leak<5mm (In-hospital complications), B; peridevice leak<5mm (Follow-up complications)………………...…**14**
**Supplemental Figure 12**, Forest plot of odds ratios for ICE vs. TEE, A; Device related thrombus (In-hospital complications), B; Device related thrombus (Follow-up complications)…………….**15**
**Supplemental Figure 13**, Forest plot of odds ratios for ICE vs. TEE, A; All-cause mortality (In-hospital complications), B; All-cause mortality (Follow-up complications)……………..…….…**16**
**Supplemental Figure 14**, Forest plot of odds ratios for ICE vs. TEE, A; Stroke (In-hospital complications), B; Stroke (Follow-up complications)…………………………………………….**17**
**Supplemental Figure 15**, Forest plot of odds ratios for ICE vs. TEE, A; Ischemic stroke (In-hospital complications), B; Ischemic stroke (Follow-up complications)………………………….**18**
**Supplemental Figure 16**, Forest plot of odds ratios for ICE vs. TEE, A; Hemorrhagic stroke (In-hospital complications), B; Hemorrhagic stroke (Follow-up complications)…………………..…**19**
**Supplemental Figure 17**, Forest plot of odds ratios for ICE vs. TEE, A; Transient Ischemic Attack (In-hospital complications), B; Transient Ischemic Attack (Follow-up complications)…………...**20**
**Supplemental Figure 18**, Forest plot of odds ratios for ICE vs. TEE, A; Major bleeding (In-hospital complications), B; Major bleeding (Follow-up complications)……………………….….**21**
**Supplemental Figure 19**, Forest plot of odds ratios for ICE vs. TEE, A; Device embolization (In-hospital complications), B; Device embolization (Follow-up complications)…………………….**22**
**Supplemental Figure 20**, Forest plot of odds ratios for ICE vs. TEE, A; Myocardial infarction (In-hospital complications), B; Cardiac arrest (In-hospital complications)…………………………...**23**
**Supplemental Figure 21**, Forest plot of odds ratios for ICE vs. TEE, A; Vascular complications (In-hospital complications), B, Vascular complications (Follow-up complications)……………...**24**
**Supplemental Figure 22**, Forest plot of odds ratios of Pseudoaneurysm (In-hospital complications) for ICE vs. TEE………………………………………………………………………..………….**25
Supplemental Figure 23**, Funnel plot of procedural success....…………………………………..**25
Supplemental Figure 24**, Funnel plot of procedural time…….…………………………………..**26
Supplemental Figure 25**, Funnel plot of in-hospital overall PE………………………………….**26
Supplemental Figure 26**, Funnel plot of PE requiring intervention………………………….......**27
Supplemental Figure 27**, Funnel plot of in-hospital all-cause mortality………………………....**27
Supplemental Figure 28**, Funnel plot of in-hospital ischemic stroke....………………...……..…**28
Supplemental Figure 29**, Funnel plot of early follow-up residual iASD...…………………..…...**28
Supplemental Figure 30**, Funnel plot of early follow-up PDL > 5 mm.…………………..……...**29
Supplemental Figure 31**, Funnel plot of late follow-up all-cause mortality..………………..…...**29
Supplemental Figure 32**, Leave-one-out sensitivity analysis of unadjusted procedural time…....**30
Supplemental Figure 33**, Leave-one-out sensitivity analysis of unadjusted fluoroscopy time…..**30
Supplemental Figure 34**, Leave-one-out sensitivity analysis of unadjusted contrast volume…....**31
Supplemental Figure 35**, Leave-one-out sensitivity analysis of unadjusted radiation dose...…....**31
Supplemental Figure 36**, Leave-one-out sensitivity analysis of unadjusted device attempted......**32
Supplemental Figure 37**, Leave-one-out sensitivity analysis of unadjusted device recapture.......**32
Supplemental Figure 38**, Leave-one-out sensitivity analysis of unadjusted length of hospital stay………………………………………………………………………………………………..**33
Supplemental Figure 39**, Leave-one-out sensitivity analysis of unadjusted PE requiring pericardiocentesis…………………………………………………………………………………**33
Supplemental Figure 40**, Leave-one-out sensitivity analysis of unadjusted stroke………………**34
Supplemental Figure 41**, Leave-one-out sensitivity analysis of unadjusted ischemic stroke…..**34
Supplemental Figure 42**, Leave-one-out sensitivity analysis of unadjusted cardiac arrest……....**35**

| base (Number of studies) | Search strategy |
| --- | --- |
| PubMed (100) | "atrial fibrillation"[MeSH Terms] AND ("atrial appendage"[MeSH Terms] OR "atrial append*"[Title/Abstract] OR LAA[Title/Abstract]) AND (occlusion[Title/Abstract] OR occlud*[Title/Abstract] OR closure[Title/Abstract] OR exclusion[Title/Abstract] OR LAAC[Title/Abstract] OR LAAO[Title/Abstract]) AND ("transesophageal echocardiograph*" OR TEE) AND ("intracardiac echocardiograph*" OR ICE) |
| Web of Science (89) | ALL=("atrial fibrillation" AND ("atrial append*" OR LAA) AND (occlusion OR occlud* OR closure OR exclusion OR LAAC OR LAAO) AND ("transesophageal echocardiograph*" OR TEE) AND ("intracardiac echocardiograph*" OR ICE)) |
| Cochrane library (10) | ("atrial fibrillation") AND ("atrial appendage" OR "atrial NEXT append*" OR "LAA"):ti,ab,kw AND (occlusion OR "NEXT occlud*" OR occluded OR closure OR exclusion OR LAAC OR LAAO):ti,ab,kw AND ("transesophageal" OR TEE):ti,ab,kw AND ("intracardiac" OR ICE):ti,ab,kw (Word variations have been searched) |
| Scopus (309) | ALL ( atrial fibrillation ) AND TITLE-ABS-KEY ( ( "atrial appendage" OR "atrial append*" OR LAA ) AND ( occlusion OR occlud* OR closure OR exclusion OR LAAC OR LAAO ) AND ( "transesophageal echocardiograph*" OR TEE ) AND ( "intracardiac echocardiograph*" OR ICE ) ) |

Supplemental Table 1, Search strategy utilized in the databases.

| Additive model | Estimate (95% CI) | P value | Interactive model | Estimate (95% CI) | P value |
| --- | --- | --- | --- | --- | --- |
| Procedural success (k = 18 / R^2^ = 6.12%) | | | Procedural success (k = 18 / R^2^ = 100%) | | |
| CHA2DS2VASc | -0.23 (-1.01; 0.55) | 0.538 | CHA2DS2VASc | 9.27 (6.10; 12.44) | <.0001 |
| HAS-BLED | -0.16 (-0.59; 0.27) | 0.436 | HAS-BLED | 16.18 (11.21; 21.15) | <.0001 |
|  |  |  | CHA2DS2VASc: HAS-BLED | -3.87 (-4.96; -2.78) | <.0001 |
| Procedural time (k = 16 / R^2^ = 100%) | | | Procedural time (k = 16 / R^2^ = 100%) | | |
| CHA2DS2VASc | 11.73 (-2.19; 25.65) | 0.092 | CHA2DS2VASc | 62.27 (-27.47; 152.01) | 0.157 |
| HAS-BLED | 2.40 (-8.74; 13.54) | 0.649 | HAS-BLED | 93.57 (-66.77; 253.91) | 0.228 |
|  |  |  | CHA2DS2VASc: HAS-BLED | -21.12 (-58.19; 15.94) | 0.238 |
| In-hospital overall PE (k = 4 / R^2^ = 0%) | | |  | | |
| CHA2DS2VASc | -13.80 (-119.68; 92.08) | 0.346 |  |  |  |
| HAS-BLED | -4.20 (-37.23; 28.84) | 0.353 |  |  |  |
| In-hospital all-cause mortality (k = 4 / R^2^ = 0%) | | |  |  |  |
| CHA2DS2VASc | -4.23 (-72.78; 64.33) | 0.577 |  |  |  |
| HAS-BLED | -4.78 (-43.82; 34.27) | 0.364 |  |  |  |
| In-hospital ischemic stroke (k = 4 / R^2^ = 0%) | | |  |  |  |
| CHA2DS2VASc | 4.52 (-145.70; 154.74) | 0.768 |  |  |  |
| HAS-BLED | 1.29 (-56.51; 59.09) | 0.824 |  |  |  |
| Early follow-up residual iASD (k = 6 / R^2^ = 3.08%) | | | Early follow-up residual iASD (k = 6 / R^2^ = 100%) | | |
| CHA2DS2VASc | -0.34 (-1.43; 0.76) | 0.400 | CHA2DS2VASc | 7.70 (-8.06; 23.46) | 0.170 |
| HAS-BLED | 0.15 (-0.71; 1.01) | 0.620 | HAS-BLED | 12.16 (-12.65; 36.98) | 0.169 |
|  |  |  | CHA2DS2VASc: HAS-BLED | -2.92 (-8.94; 3.09) | 0.171 |
| Early follow-up PDL > 5 mm (k = 5 / R^2^ = 0%) | | | Early follow-up PDL > 5 mm (k = 5 / R^2^ = 0%) | | |
| CHA2DS2VASc | -2.41 (-13.71; 8.89) | 0.456 | CHA2DS2VASc | 10.04 (-76.81; 96.89) | 0.380 |
| HAS-BLED | -1.05 (-4.79; 2.68) | 0.350 | HAS-BLED | 18.28 (-112.26; 148.81) | 0.326 |
|  |  |  | CHA2DS2VASc: HAS-BLED | -4.38 (-33.90; 25.14) | 0.311 |
| In-hospital ischemic stroke (k = 4 / R^2^ = 0%) | | |  |  |  |
| CHA2DS2VASc | 0.66 (-5.24; 6.57) | 0.389 |  |  |  |
| HAS-BLED | 0.23 (-8.62; 9.09) | 0.794 |  |  |  |

Supplemental Table 2, Meta-regression of unadjusted pooled outcomes (additive and interactive models).
k : number of studies, R^2^ : Amount of heterogeneity accounted for.


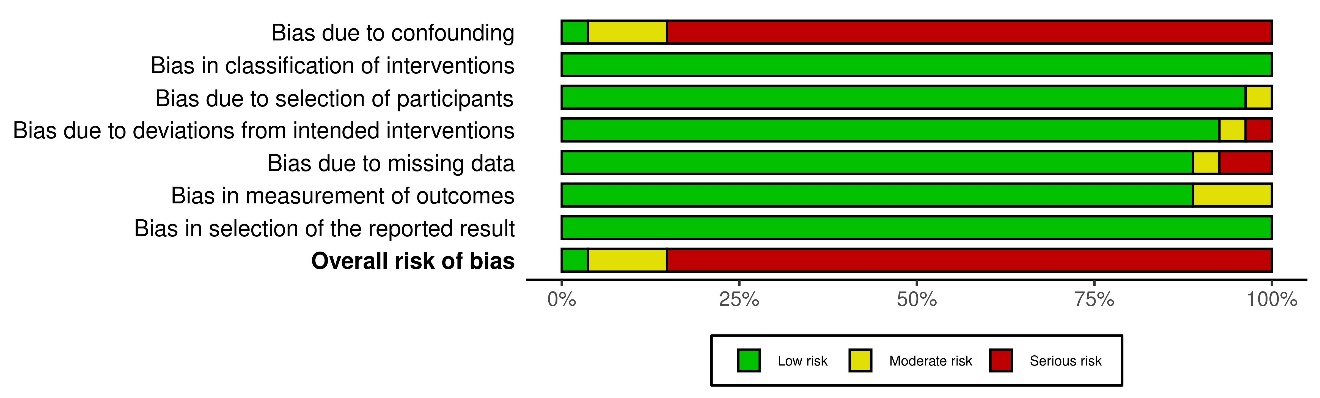


Supplemental Figure 1, ROBINS-I weighted summary plot.


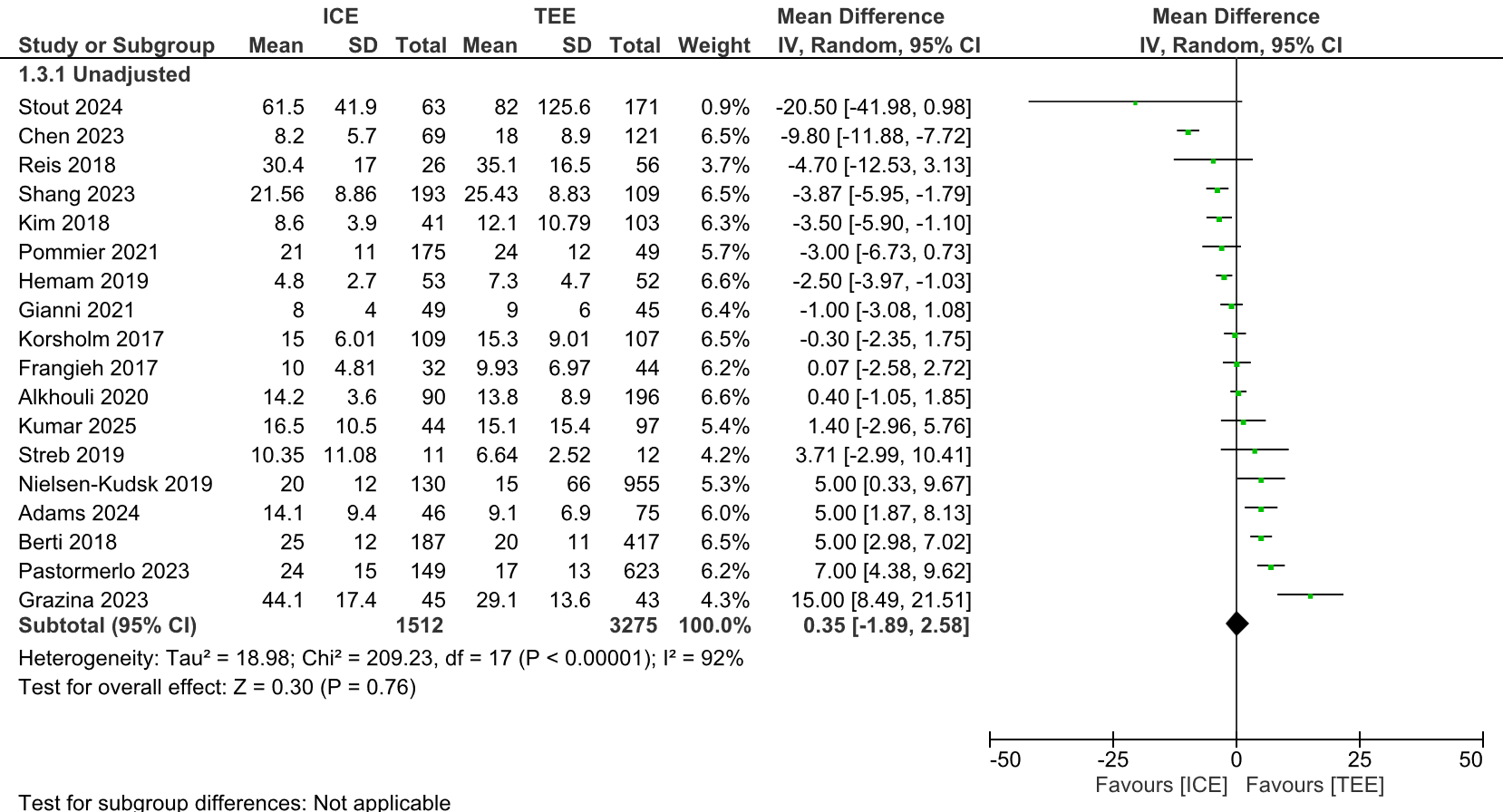


Supplemental Figure 2, Forest plot of mean difference in Fluoroscopy time for ICE vs. TEE. 95% PI [-9.15; 9.85].


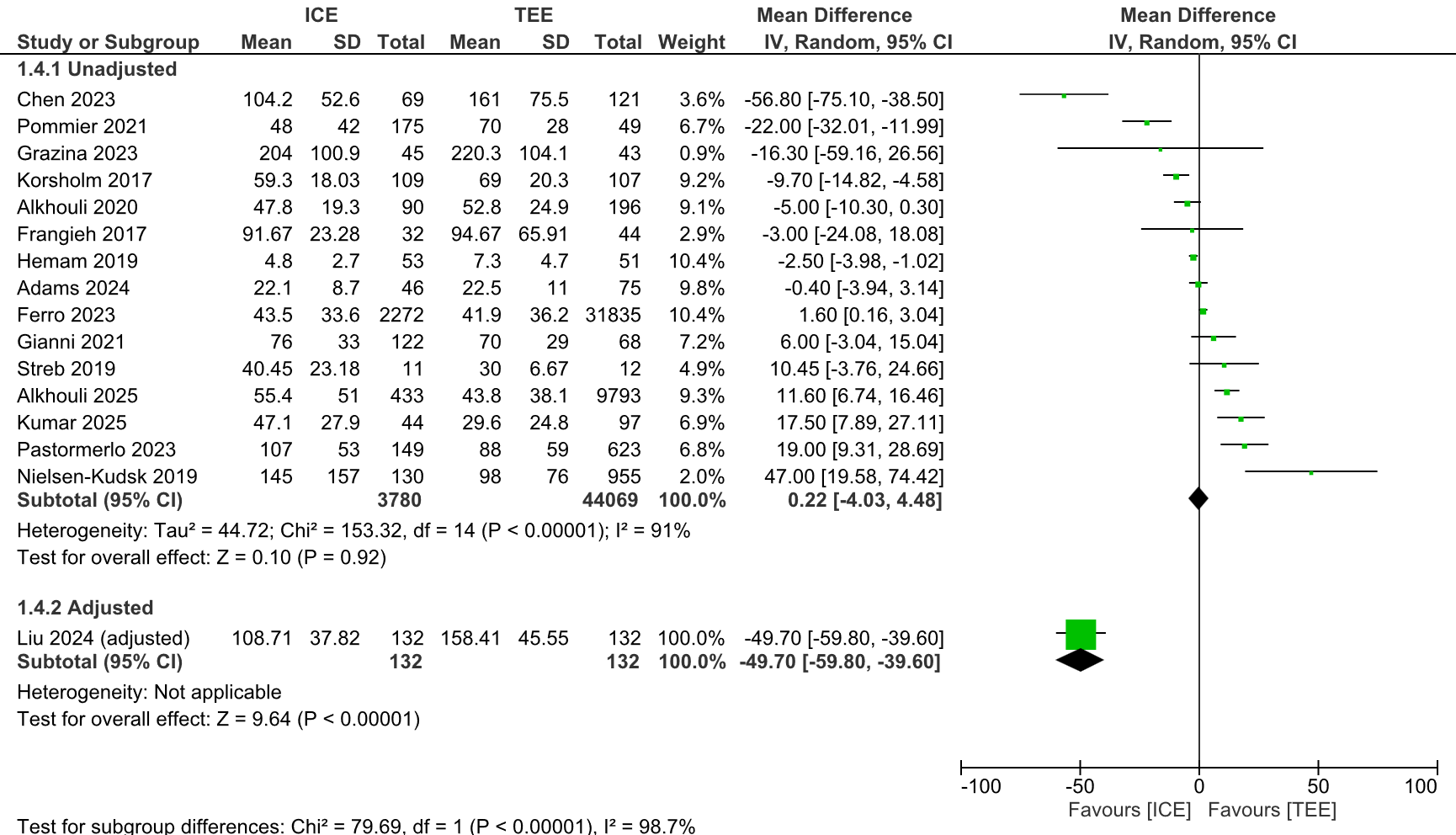


Supplemental Figure 3,Forest plot of mean difference in Contrast volume for ICE vs. TEE. Unadjusted 95% PI [-14.86; 15.31].


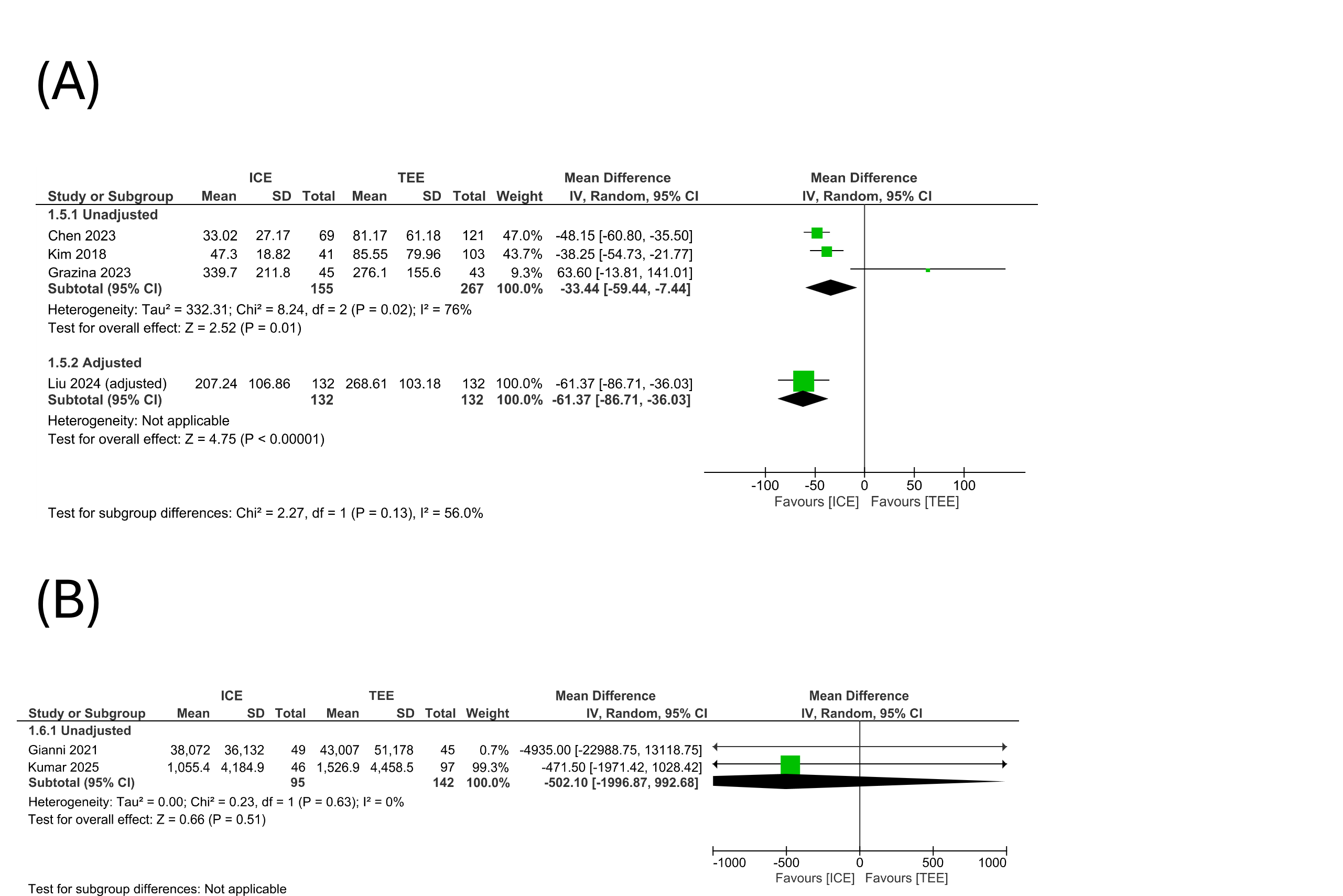


Supplemental Figure 4, Forest plot of mean difference for ICE vs. TEE, A; Radiation dose, unadjusted 95% PI [-130.44; 63.56], B; Dose area product, unadjusted 95% PI [-10192.54; 9188.34].


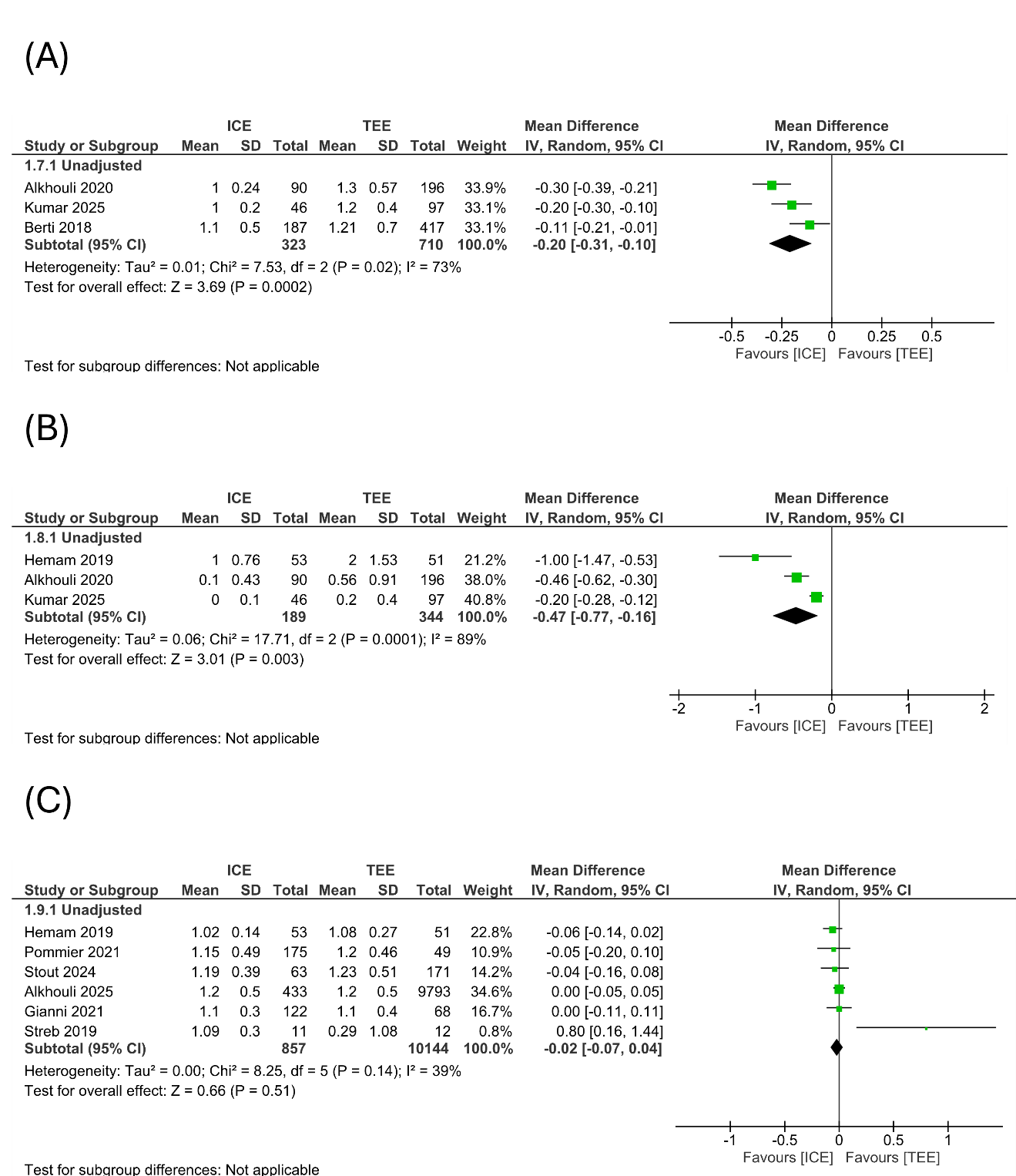


Supplemental Figure 5,Forest plot of mean difference for ICE vs. TEE, A; Device attempted, unadjusted 95% PI [-0.63; 0.22], B; Device recapture, unadjusted PI= [-1.70; 0.76], C; Device used, unadjusted 95% PI [-0.15; 0.11].


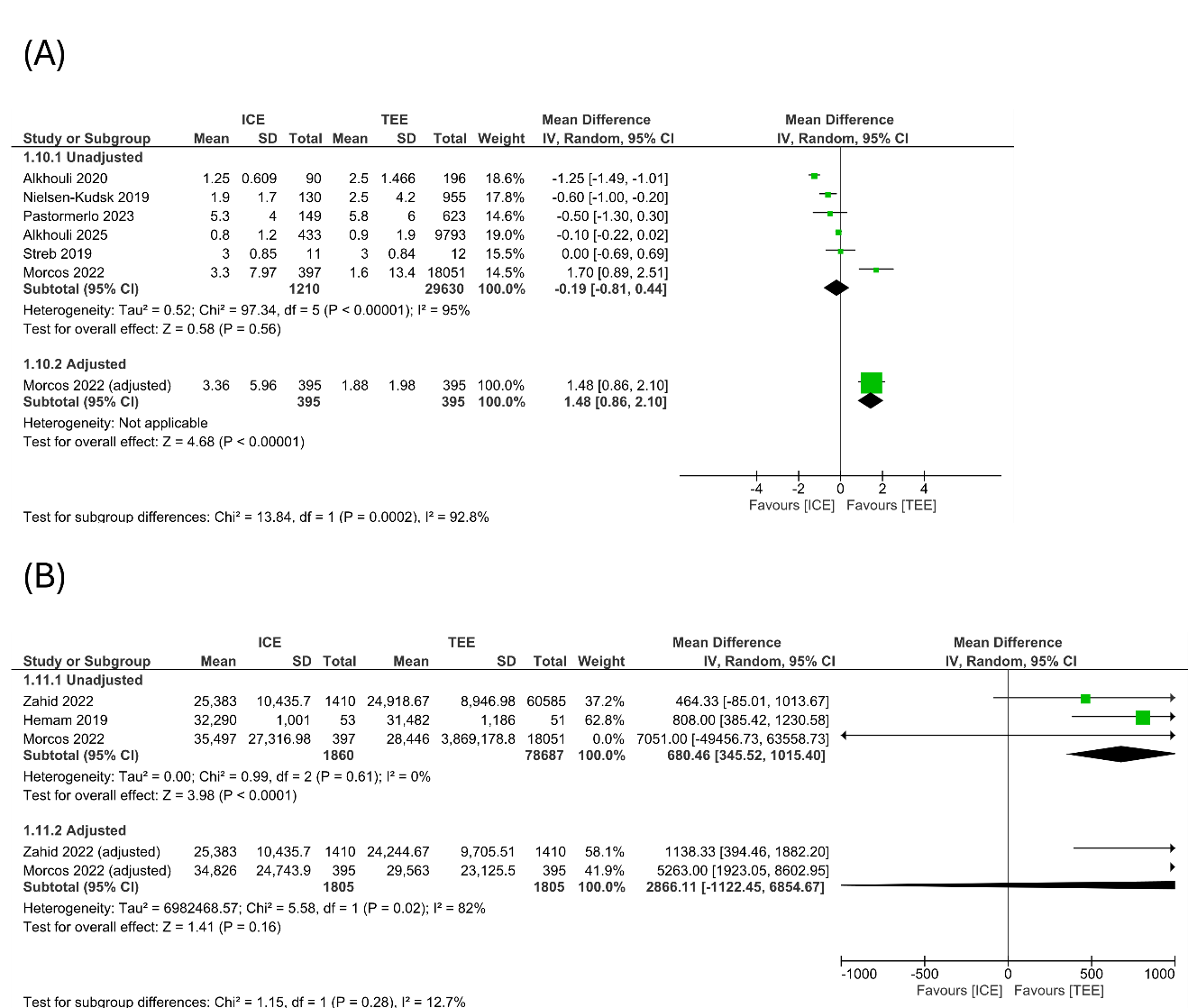


Supplemental Figure 6, Forest plot of mean difference for ICE vs. TEE, A; Length of hospital stay, unadjusted 95% PI [-2.22; 1.85], B; Cost of hospitalization, unadjusted PI= [-54.82; 1415.74], adjusted 95% PI [-39512.02; 45244.23].


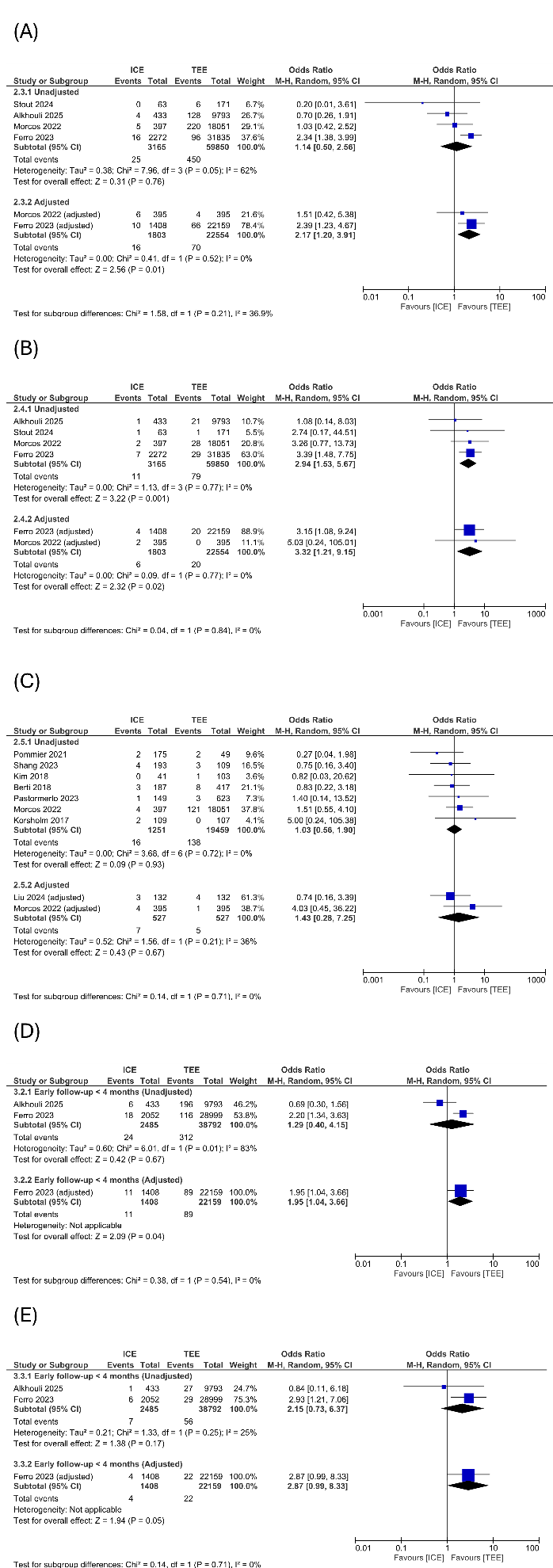


Supplemental Figure 7,Forest plot of odds ratios for ICE vs. TEE, A; Pericardial effusion requiring pericardiocentesis (In-hospital complications), unadjusted 95% PI [0.11; 11.94], adjusted 95% PI [0.05; 96.53], B; Pericardial effusion requiring surgery (In-hospital complications), unadjusted 95% PI [1.01; 8.48], adjusted 95% PI [0.01; 2324.78], C; Cardiac tamponade (In-hospital complications), unadjusted 95% PI [0.48; 2.18], adjusted 95% PI [0.00; 1480761.16], D; Pericardial effusion requiring pericardiocentesis (Follow-up complications), unadjusted 95% PI [0.00; 282693.97], E; Pericardial effusion requiring surgery (Follow-up complications), unadjusted 95% PI [0.01; 19066.40].


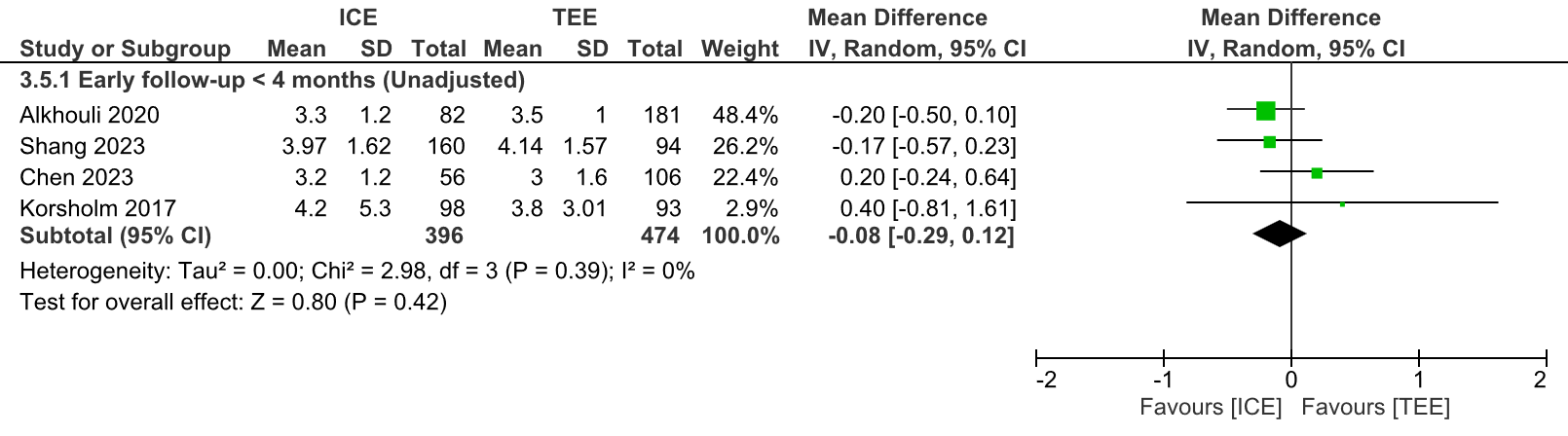


Supplemental Figure 8, Forest plot of mean difference in ASD size (Follow-up complications) for ICE vs TEE, unadjusted 95% PI [-0.42; 0.25].


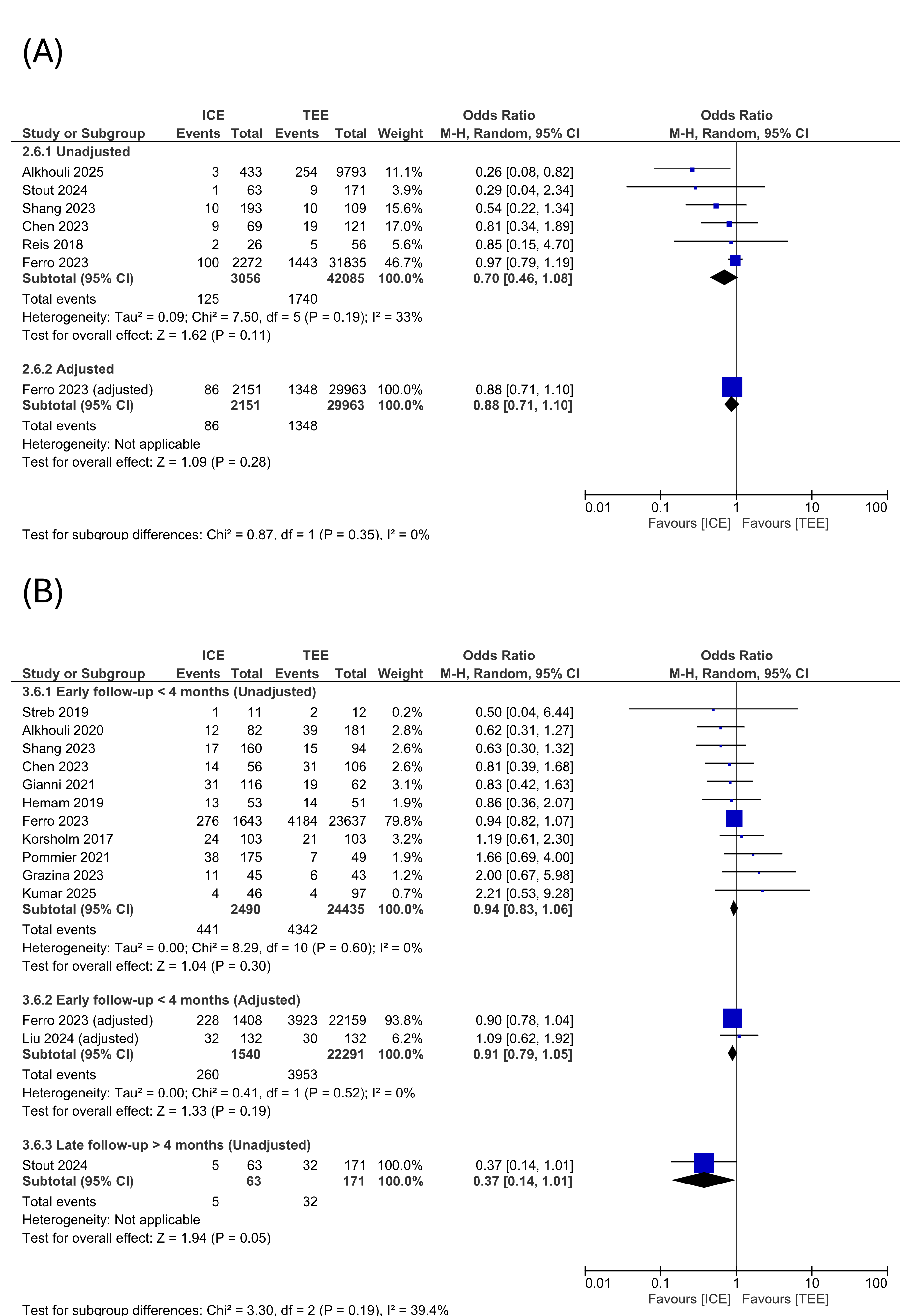


Supplemental Figure 9, Forest plot of odds ratios for ICE vs. TEE, A; peridevice leak (In-hospital complications), unadjusted 95% PI [0.30; 1.75], B; peridevice leak (Follow-up complications), early unadjusted 95% PI [0.85; 1.06], early adjusted 95% PI [0.43; 1.98].


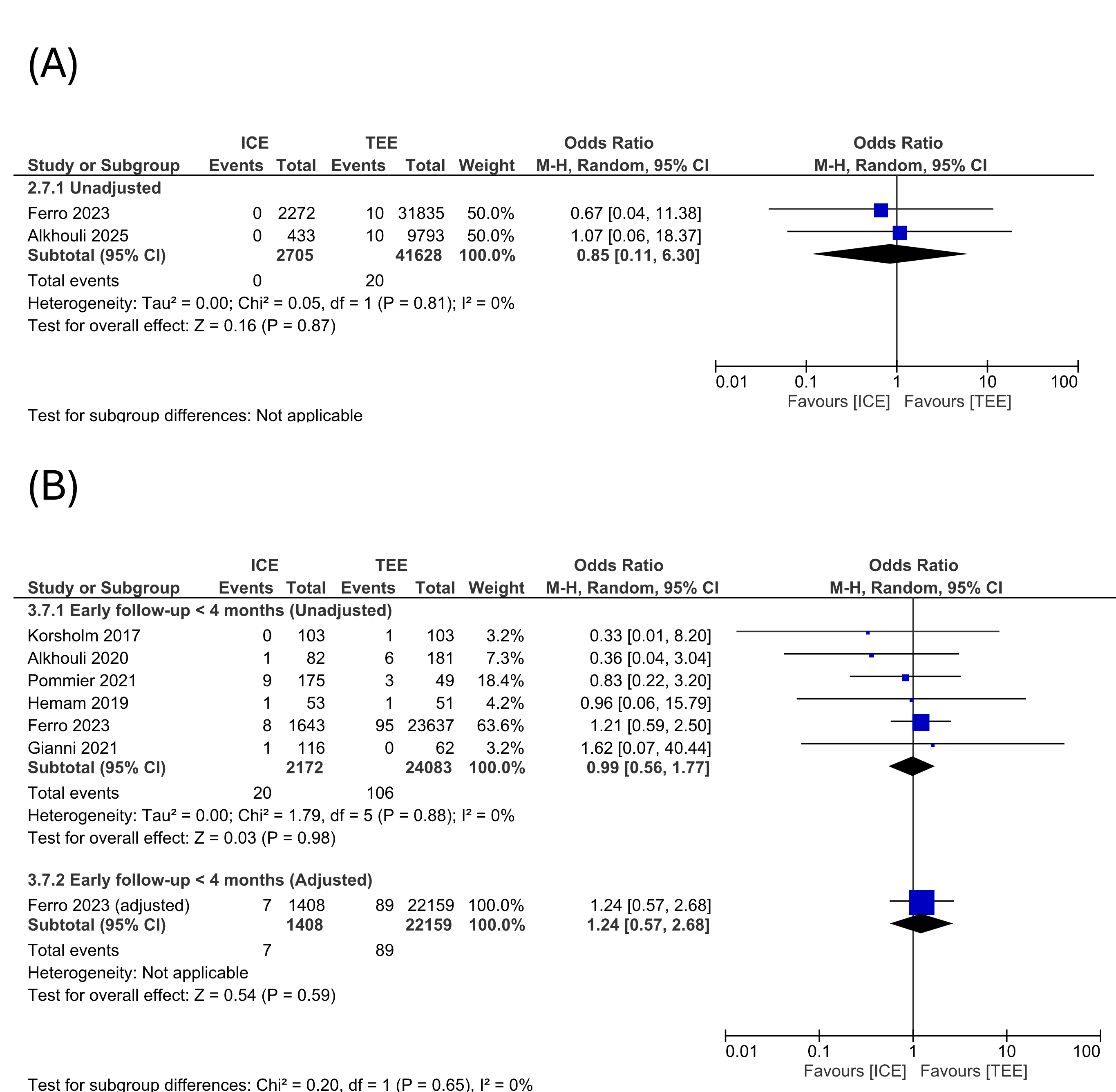


Supplemental Figure 10,Forest plot of odds ratios for ICE vs. TEE, A; peridevice leak >5mm (In-hospital complications), unadjusted 95% PI [0.00; 375109.56], B; peridevice leak>5mm (Follow-up complications), early unadjusted 95% PI [0.47; 2.08].


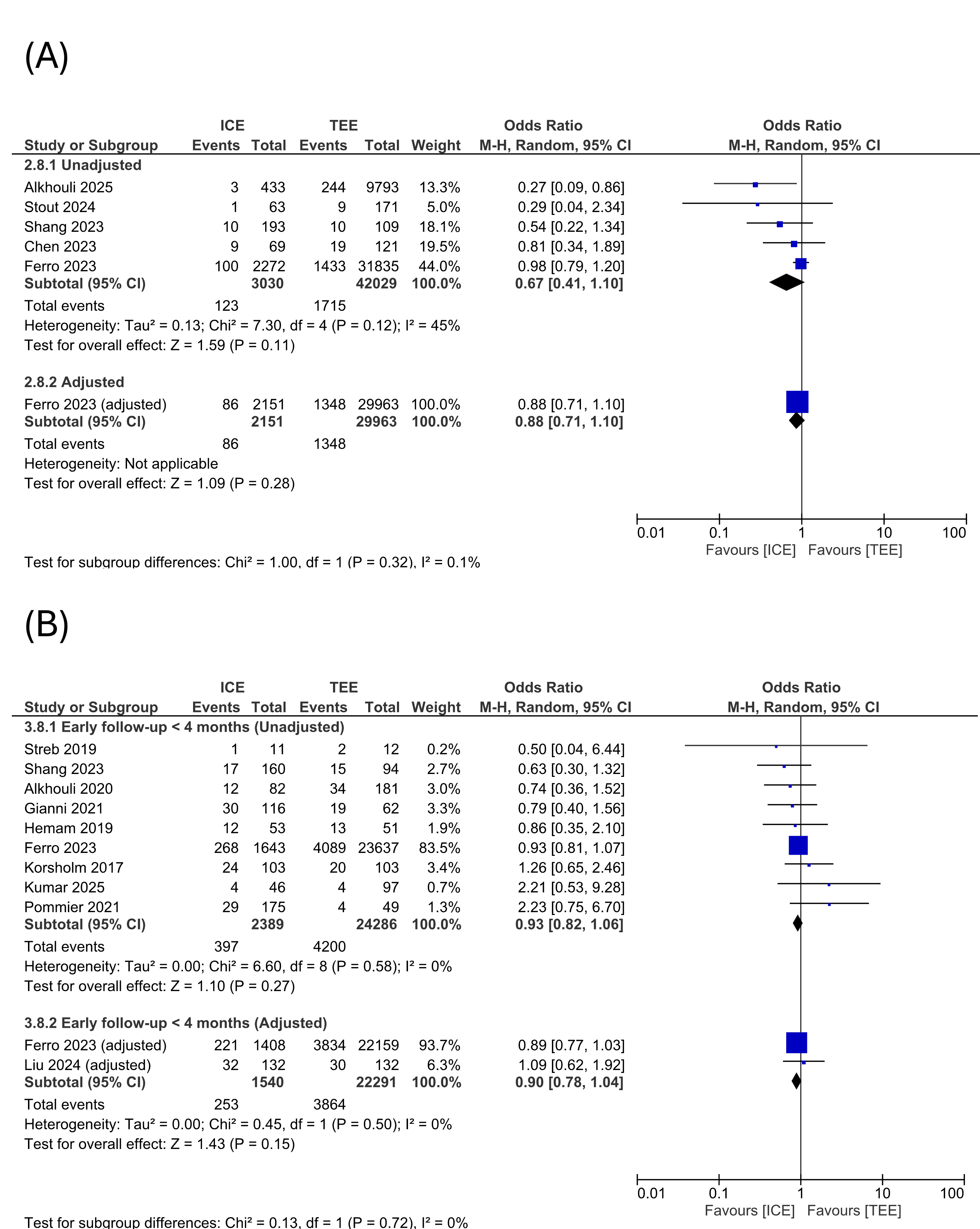


Supplemental Figure 11,Forest plot of odds ratios for ICE vs. TEE, A; peridevice leak<5mm (In-hospital complications), unadjusted 95% PI [0.23; 2.14], B; peridevice leak<5mm (Follow-up complications), early unadjusted 95% PI [0.84; 1.06], early adjusted 95% PI [0.42; 1.99].


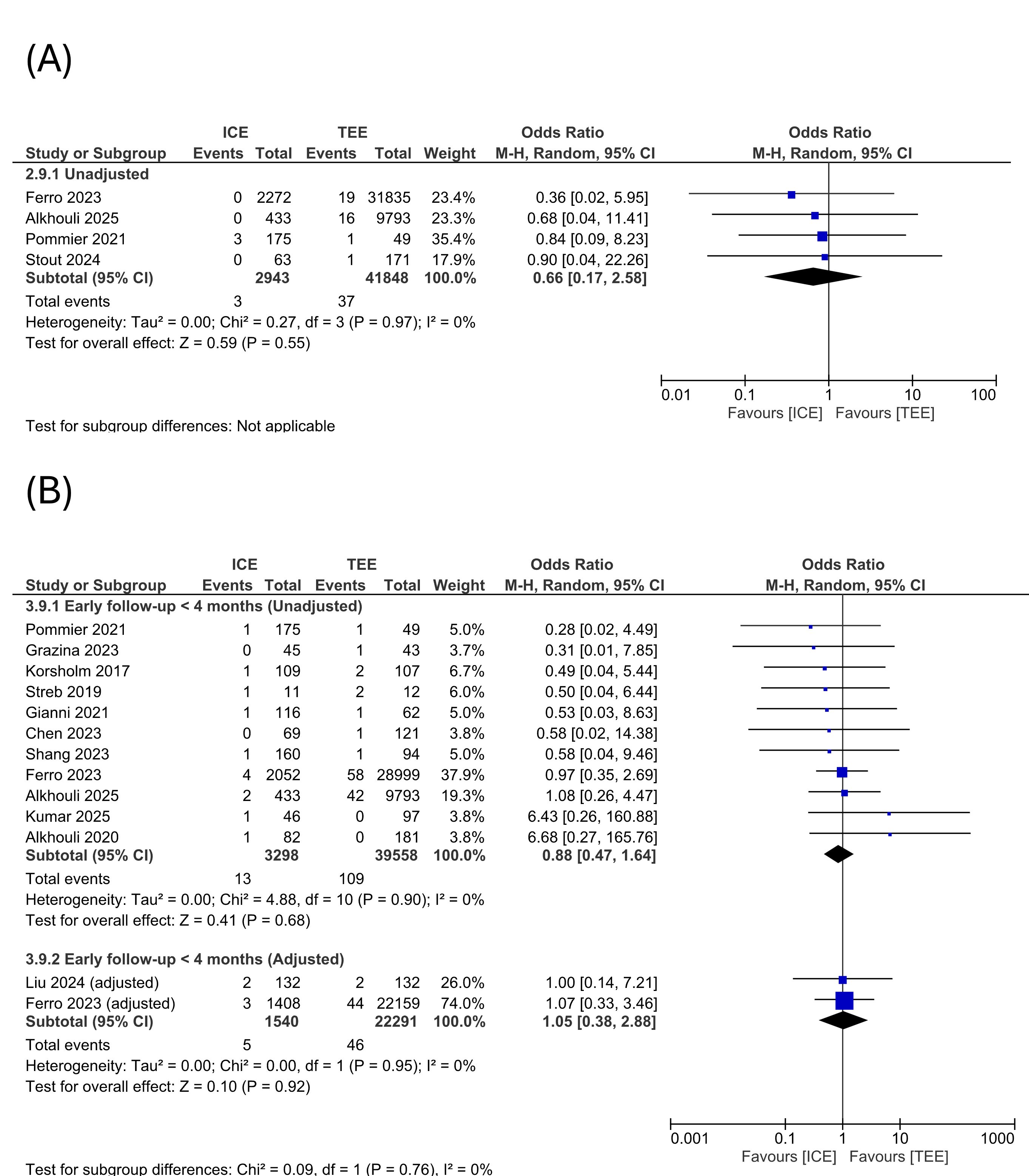


Supplemental Figure 12, Forest plot of odds ratios for ICE vs. TEE, A; Device related thrombus (In-hospital complications), unadjusted 95% PI [0.07; 5.95], B; Device related thrombus (Follow-up complications), early unadjusted 95% PI [0.43; 1.76], early adjusted 95% PI [0.01; 695.40].


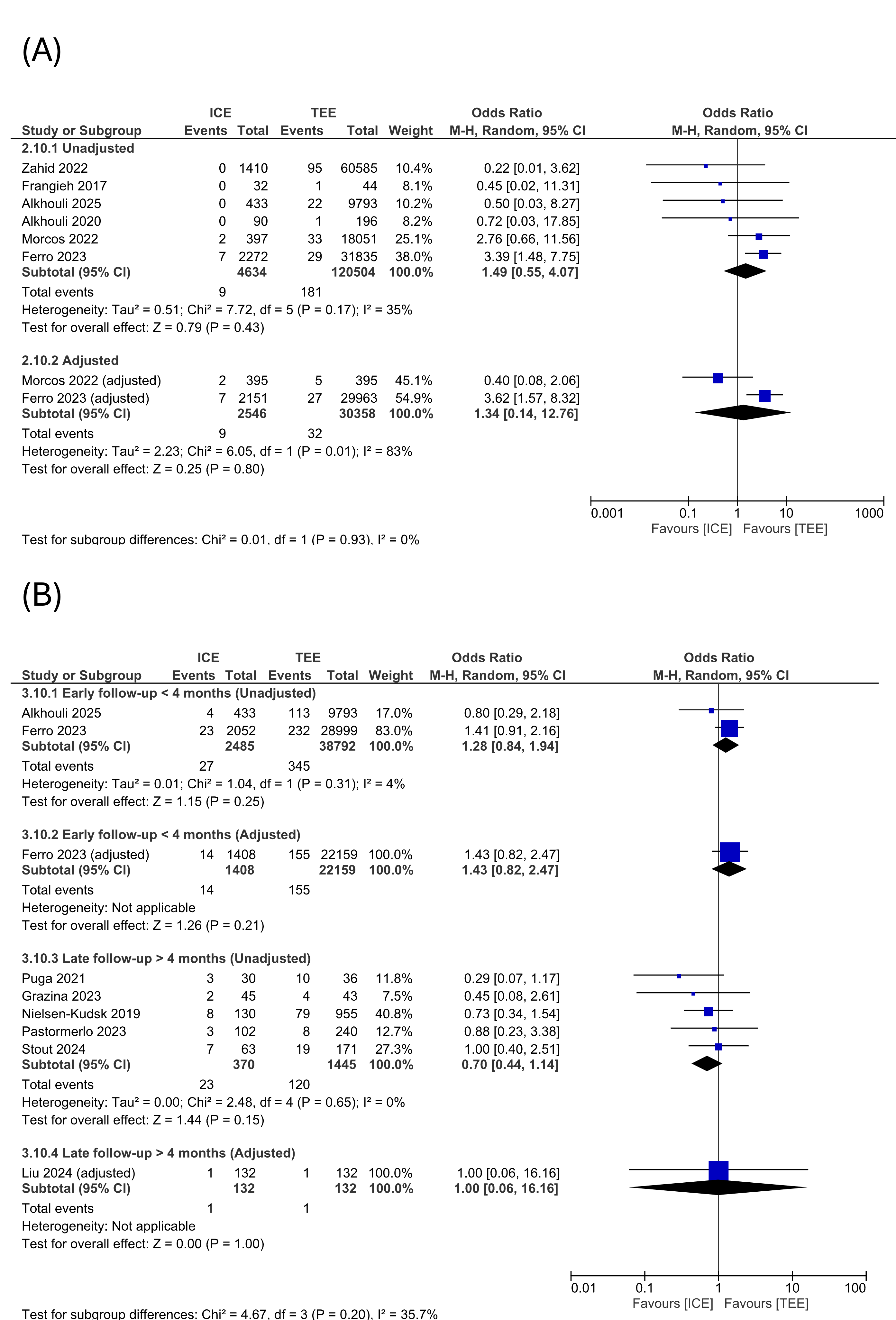


Supplemental Figure 13, Forest plot of odds ratios for ICE vs. TEE, A; All-cause mortality (In-hospital complications), unadjusted 95% PI [0.16; 14.14], adjusted 95% PI [0.00; 30913647970.18], B; All-cause mortality (Follow-up complications), early unadjusted 95% PI [0.07; 22.35], late unadjusted 95% PI [0.39; 1.35].


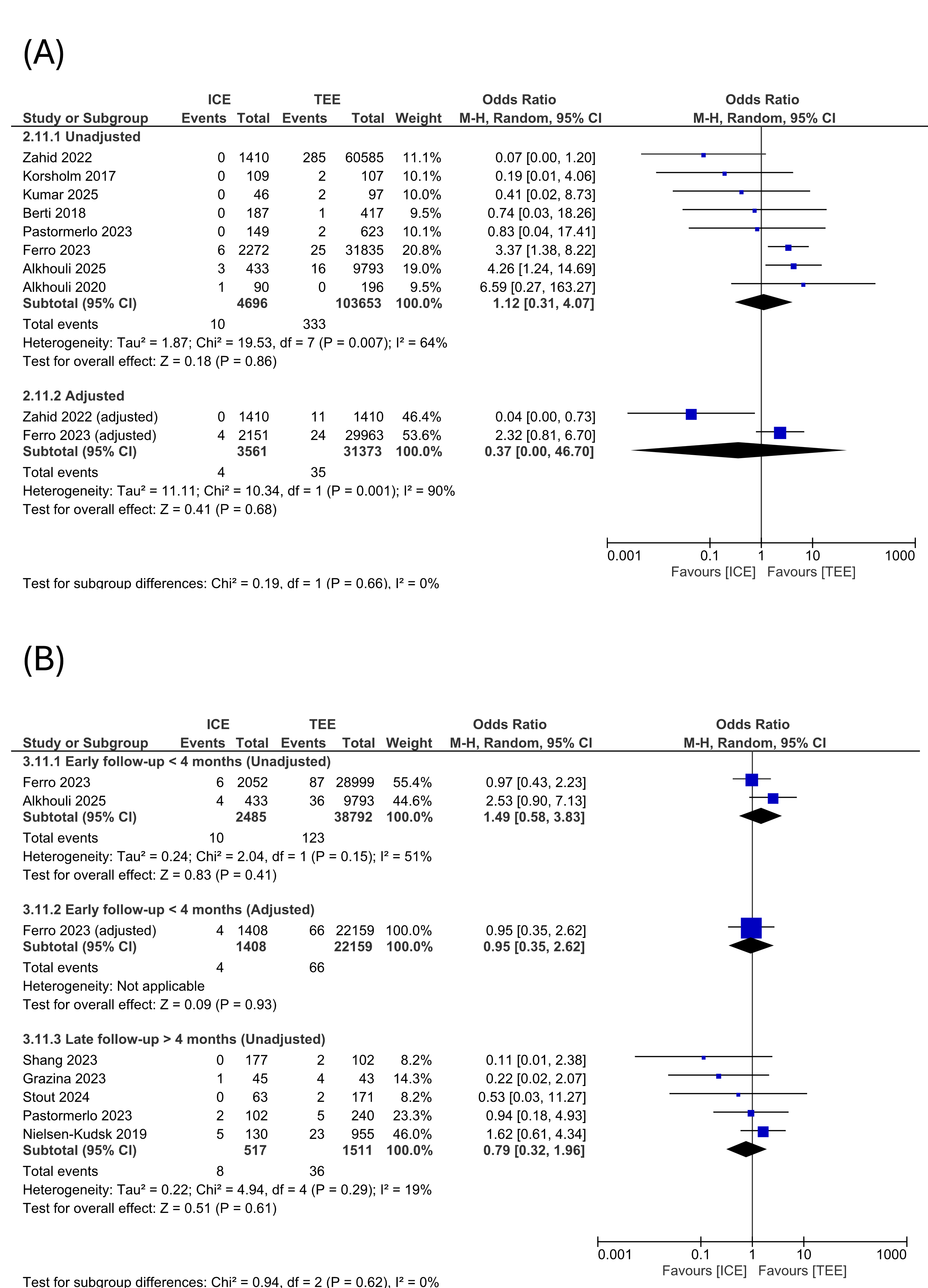


Supplemental Figure 14, Forest plot of odds ratios for ICE vs. TEE, A; Stroke (In-hospital complications), unadjusted 95% PI [0.03; 40.01], adjusted 95% PI [0.0000; 28025245064307551502466], B; Stroke (Follow-up complications), early unadjusted 95% PI [0.01; 8774.84], late unadjusted 95% PI [0.14; 4.73].


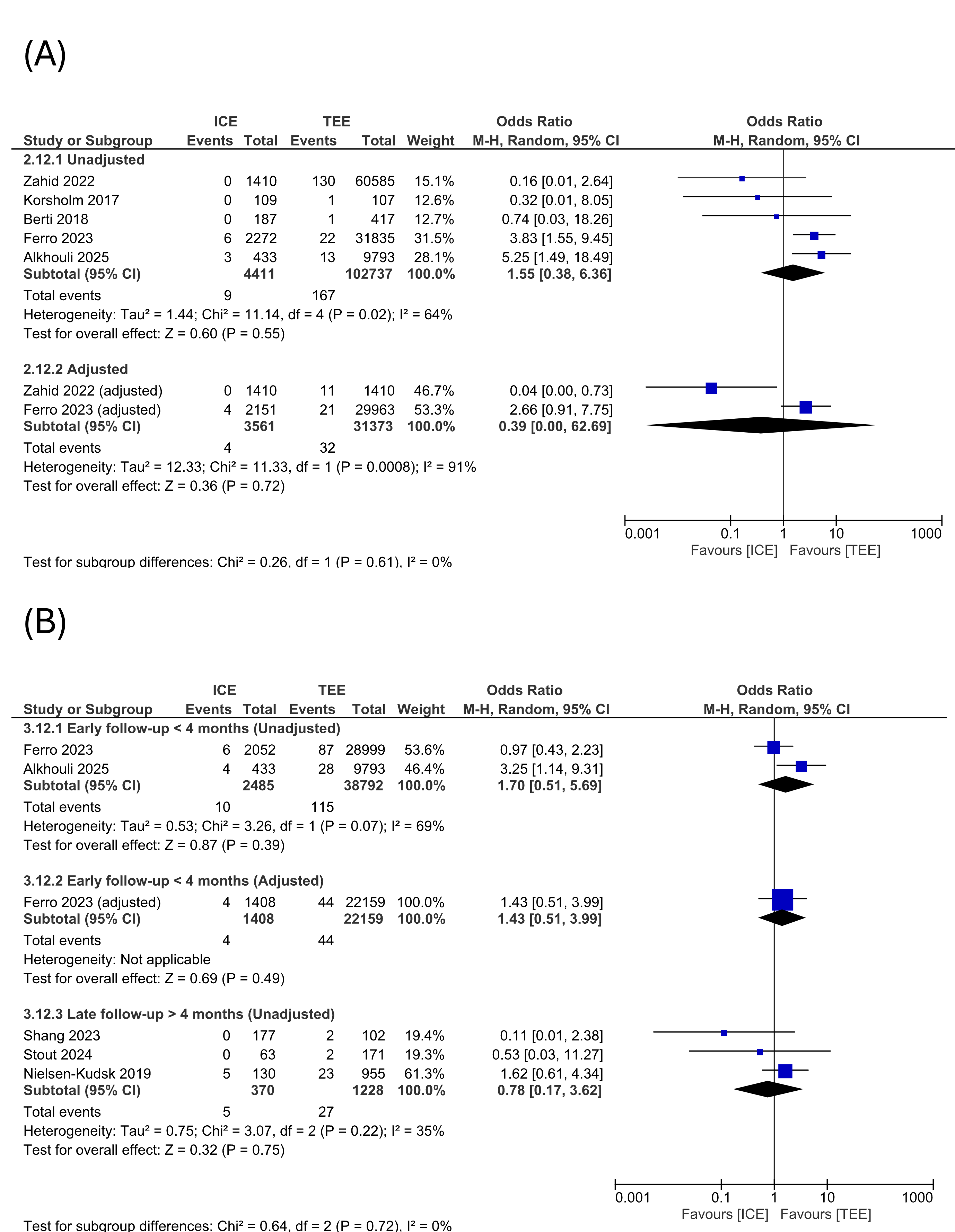


Supplemental Figure 15, Forest plot of odds ratios for ICE vs. TEE, A; Ischemic stroke (In-hospital complications), unadjusted 95% PI [0.03; 74.55], adjusted 95% PI , B; Ischemic stroke (Follow-up complications), early unadjusted 95% PI [0.00; 286810.64], late unadjusted 95% PI [0.01; 110.56].


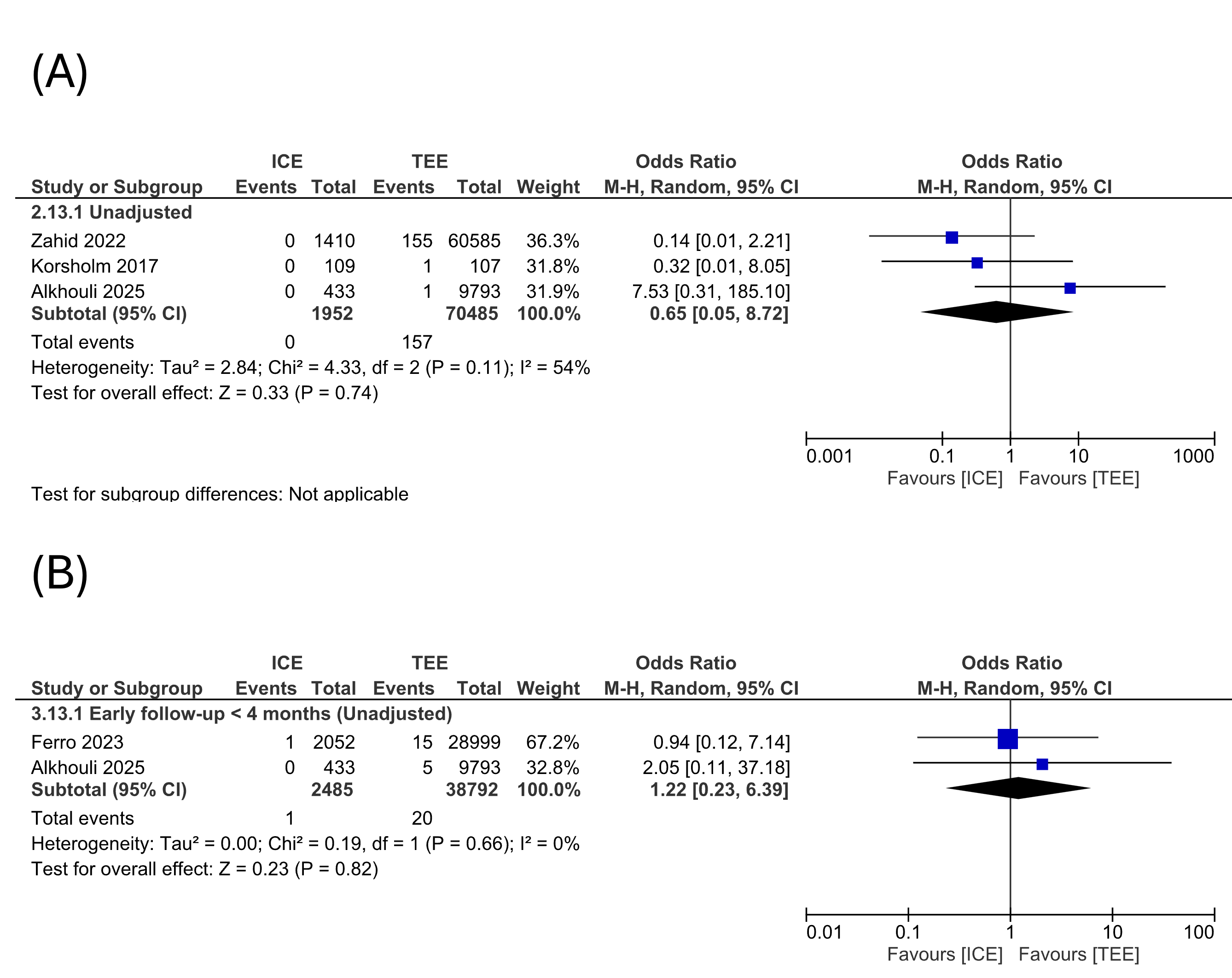


Supplemental Figure 16, Forest plot of odds ratios for ICE vs. TEE, A; Hemorrhagic stroke (In-hospital complications), unadjusted 95% PI [0.01; 6443.68], B; Hemorrhagic stroke (Follow-up complications), early unadjusted 95% PI [0.00; 56805.60].


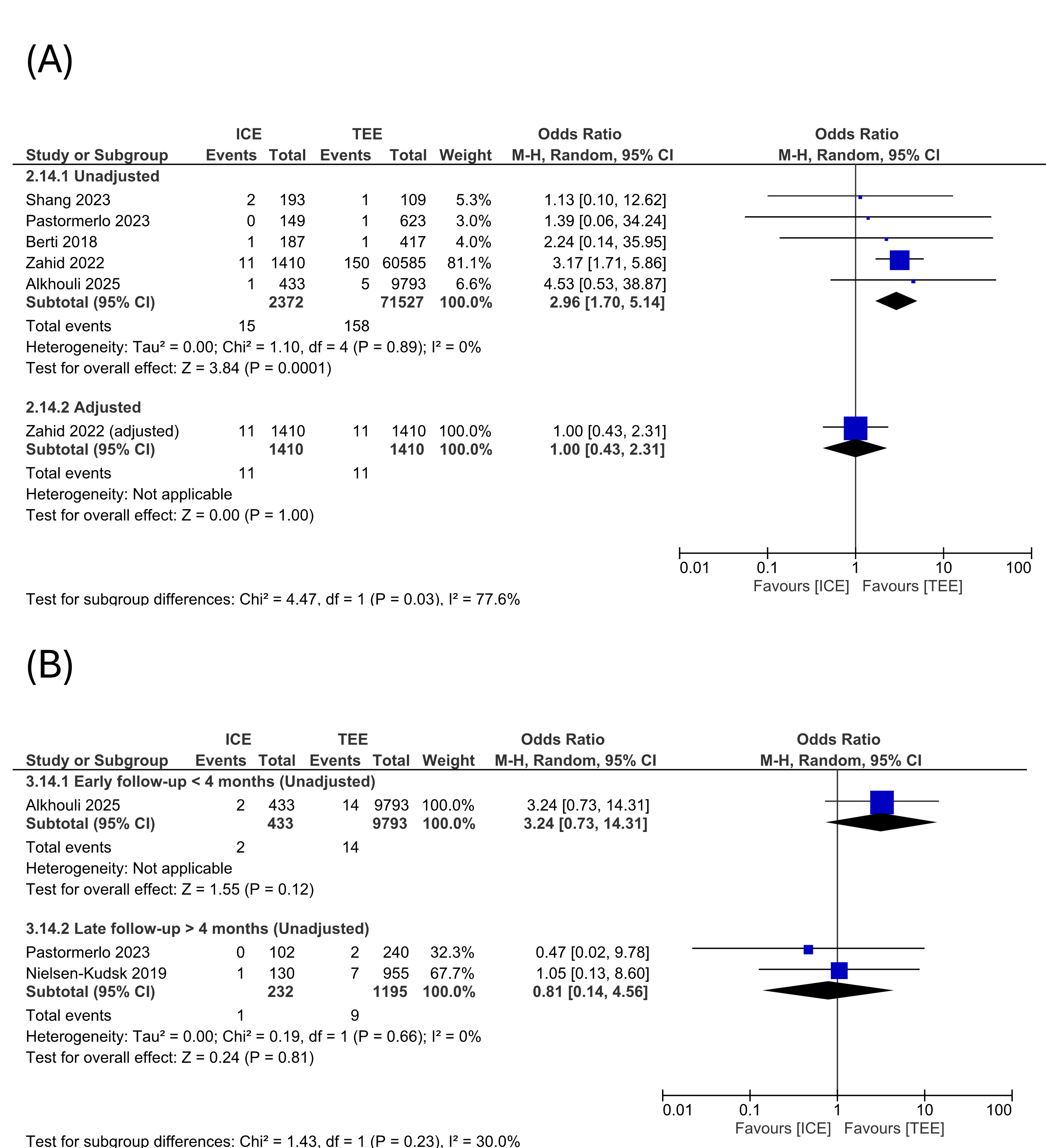


Supplemental Figure 17, Forest plot of odds ratios for ICE vs. TEE, A; Transient Ischemic Attack (In-hospital complications), unadjusted 95% PI [1.35; 6.41], B; Transient Ischemic Attack (Follow-up complications), late unadjusted 95% PI [0.00; 55795.58].


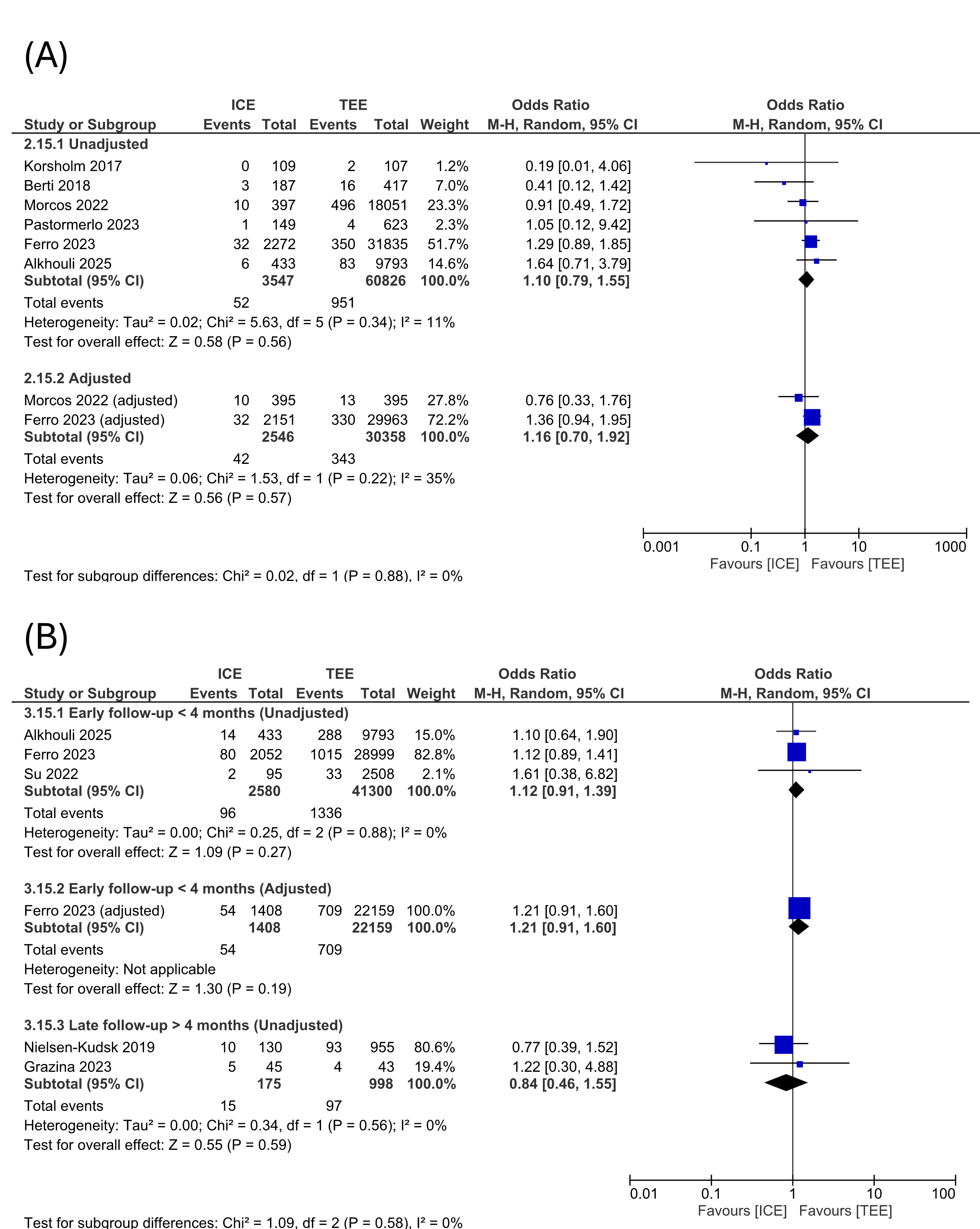


Supplemental Figure 18, Forest plot of odds ratios for ICE vs. TEE, A; Major bleeding (In-hospital complications), unadjusted 95% PI [0.62; 1.96], adjusted 95% PI [0.01; 94.91], B; Major bleeding (Follow-up complications), early unadjusted 95% PI [0.72; 1.75], late unadjusted 95% PI [0.02; 32.27].


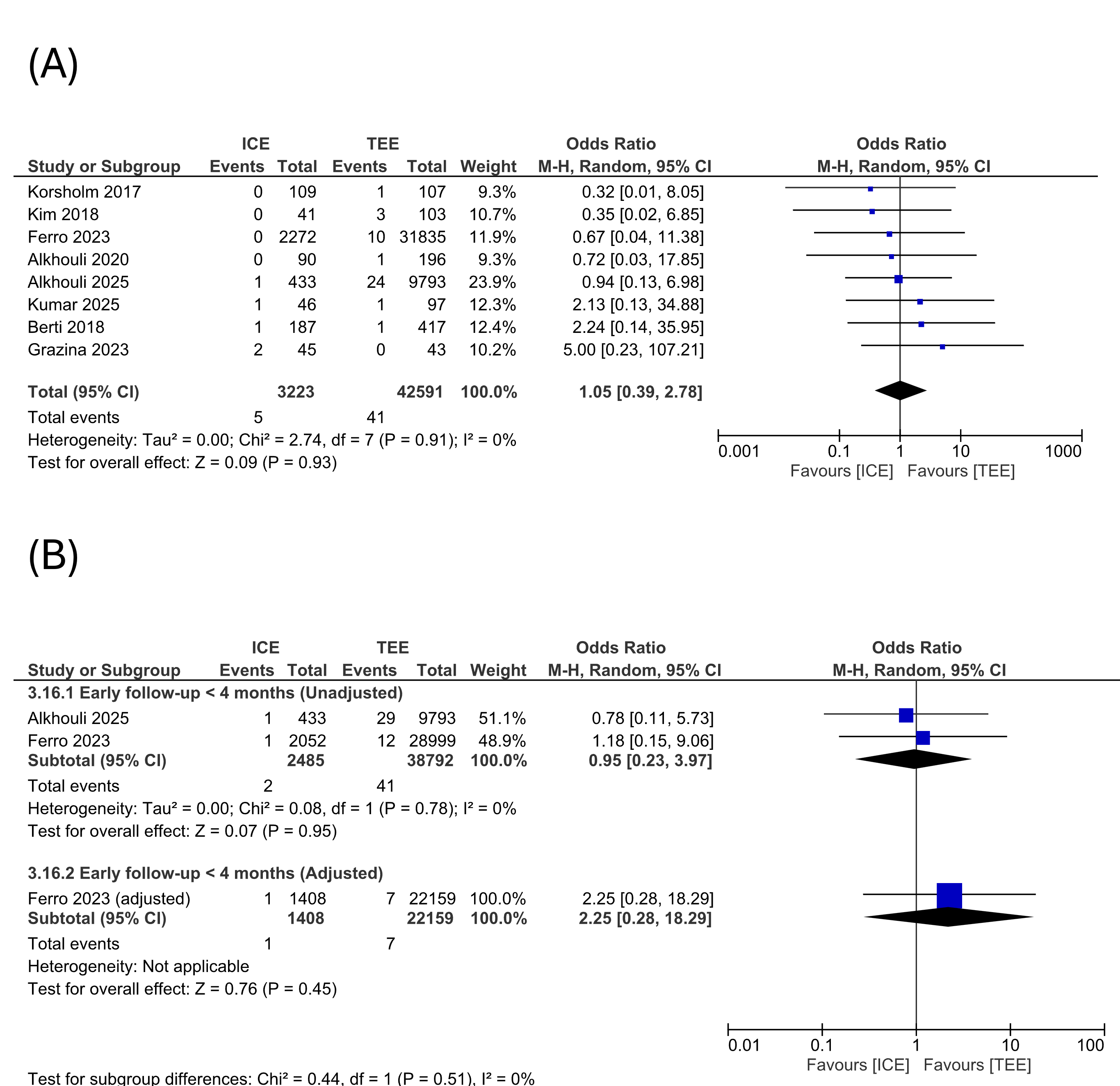


Supplemental Figure 19, Forest plot of odds ratios for ICE vs. TEE, A; Device embolization (In-hospital complications), unadjusted 95% PI [0.32; 3.38], B; Device embolization (Follow-up complications), early unadjusted 95% PI [0.01; 9788.34].


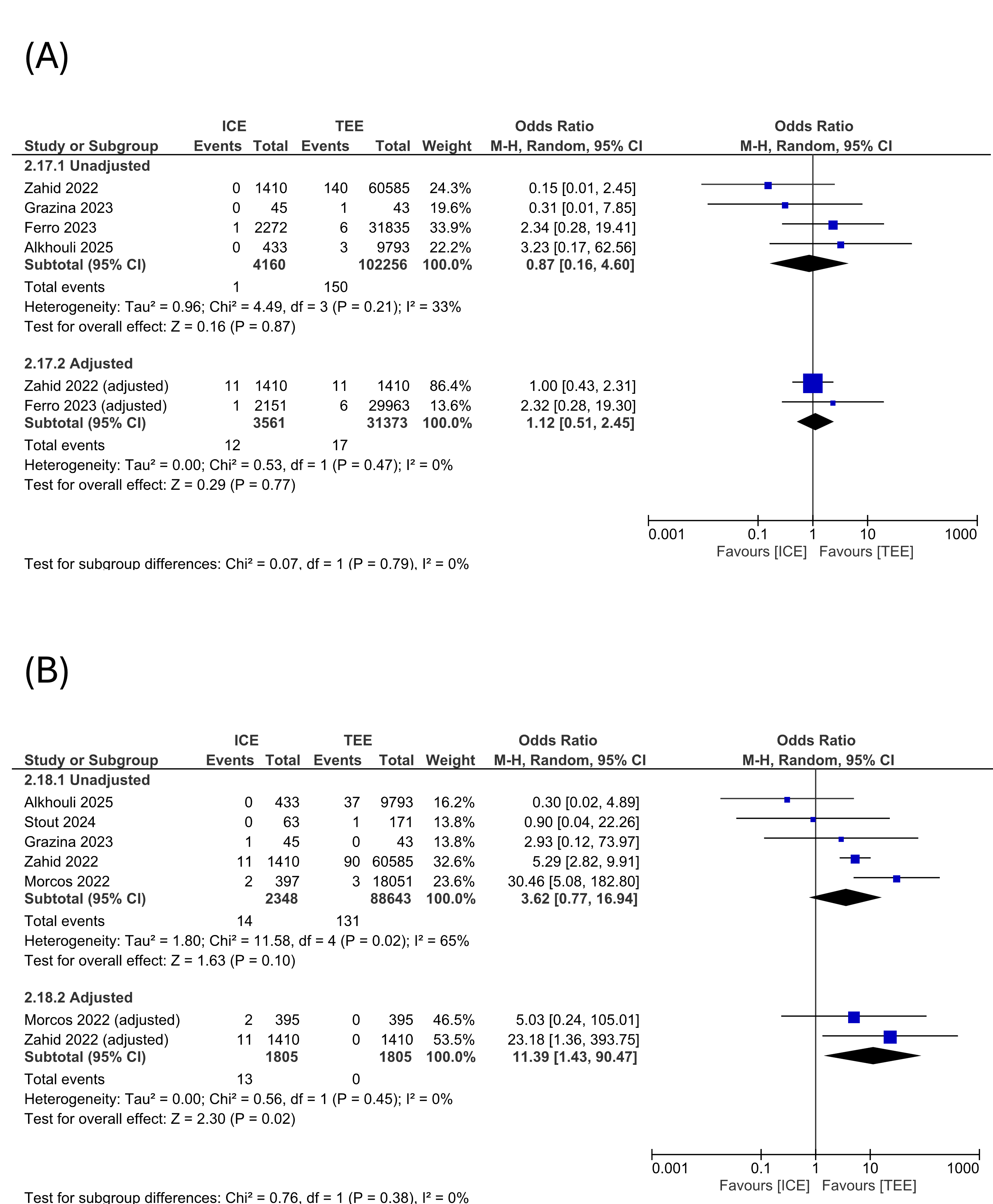


Supplemental Figure 20, Forest plot of odds ratios for ICE vs. TEE, A; Myocardial infarction (In-hospital complications), unadjusted 95% PI [0.01; 52.49], adjusted 95% PI [0.01; 169.88], B; Cardiac arrest (In-hospital complications), unadjusted 95% PI [0.05; 265.99], adjusted 95% PI [0.00; 7578010.01].


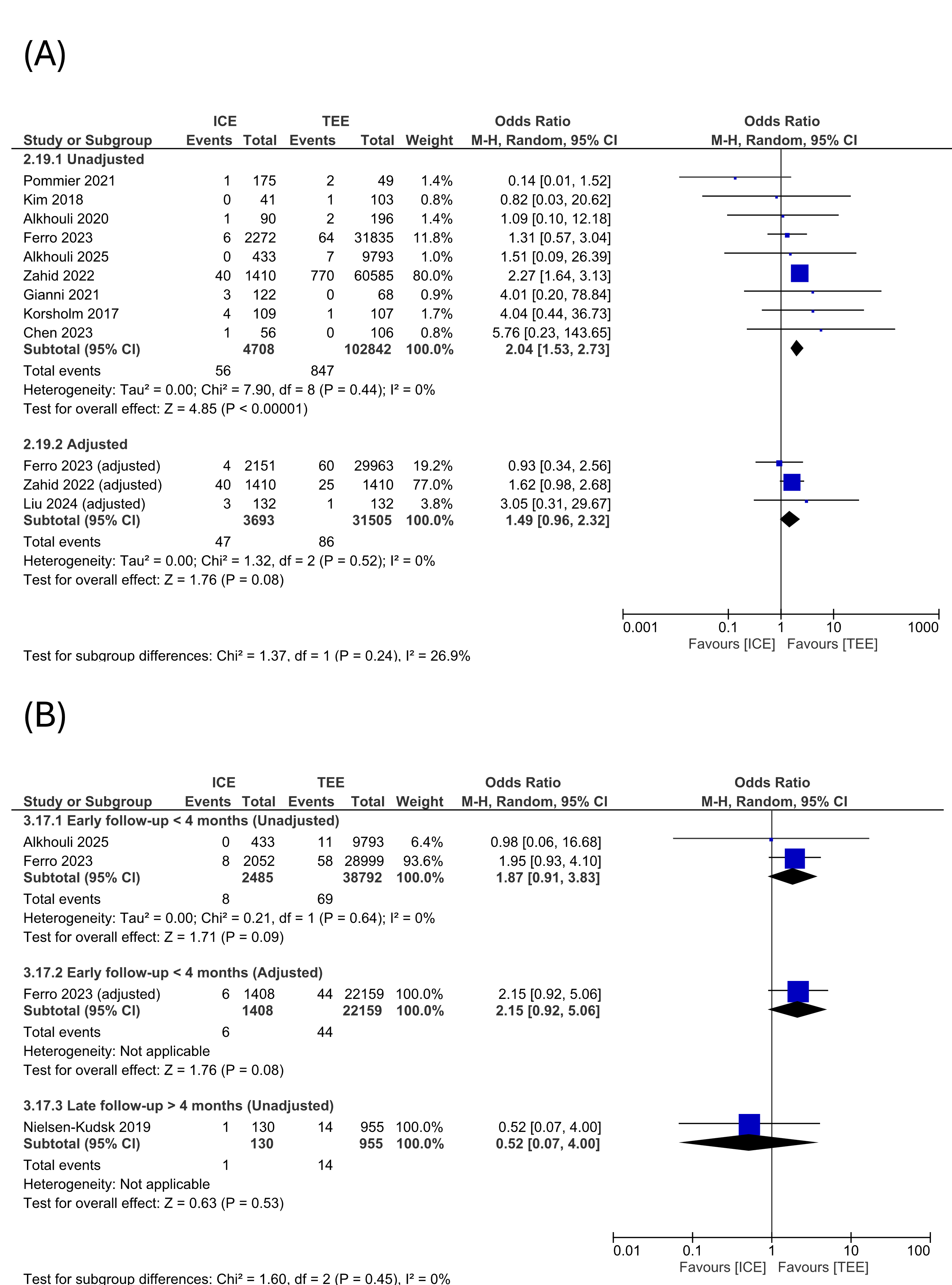


Supplemental Figure 21, Forest plot of odds ratios for ICE vs. TEE, A; Vascular complications (In-hospital complications), unadjusted 95% PI [1.45; 2.82], adjusted 95% PI [0.57; 3.85], B, Vascular complications (Follow-up complications), early unadjusted 95% PI [0.02; 191.02].


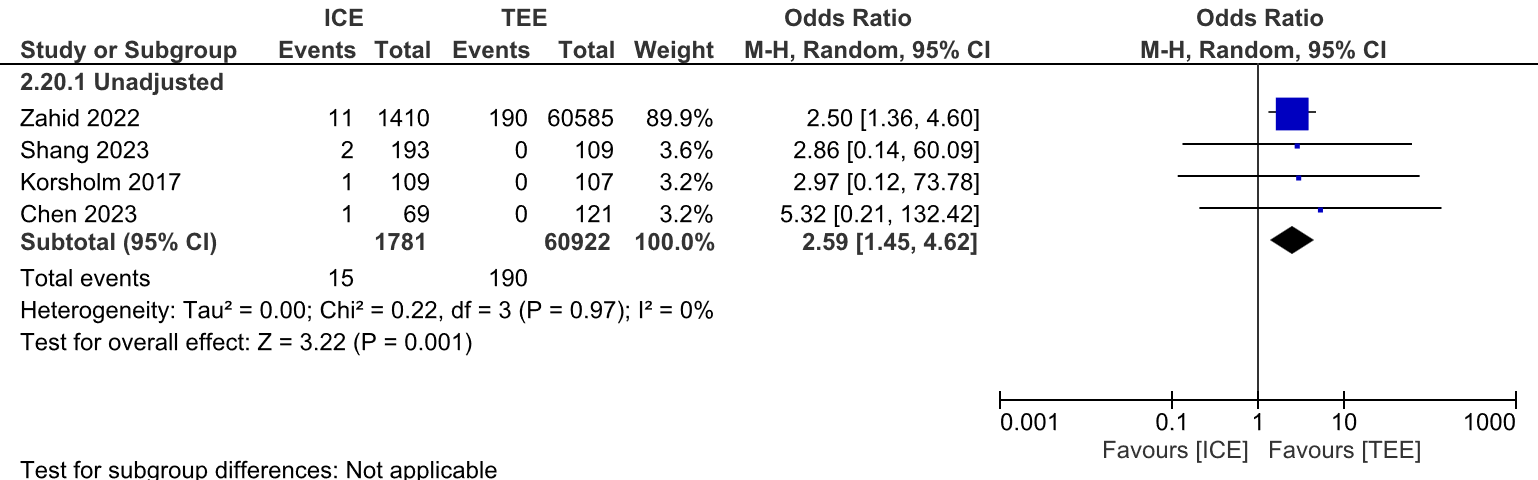


Supplemental Figure 22, Forest plot of odds ratios of Pseudoaneurysm (In-hospital complications) for ICE vs. TEE, unadjusted 95% PI [1.01; 6.54].


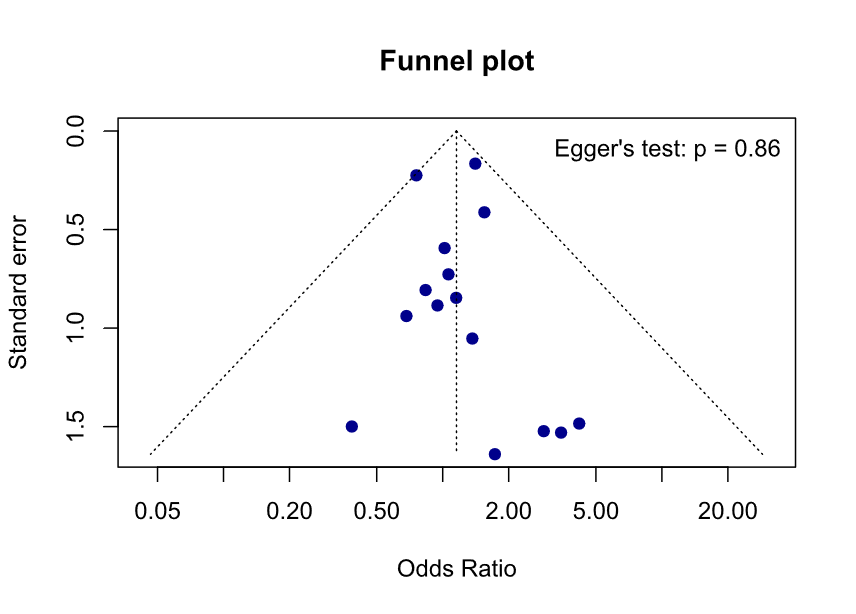


Figure 23, Funnel plot of procedural success.


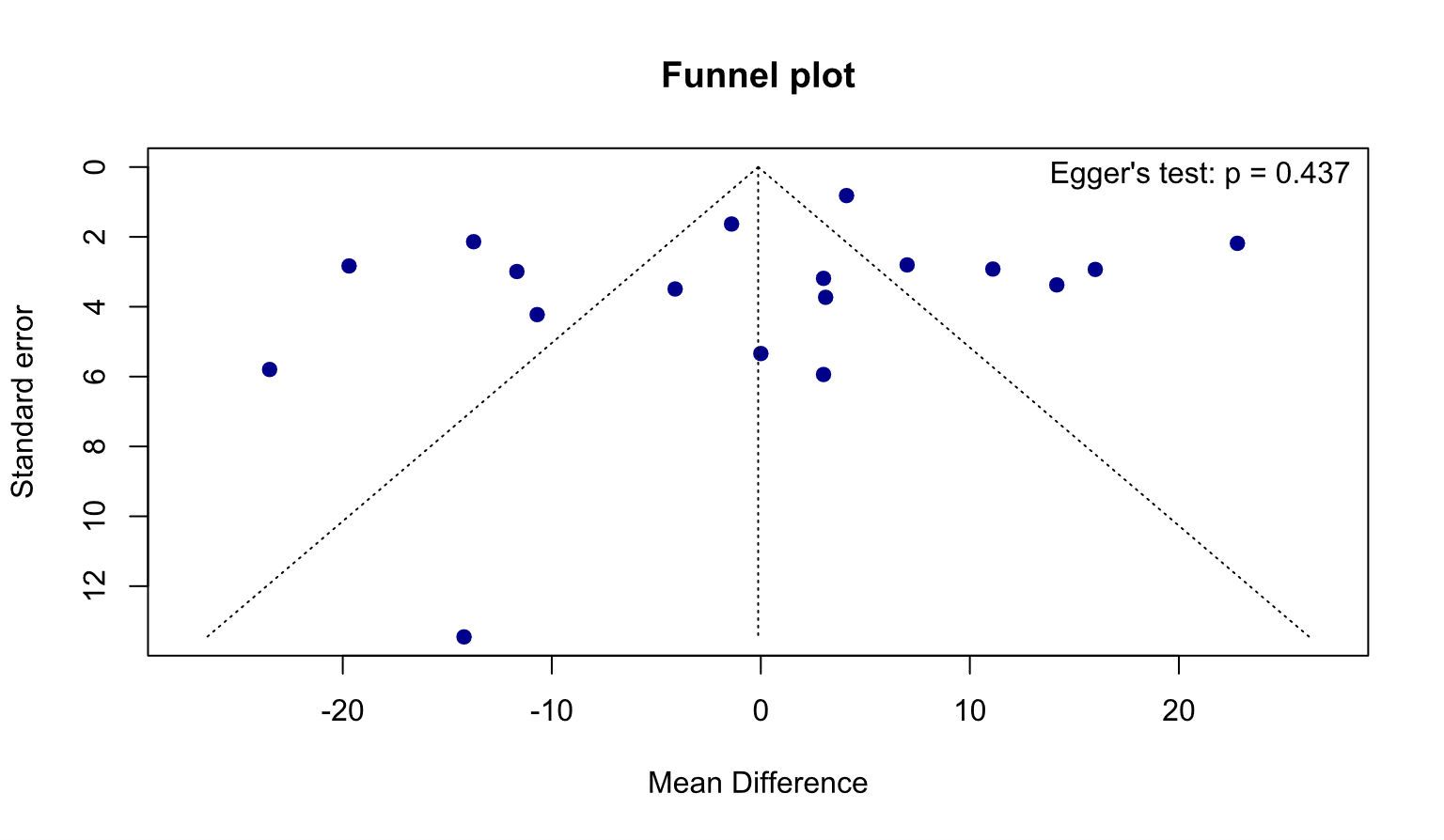


Supplemental Figure 24, Funnel plot of procedural time.


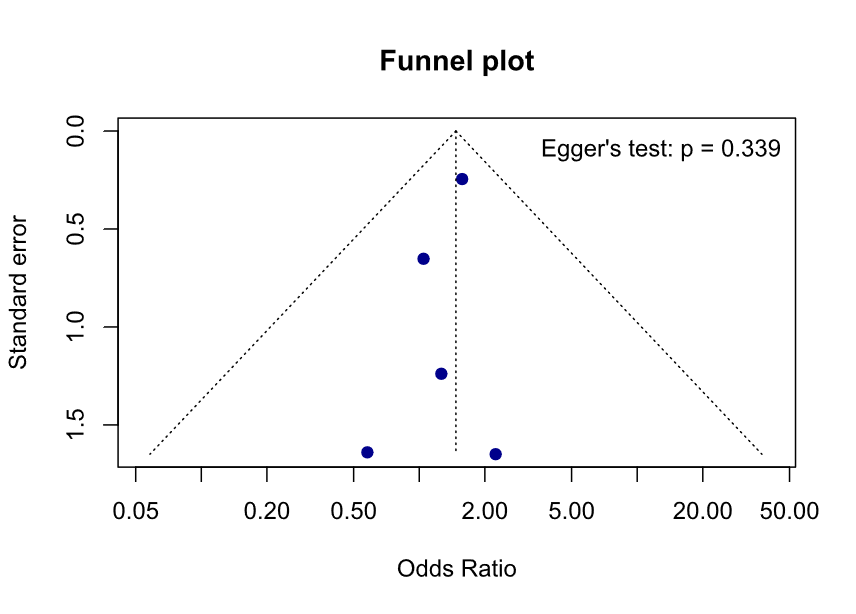


Supplemental Figure 25, Funnel plot of in-hospital overall PE.


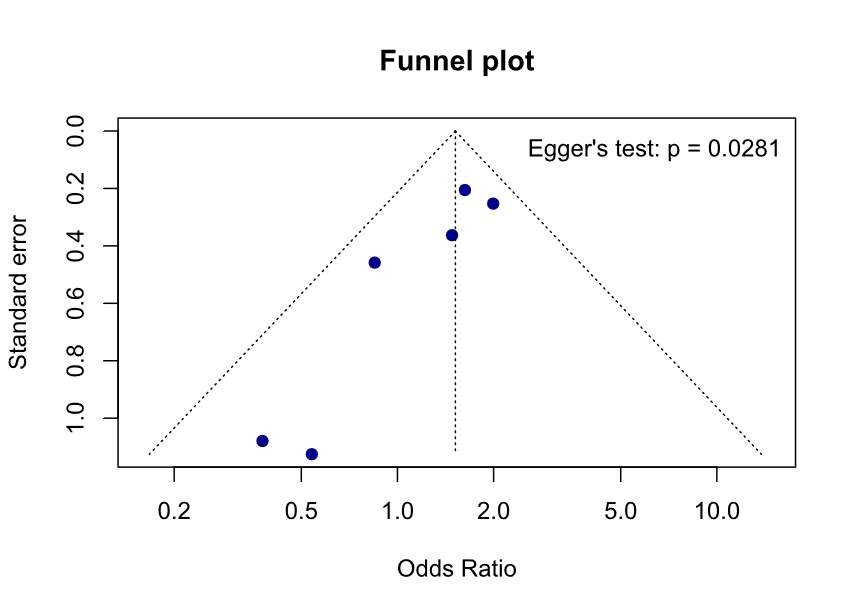


Supplemental Figure 26, Funnel plot of PE requiring intervention.


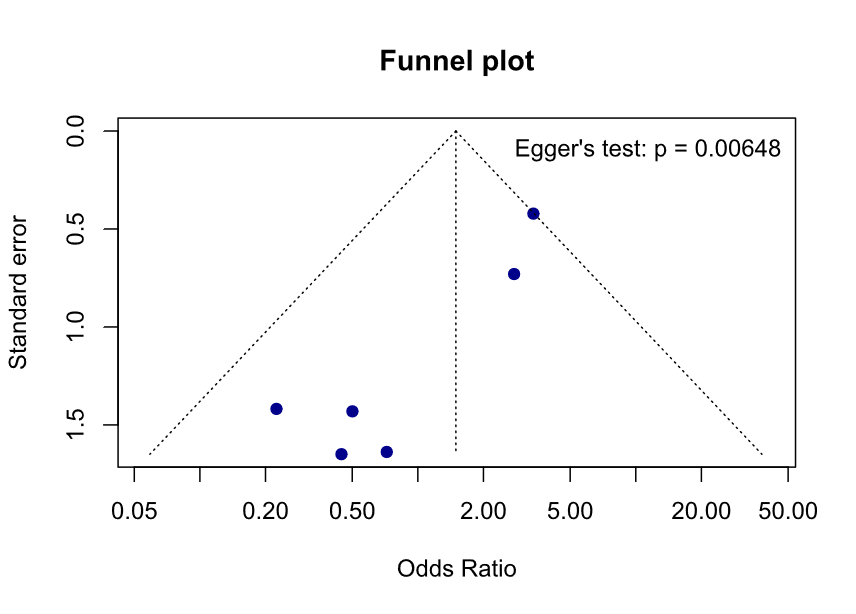


Supplemental Figure 27, Funnel plot of in-hospital all-cause mortality.


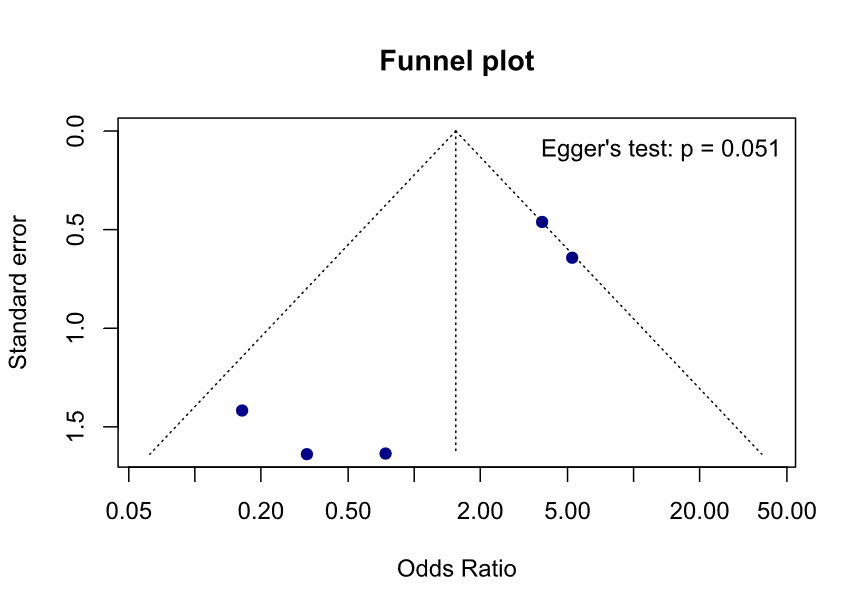


Supplemental Figure 28, Funnel plot of in-hospital ischemic stroke.


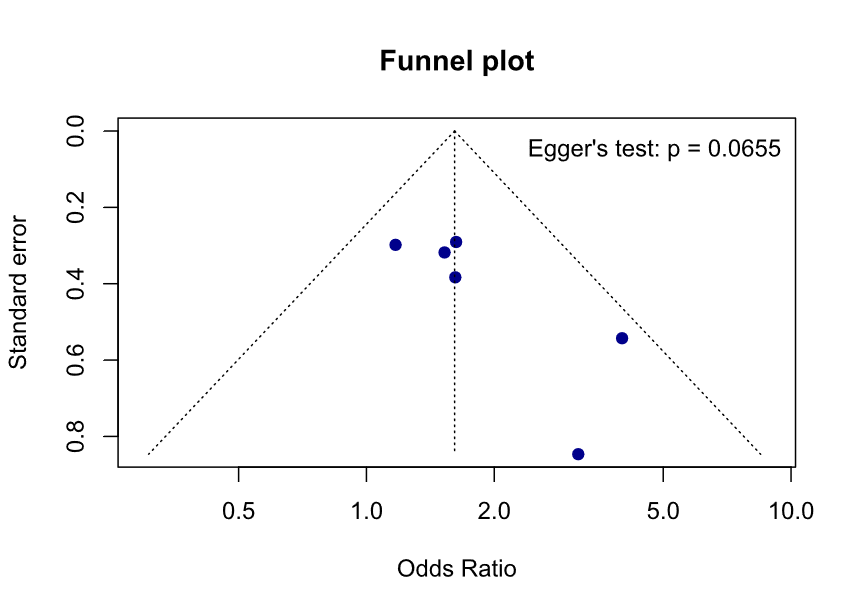


Supplemental Figure 29, Funnel plot of early follow-up residual iASD.


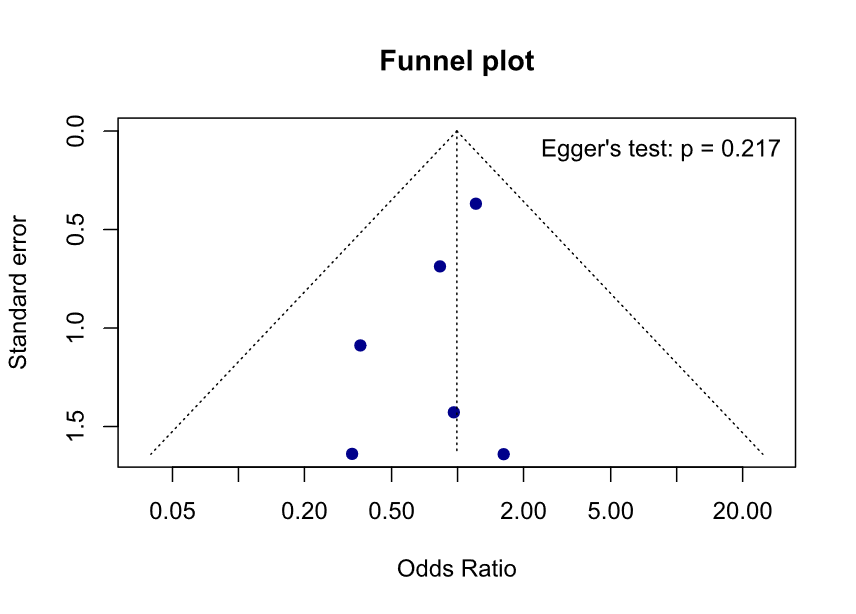


Supplemental Figure 30, Funnel plot of early follow-up PDL > 5 mm.


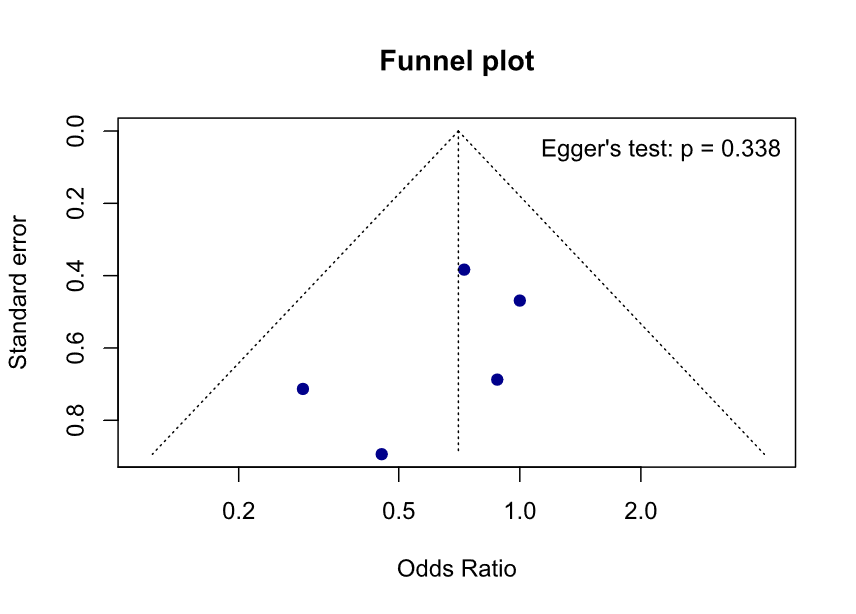


Supplemental Figure 31, Funnel plot of late follow-up all-cause mortality.


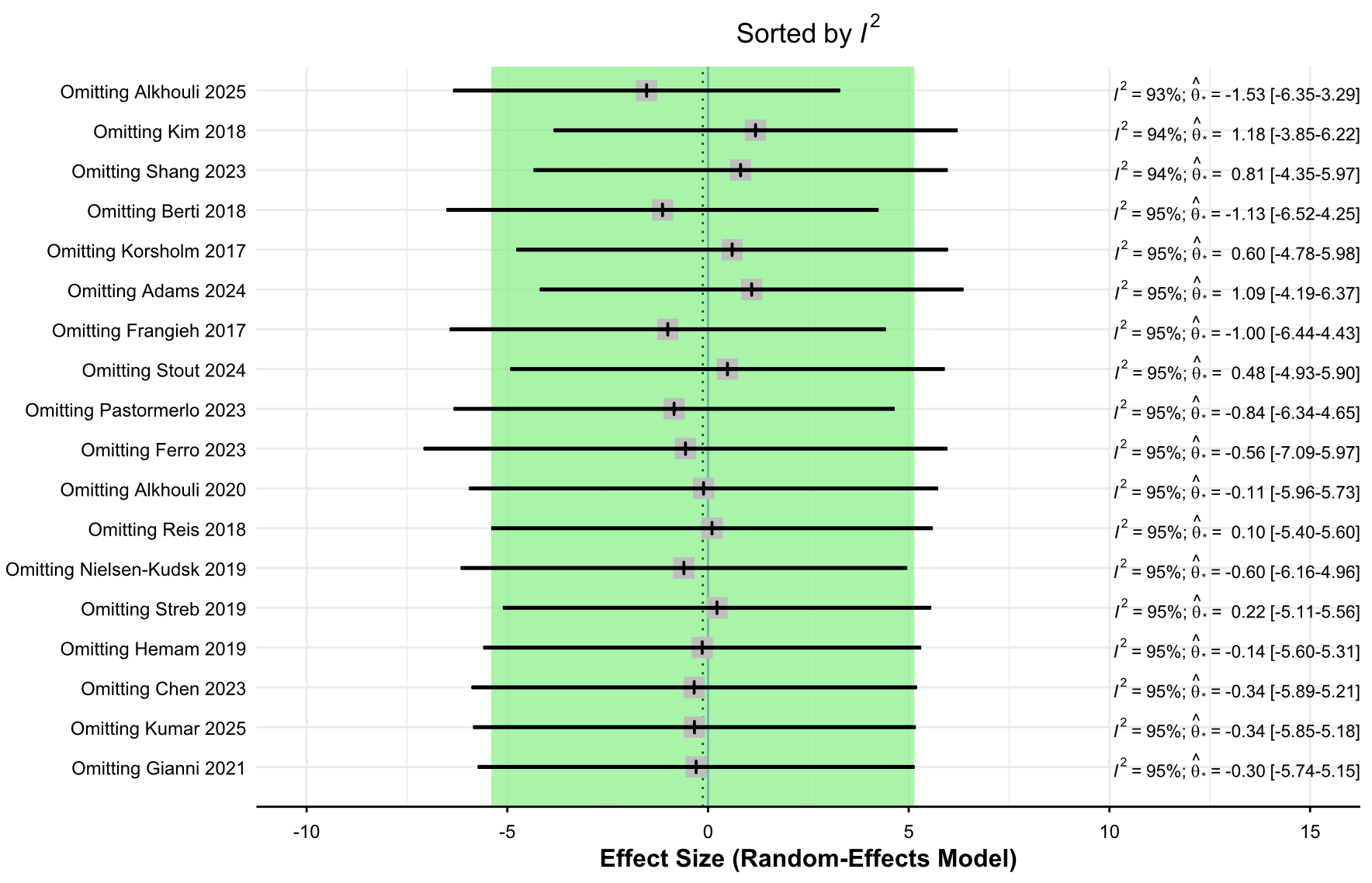


Supplemental Figure 32, Leave-one-out sensitivity analysis of unadjusted procedural time.


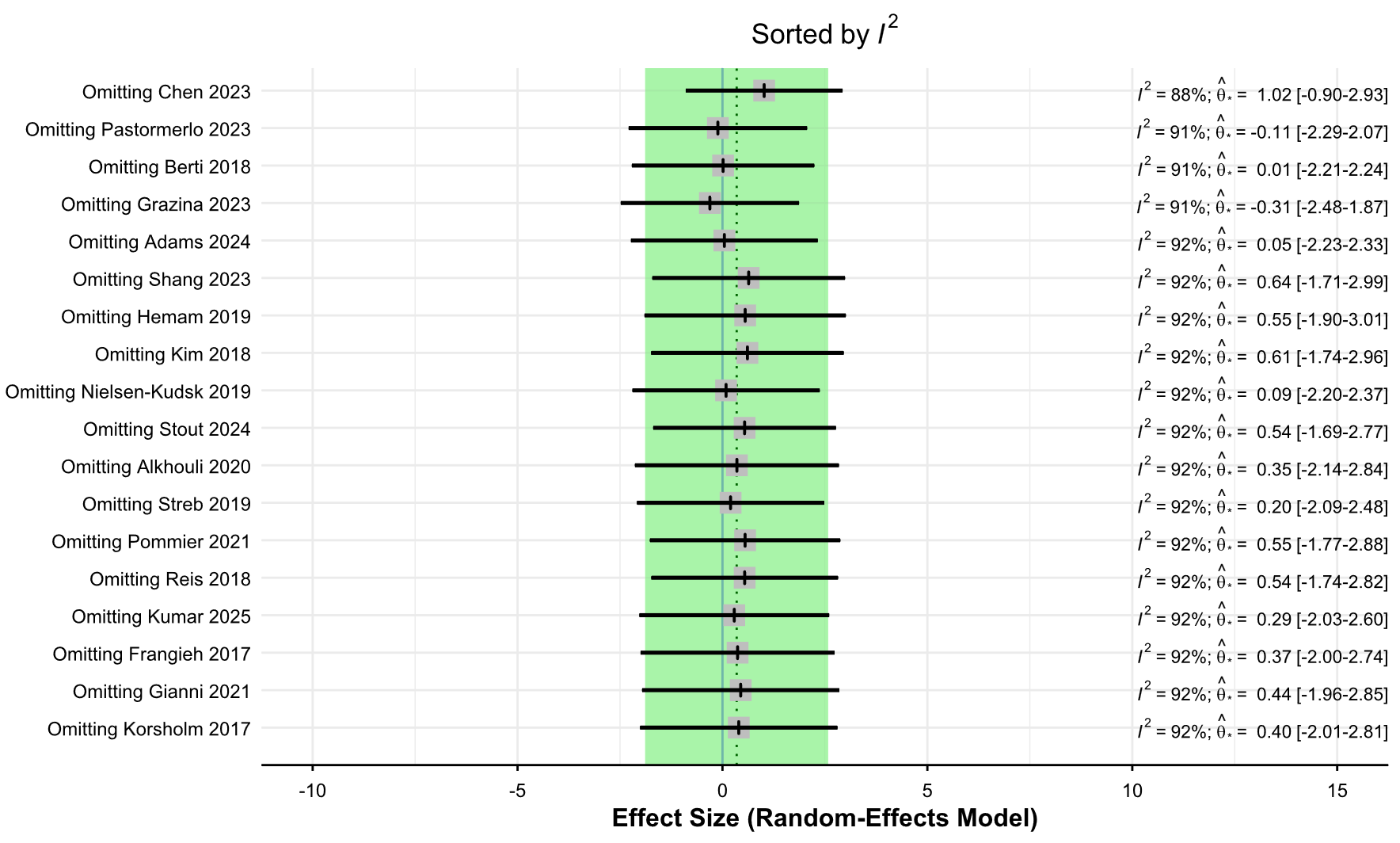


Supplemental Figure 33, Leave-one-out sensitivity analysis of unadjusted fluoroscopy time.


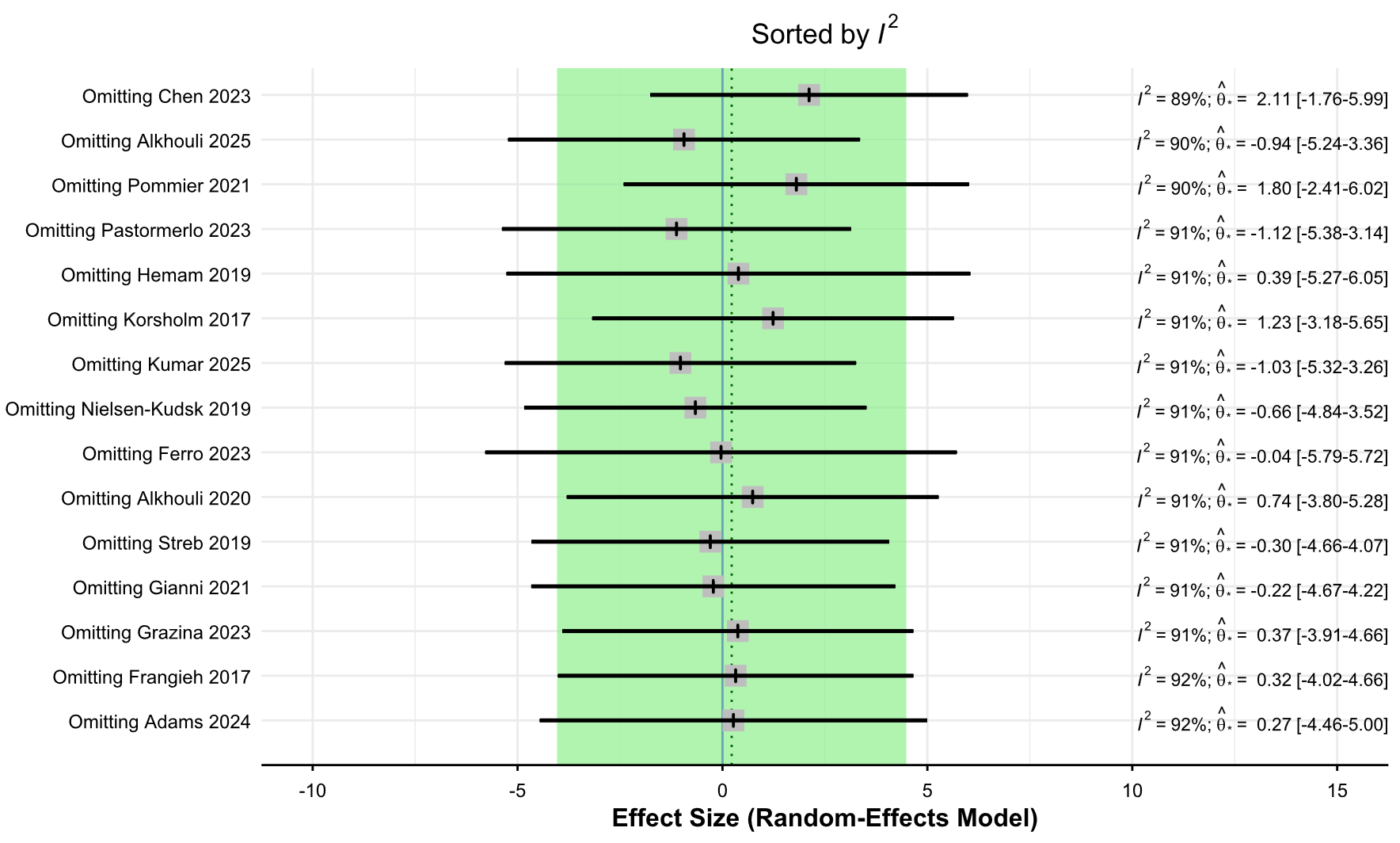


Supplemental Figure 34, Leave-one-out sensitivity analysis of unadjusted contrast volume.


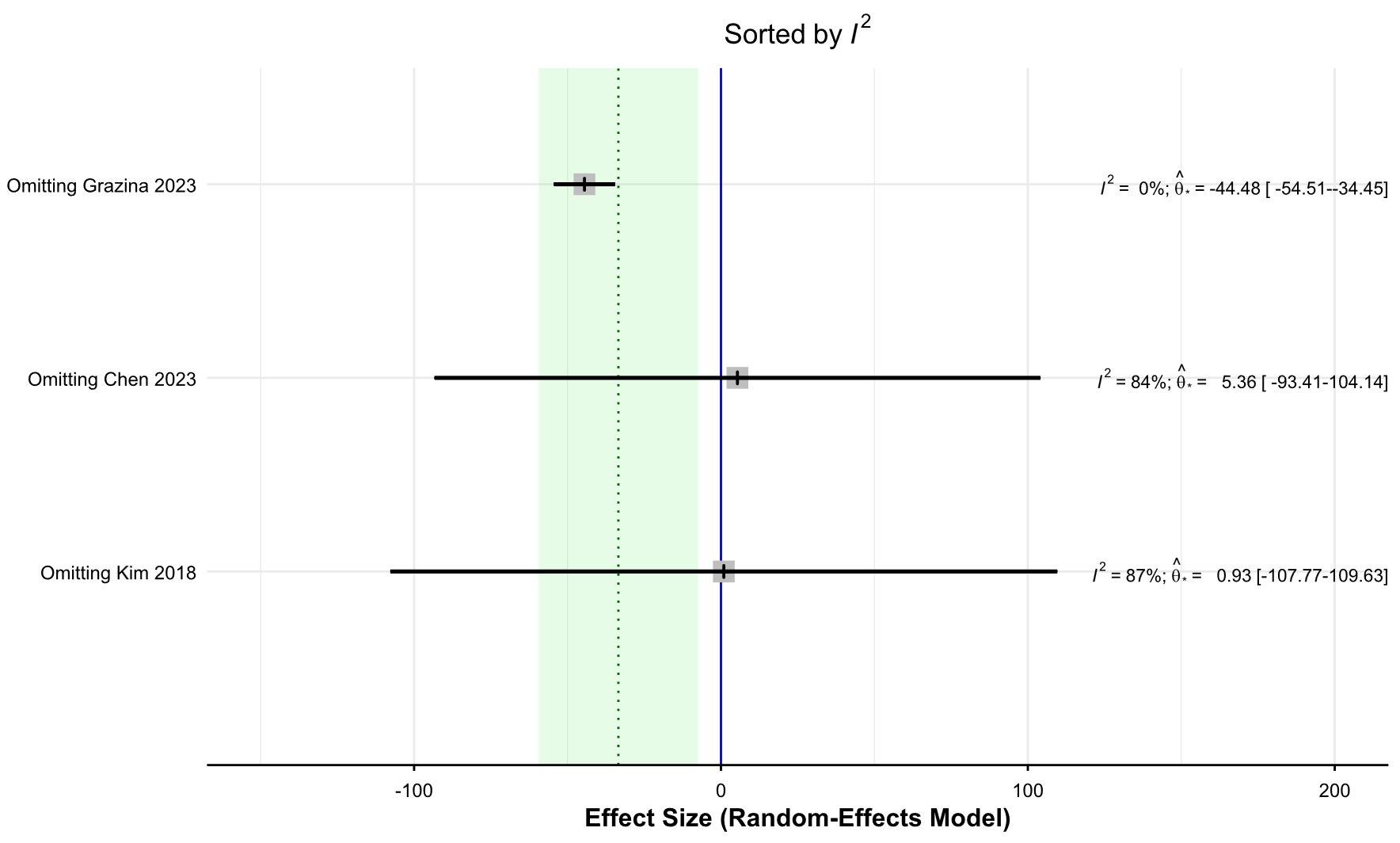


Supplemental Figure 35, Leave-one-out sensitivity analysis of unadjusted radiation dose.


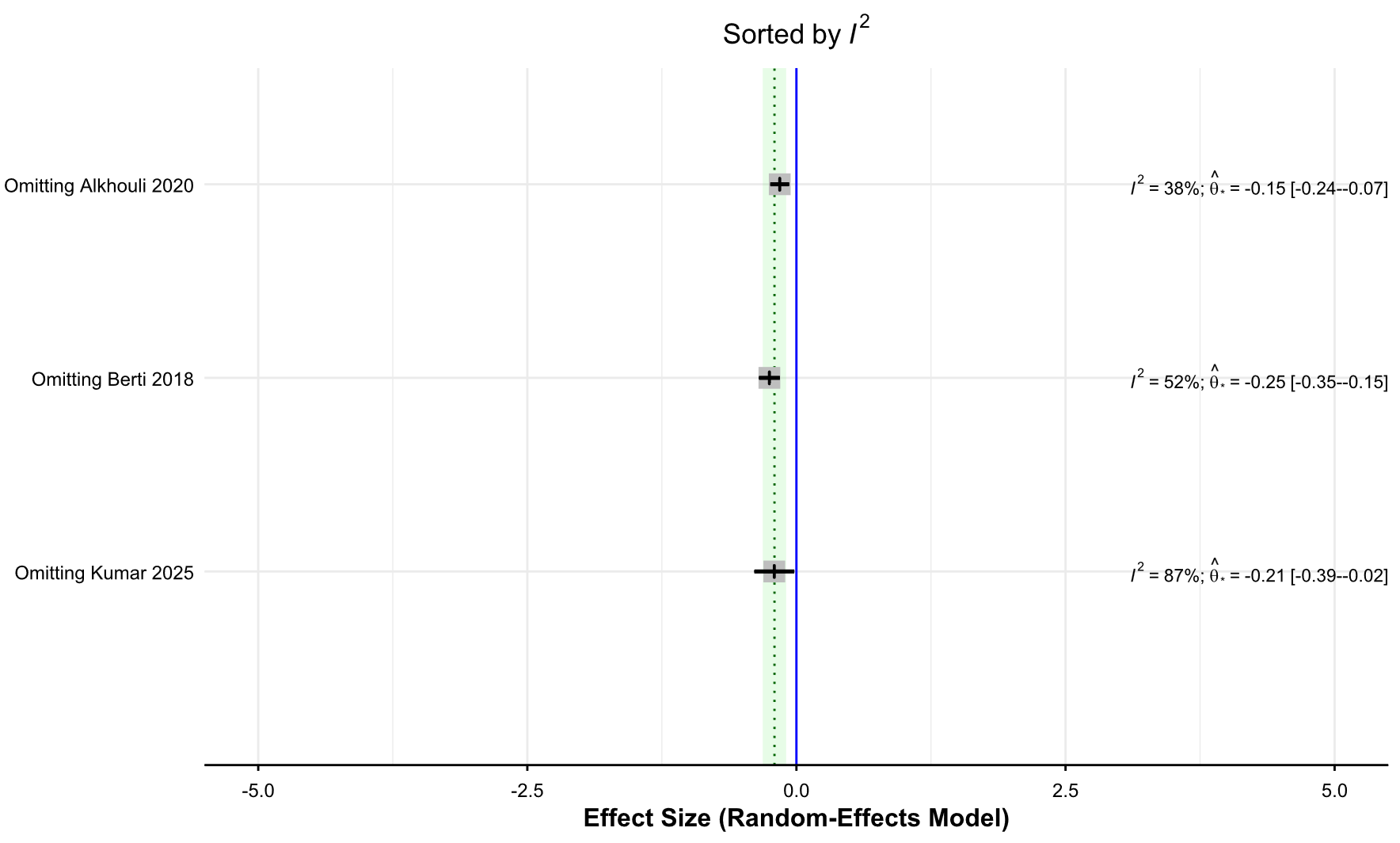


Supplemental Figure 36, Leave-one-out sensitivity analysis of unadjusted device attempted.


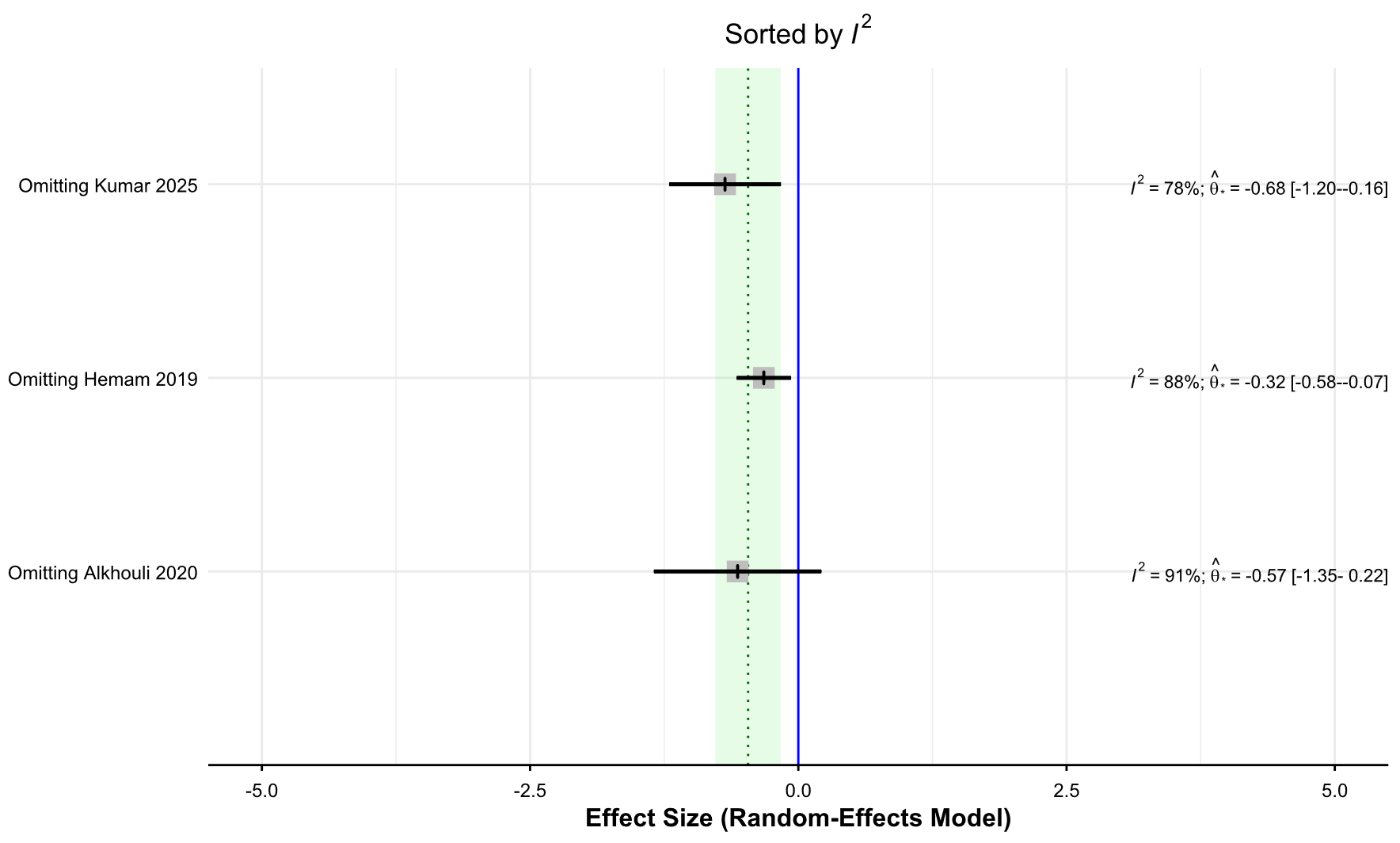


Supplemental Figure 37, Leave-one-out sensitivity analysis of unadjusted device recapture.


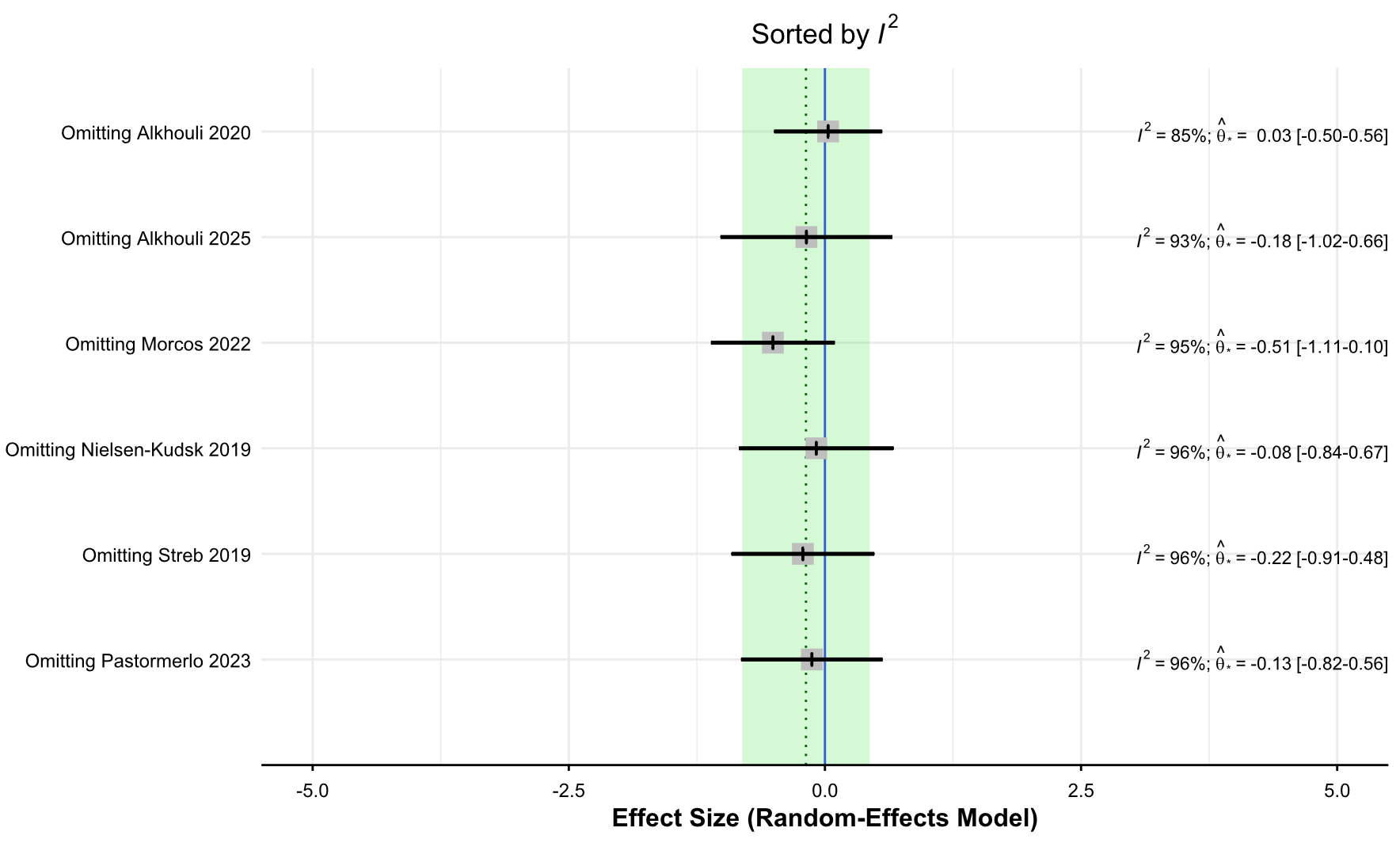


Supplemental Figure 38, Leave-one-out sensitivity analysis of unadjusted length of hospital stay.


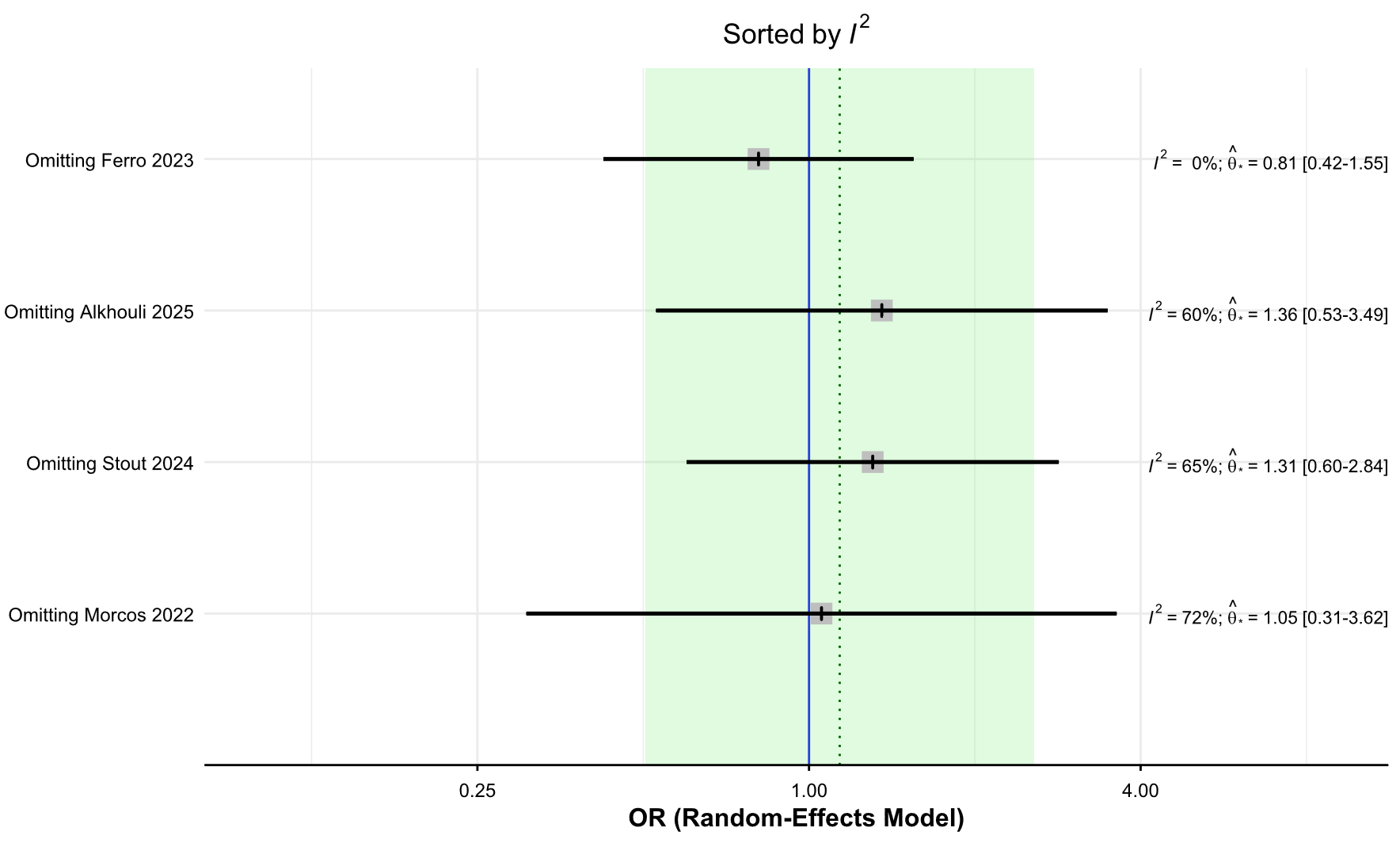


Supplemental Figure 39, Leave-one-out sensitivity analysis of unadjusted PE requiring pericardiocentesis.


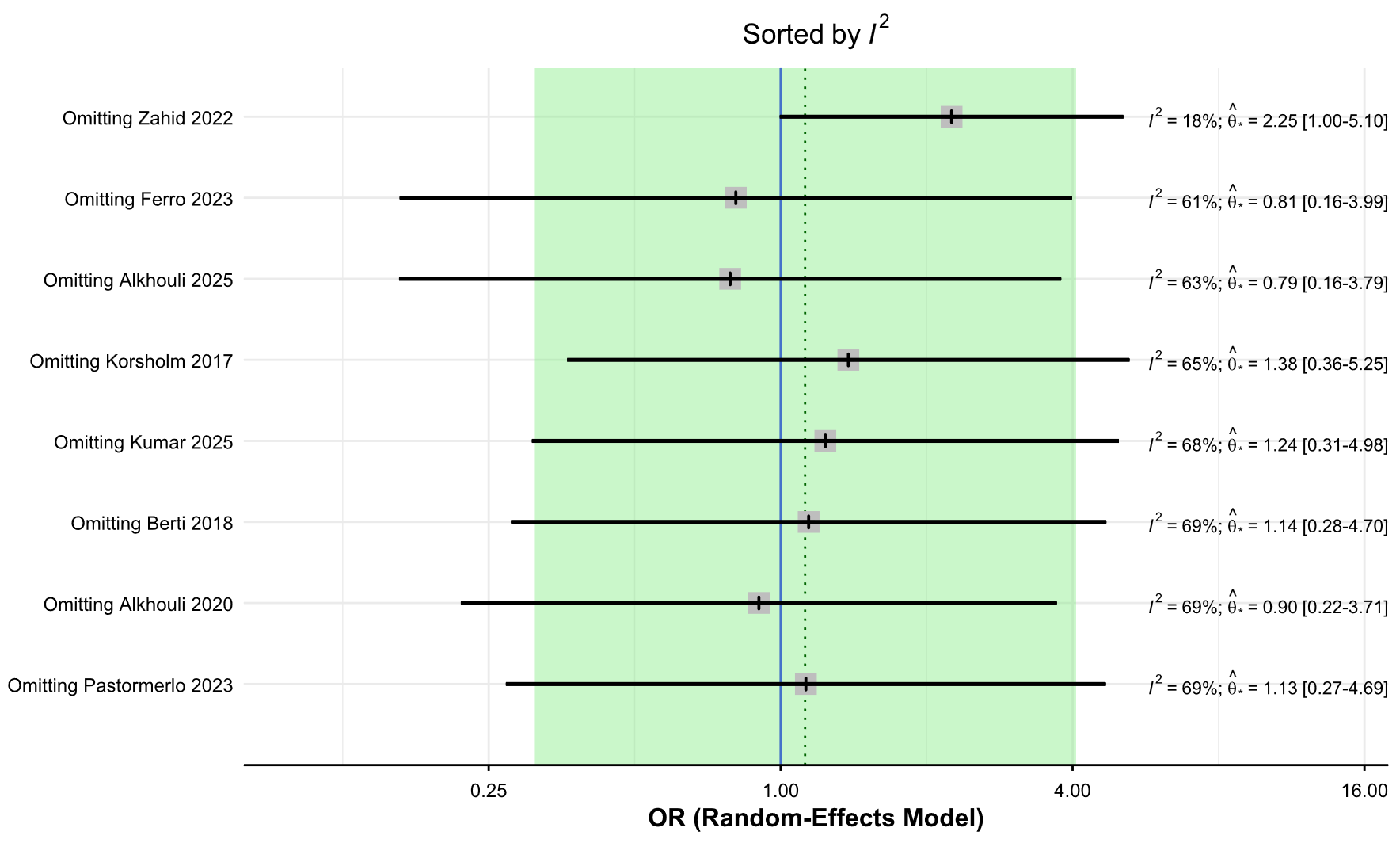


Supplemental Figure 40, Leave-one-out sensitivity analysis of unadjusted stroke.


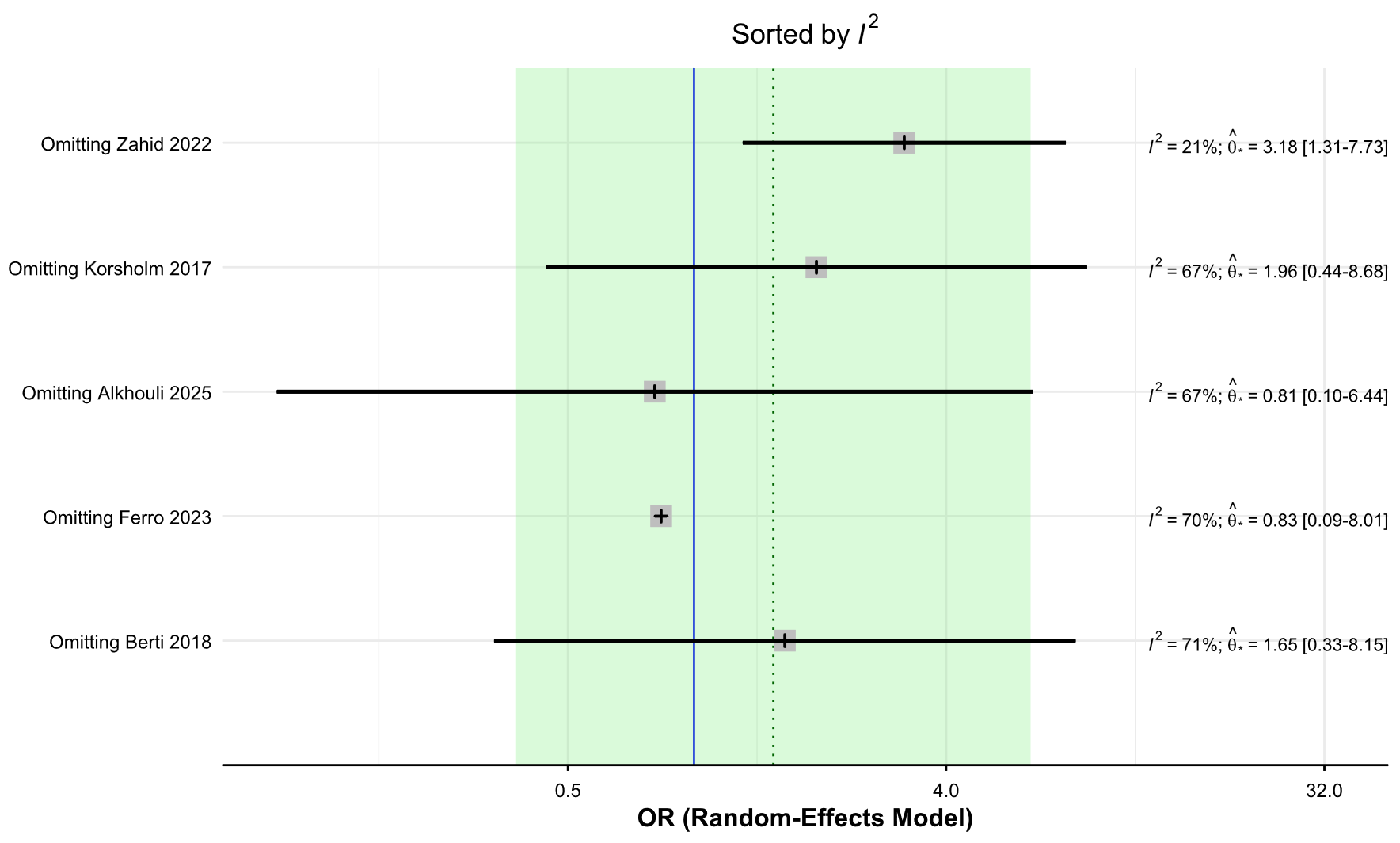


Supplemental Figure 41, Leave-one-out sensitivity analysis of unadjusted ischemic stroke.


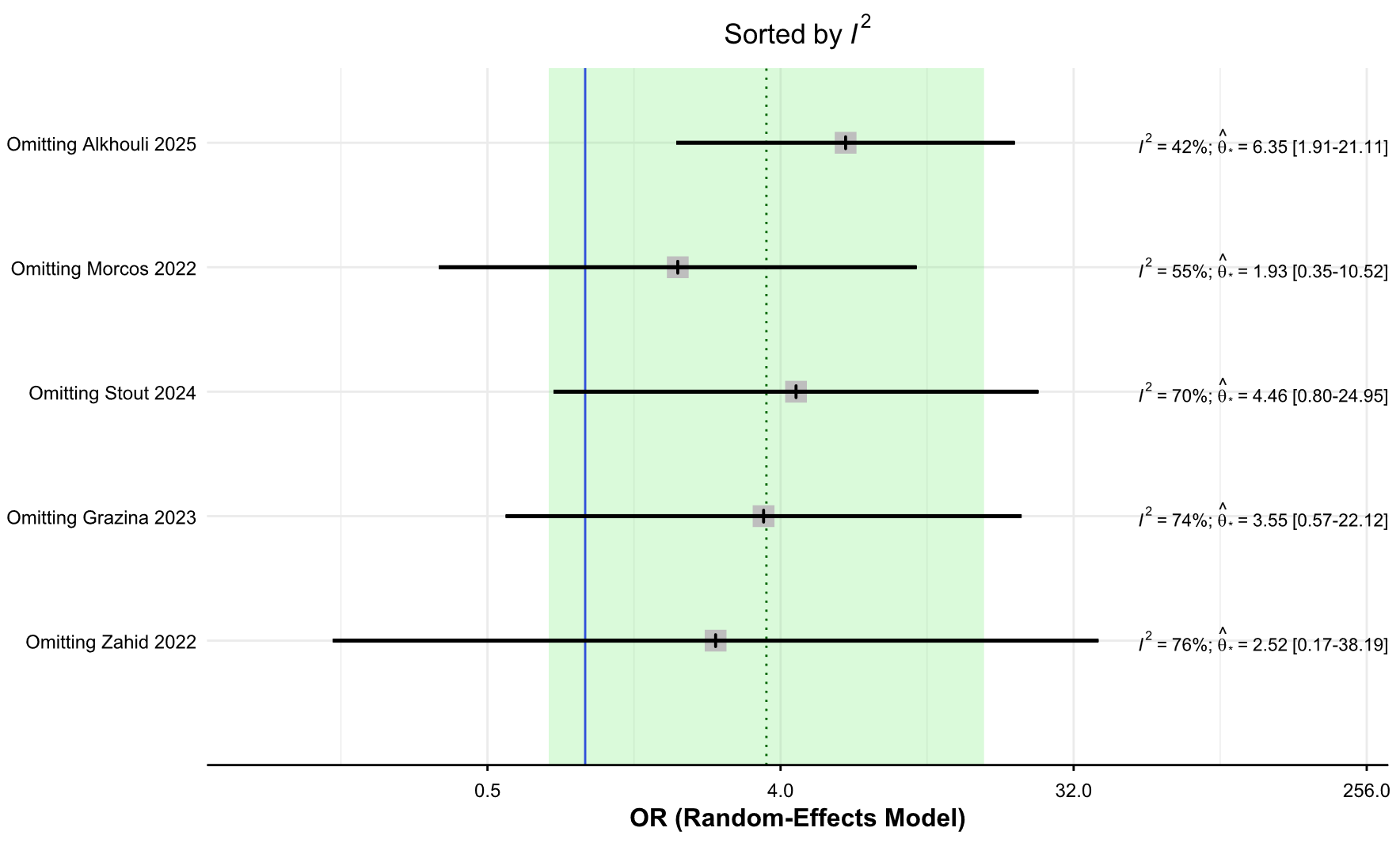


Supplemental Figure 42, Leave-one-out sensitivity analysis of unadjusted cardiac arrest.
